# Proteomic Profiling Identifies Distinct Protein Patterns in Acute Myelogenous Leukemia CD34+CD38- Stem-Like Cells

**DOI:** 10.1371/journal.pone.0078453

**Published:** 2013-10-24

**Authors:** Steven M. Kornblau, Amina Qutub, Hui Yao, Heather York, Yi Hua Qiu, David Graber, Farhad Ravandi, Jorge Cortes, Michael Andreeff, Nianxiang Zhang, Kevin R. Coombes

**Affiliations:** 1 Department of Leukemia, The University of Texas M.D. Anderson Cancer Center, Houston, Texas, United States of America; 2 Department of Bioengineering, Rice University, Houston, Texas, United States of America; 3 Department of Bioinformatics and Computational Biology, The University of Texas M.D. Anderson Cancer Center, Houston, Texas, United States of America; Emory University, United States of America

## Abstract

Acute myeloid leukemia (AML) is believed to arise from leukemic stem-like cells (LSC) making understanding the biological differences between LSC and normal stem cells (HSC) or common myeloid progenitors (CMP) crucial to understanding AML biology. To determine if protein expression patterns were different in LSC compared to other AML and CD34+ populations, we measured the expression of 121 proteins by Reverse Phase Protein Arrays (RPPA) in 5 purified fractions from AML marrow and blood samples: Bulk (CD3/CD19 depleted), CD34-, CD34+(CMP), CD34+CD38+ and CD34+CD38-(LSC). LSC protein expression differed markedly from Bulk (n=31 cases, 93/121 proteins) and CD34+ cells (n= 30 cases, 88/121 proteins) with 54 proteins being significantly different (31 higher, 23 lower) in LSC than in either Bulk or CD34+ cells. Sixty-seven proteins differed significantly between CD34+ and Bulk blasts (n=69 cases). Protein expression patterns in LSC and CD34+ differed markedly from normal CD34+ cells. LSC were distinct from CD34+ and Bulk cells by principal component and by protein signaling network analysis which confirmed individual protein analysis. Potential targetable submodules in LSC included the proteins PU.1(SP1), P27, Mcl1, HIF1α, cMET, P53, Yap, and phospho-Stats 1, 5 and 6. Protein expression and activation in LSC differs markedly from other blast populations suggesting that studies of AML biology should be performed in LSC.

## Introduction

Acute Myelogenous Leukemia (AML) patients die of their disease when therapy fails to eradicate all the neoplastic cells, resulting in rapid (primary refractory) or delayed (relapse) regrowth of leukemic blasts. The discovery of leukemia stem-like cells (LSC) or initiating cells (LIC) generated the hypothesis that cells crucial for leukemia regrowth exist within the LSC pool rather than the bulk AML population[1]. If this hypothesis is true, improving therapy for AML will depend on understanding how the biology of the chemoresistant LSC differs from the chemosensitive non-stem leukemic cells. 

Most studies suggest that normal and leukemic SC have a CD34+/CD38- phenotype[1–4]. LSC are further distinguished from normal HSC by characteristics within the side population by flow cytometry[5]. Normal HSC are CD90+/CD123-low/CD117+//HLA-DR+ while LSC are CD90-/CD123+/CD117-/HLA-DR- and also express CLL-1, CD44, CD47 and CD96[6–9]. This phenotype definition is controversial; some studies have demonstrated LIC with specific molecular abnormalities (del 5q)[10] in this pool; another found mutation bearing (NPM1) cells within CD34- fractions[11]. Engraftment of human AML cells was highest (93%) from the CD34+CD38- fraction but also occurred with CD34+CD38+ cells (63%)[12], possibly related to the cytotoxic effects of CD38+ antibodies[13]. For childhood pre-B acute lymphocytic leukemia, LIC were found in all maturation stages including CD34+ and CD34- populations[14]. In myelodysplasia, SC also had a CD34+CD38- phenotype[15]. In AML, higher frequencies of CD34+CD38-, but not CD34+ cells was prognostically adverse[16] and intermediate, versus high, aldehyde dehydrogenase expression correlated with relapse[17,18], supporting the concept that LSC contribute to regrowth. 

It’s intuitive that HSC or LSC will have different patterns of gene/protein expression and activation, compared to more differentiated progeny that define their unique biology. Elucidation of the differences between LSC and HSC could identify therapeutic targets. Characterization of protein expression differences between LSC and HSC is therefore crucial; however, the rarity of LSC/HSC has hindered isolation of sufficient cells to permit detailed study. Xenogenic transplantation of human cells into non-obese diabetic/severe combined-immunodeficiency mice suggested a LSC frequency between 0.1% to 1%, and normal HSC are rarer (1:100,000 to 1:10 million)[1]. Non-xenogenic transplants of mouse leukemias into histocompatible mice suggest a frequency of 2-5% and that the microenvironment significantly influences their frequency[19]. If these frequencies are correct, then an assay requiring 1x10^6^ LSC would require a starting population of 1x10^8^ (1%) to 1x10^9^ (0.1%) cells. Similarly, generating 1x10^6^ HSC would require between 1x10^10^ (1:10,000) to 1x10^12^ (1:1,000,000) cells, a LSC dose equivalent to a complete stem cell transplant marrow harvest. Consequently studies of mRNA gene expression profiling (GEP) in stem cells have been limited to date. Noverhstern and colleagues isolated 38 hematopoietic compartments from normal cord blood and marrow, and identified transcriptional circuits regulating each compartment based on mRNA GEP[20]. They demonstrated HSC specific transcription modules, but also showed extensive overlap between HSC and committed myeloid and megakaryocyte-erythoid progenitors, subsets equivalent to CD34+CD38+ cells. GEP comparing HSC (7 samples using 15-65,000 cells) to AML LSC (16 cases, 50-150,000 cells) demonstrated multiple differences, notably in the Wnt and MAP Kinase pathways[21]. Using CD34+ cells mRNA GEP revealed distinct subtypes of therapy related AML but comparison to normal HSC was not performed[22]. Another group compared LSC to non-LSC using mRNA GEP and observed 409 differentially expressed genes; a third of which were found in normal HSC[23].

Transcript expression may not accurately reflect protein expression (affected by mRNA translation, transcript degradation kinetics and protein degradation rates) or activation (e.g. post-translational modifications)[24–26]. Since protein activity regulates signaling in LSC, studying protein expression and activation is crucial to fully understand LSC biology, however, proteomic profiling of AML LSC has not been reported. We performed proteomic profiling of bulk leukemic cells using Reverse Phase Protein Array (RPPA), demonstrating the existence of recurrent patterns of protein expression that are frequently prognostic[27,28]. Since RPPA requires only 2x10^5^ cells, we hypothesized that we could use RPPA to assess protein expression differences between Bulk, CD34+ and the immature phenotype CD34+CD38- LSC containing populations. Herein, we present proteomic profiles and a network analysis based on 121 antibodies, demonstrating that expression patterns and pathway utilization in CD34+CD38- differ markedly from CD34+ or Bulk cells, and these differences suggest therapeutic avenues for targeting LSC.

## Methods

### Patient Population and Subset Isolation

 Sequential bone marrow specimens (N=85, 29 diagnosis, 56 relapse) or peripheral blood samples (N=22, 14 diagnosis, 8 relapse) were collected from 73 patients with newly diagnosed or relapsed AML (excluding acute promyelocytic leukemia,) evaluated at The U.T. M.D. Anderson Cancer Center between January 2007 and March 2008. Samples and written informed consent were acquired during routine diagnostic assessments per the regulations and protocols (Lab 01-473) approved by the M.D. Anderson Cancer Center Investigational Review Board (IRB) and those of the Declaration of Helsinki. Analysis occurred under IRB-approved protocol (Lab05-0654). Samples were enriched for leukemic cells by ficoll separation yielding a mononuclear fraction which then underwent magnetic antibody based sorting (MACS, Miltenyi-Biotec, Auburn CA) to produce five different population subsets. To produce leukemia enriched fractions (Bulk) cells underwent CD3/CD19 depletion, removing contaminating T and B cells. All remaining cells underwent CD34 positive selection to yield CD34- and CD34+ fractions. If sufficient numbers were available, the CD34+ cells then underwent CD38 depletion, after the removal of the CD34+ beads using the Miltenyi release reagent, to produce CD34+CD38+ and CD34+CD38- (LSC) fractions. The samples were normalized to a concentration of 1x10^4^ cells/μL and whole cell lysates prepared as described[29]. Sample yields and recovery details are shown in Table 1. Not all subsets could be produced in all cases. This median yield was 5.6x10^6^ CD34+ cells and 200,000 LSC from 102 and 31 samples respectively, averaging ~1% of the starting material (yields for individual cases are shown in Table S1 in File S1). The median WBC (p = 0.28), percent blood blasts (p=0.93) or percent bone marrow blasts (p=0.83) did not differ between samples from which we could obtain all fractions, compared to those that only underwent CD34+ selection. Purity was measured by flow cytometry on test samples during procedure development and was consistently >85% (Figure S1 in File S1). Low CD38 expression in the LSC samples was confirmed by GEP in a subset of cases. The associated demographics and clinical features are described in Table S1 in File S1. Due to sample size limitations we did not attempt to make correlations with clinical features (FAB, cytogenetics, FLT3 mutation status) or outcome. This population lack samples from the 10-15% of AML cases that are CD34 negative. As normal controls, 1x10^6^ CD34+ cells were collected from 10 different unstimulated normal bone marrow donors (AllCells, Emeryville, CA http://www.allcells.com/). Use of these were approved by the M.D. Anderson Cancer Center Investigational Review Board (IRB) under IRB-approved protocol (Lab05-0654)

**Table 1:** Sorting statistics.

		Median	Average	Standard Deviation	Minimum	Maximum
CD34 Sort	# Sorted	4.00E+07	7.93E+07	9.58E+07	5.00E+06	4.50E+08
	Yield 34+	5.60E+06	1.16E+07	1.70E+07	2.00E+05	1.10E+08
	Yield 34-	1.80E+07	4.95E+07	7.54E+07	7.00E+05	4.70E+08
	% 34+	21.10%	29.70%	27.20%	0.70%	96.20%
	% 34-	78.90%	70.30%	27.20%	3.80%	99.30%
CD38 Sort	# 38 sorted	6.40E+06	1.23E+07	1.39E+07	1.00E+06	8.00E+07
	Yield 34+, 38-	2.00E+05	5.25E+05	1.25E+06	1.00E+05	9.60E+06
	Yield 34+, 38+	4.00E+06	8.17E+06	1.18E+07	2.00E+05	8.60E+07
	% 34+/38-	5.00%	8.20%	9.50%	0.20%	60.00%
	% 34+/38+	95.00%	91.80%	9.50%	40.00%	99.80%
	overall SC %	1.00%	2.60%	4.20%	0.10%	28.30%

### RPPA Methodology

Proteomic profiling was performed using RPPA as described[27,28,30]. Briefly, samples were printed in 5 serial dilutions along with normalization and expression controls, with fractions from each patient clustered together. Most samples (n=345) were printed in replicate, but some with low cell number (n=31) were printed once. Pearson correlation coefficients of duplicated samples within arrays had mean, median, and standard deviations of 0.84, 0.87, and 0.09, respectively. Slides were probed with 121 validated (validation process described in[28]) primary antibodies, detecting total, phospho or cleaved proteins, and a secondary antibody to amplify the signal, and a stable dye is precipitated. The antibodies used, along with the manufacturer, catalog number, primary and secondary antibody concentrations are listed in Table S2 in File S1. A “Rosetta Stone” of antibody and protein names used in online databases (HUGO and MiMI) and the RPPA is included as Table S3 in File S1. The stained slides were analyzed using Microvigene® software (Vigene Tech, Carlisle, MA). 

### Statistical Analysis

 Relative protein concentrations in log_2_ scale were estimated by fitting a common logistic response curve using all sample dilution series within an array[27,31]. The algorithm was implemented in a R package, *SuperCurve*. A topographical normalization[32] followed by a median polish procedure was used to account for within-array background staining and sample loading variations.

 Paired t-tests and Wilcoxon signed-rank tests were applied to test the null hypotheses of no differential expressions between two cell types, e.g. CD34+CD38- vs. bulk, for any protein. A beta-uniform mixture model was used to control the false discovery rate (FDR)[33]. Two-way hierarchical clustering with Euclidean distance and Ward’s linkage rule was performed to explore the multivariate structures. The robustness of the numbers of groups resulted from the clustering analyses was examined by a bootstrapping method with 200 iterations[34]. Principal component analyses were employed to further examine the classifications of cell types based on multiple protein expressions. Associations between sample clusters and disease status (or sample source) were assessed by Fisher’s exact tests. 

 To compare leukemic and normal CD34+ cells, the lower and upper thresholds of protein expressions for normal CD34+ cells were calculated as the mean expression ± 1.96 × standard deviation (95% confidence interval). Analyses were performed using R statistical software with in-house developed packages including *SuperCurve* (http://bioinformatics.mdanderson.org/Software/OOMPA/). 

### Network Analysis

To supplement the above statistical analysis, we performed a network analysis. We identified pairs of proteins where the relative expression in CD38+CD38- cells was distinct from that of bulk or CD34+ cells[35]. To analyze the matrix of relative expression levels, we applied a t-test comparison for all possible pairwise combinations of the 121 proteins in the dataset (7260 pairs). The p-value of each protein pair from CD34+CD38- cells was calculated relative to those of bulk and CD34+ using the standard t-distribution. The resulting differences in CD34+CD38- vs. Bulk paired protein expression, for pairs statistically different at a p-value of α = 10^-10^, are shown by protein function . Patients and corresponding protein pair expression values in CD34+CD38- vs. Bulk were clustered by two-way hierarchical clustering with Euclidean distance and centroid linkage. Finally, from the identified sets of statistically different protein pairs, we built a network representation. Protein pairs were connected by joining overlapping proteins (nodes). Edges are our initial hypothesis of probabilistic interactions between identified proteins. Analysis utilized Matlab (MathWorks) and results are graphically represented using Cytoscape .. Within Cytoscape, we queried public databases to establish known networks for the proteins pairs identified as significant. The database sources used in these queries were all those available through the MiMI Plugin 3.0.1 (including protein-protein interactions (PPI) and signaling networks: BIND, CCSB, DIP, GRID, HPRD, IntAct, MDC, MINT, KEGG, PubMed, and reactome) restricted to human protein data,[36]. Query results showed how identified proteins were known to interact directly (PPI) or through transcriptional signaling. By highlighting proteins from our dataset, we could see where previously known interactions may occur. To identify possible subnetworks of highly connected proteins within the larger graphs, we used the MCODE Cytoscape Plugin, detecting densely connected regions through vertex weighting as a function of local neighborhood density[37,38]. 

## Results

**Figure 1:**
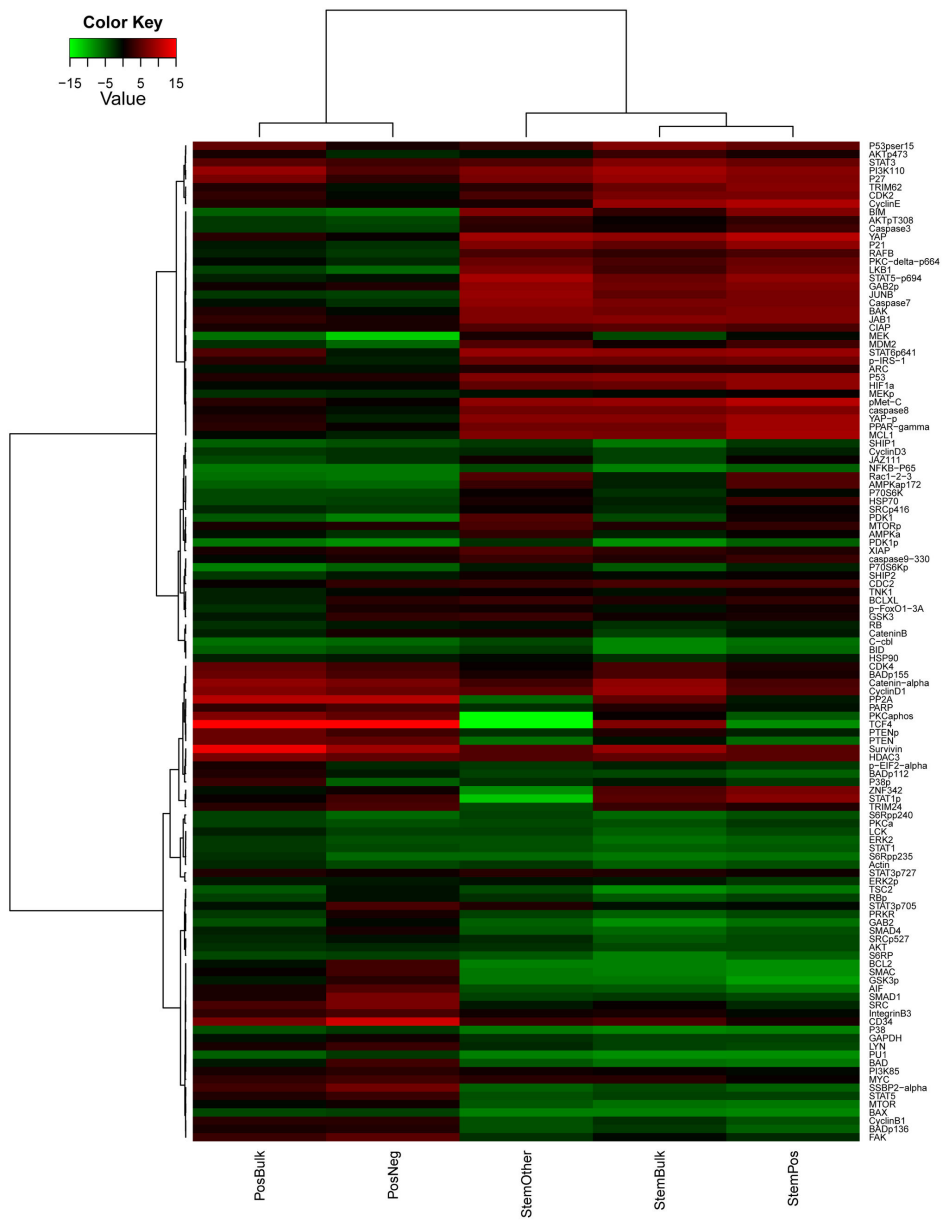
Summary of t-test results for individual proteins. The 5 cell subsets are labled as follows along the bottom: Stem = CD34+CD38-, OTHER = CD34+CD38+, POS= CD34+, NEG=CD34+ and BULK= Leuekmia enriched CD3/CD19 depleted cells. These are combined to show the comparison made, e.g. “PosBulk” is a comparison of CD34+ vs Bulk, “StemOther” is a comparison of Stem cells to CD34+CD38+ cells, etc. Proteins are clustered based on the t-statistics from pairwise comparison. Each column has data for the comparison between two subset populations listed at the bottom. Black values represents a t-statistic of zero. Red values are higher in the first of two groups named at the bottom of the column, and green values are higher in the second group. The t-statistic was calculated in each of pairwise comparisons per protein and the range of t-statistics is [-15.4, 22.7]. The values beyond [-15, 15] were chopped for the display purpose only.

**Figure 2:**
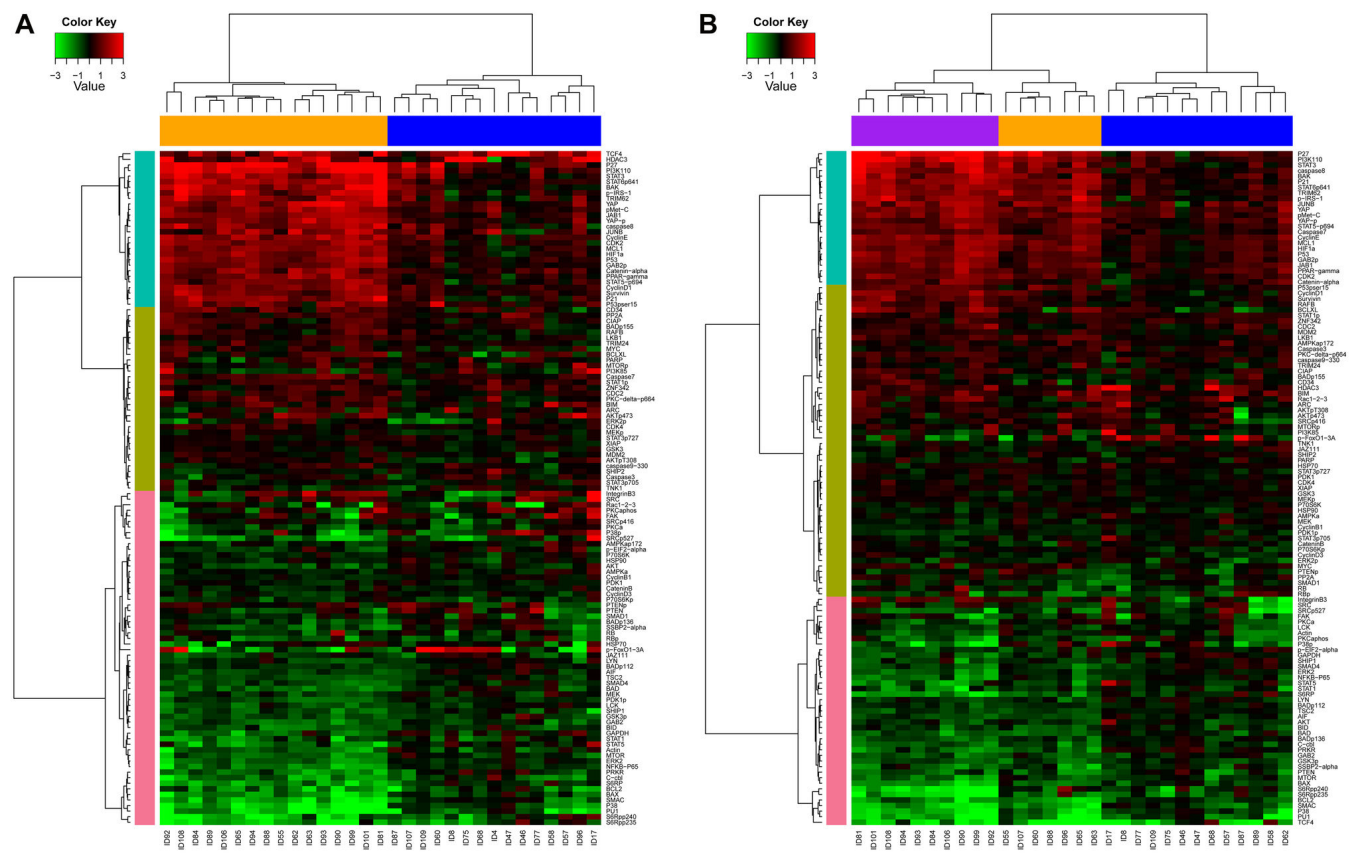
Two-way hierarchical clustering of paried differences between A) CD34+CD38- cells and Bulk cells and B) CD34+CD38- cells and CD34+, using normalized data. Case numbers are listed along the x-axis and protein names along the y-axis. Colors in the heatmap represent log ratios of protein expression in paired samples, with black representing 0, pure red representing +3, and pure green representing -3. Data beyond these bounds was truncated for display purposes. Comparable figures for CD34+ vs. BULK cells, CD34+ vs. CD34- and CD34+CD38+ vs. CD34+CD38- cells are shown as Figures S2C, D and E in File S1.

**Figure 3:**
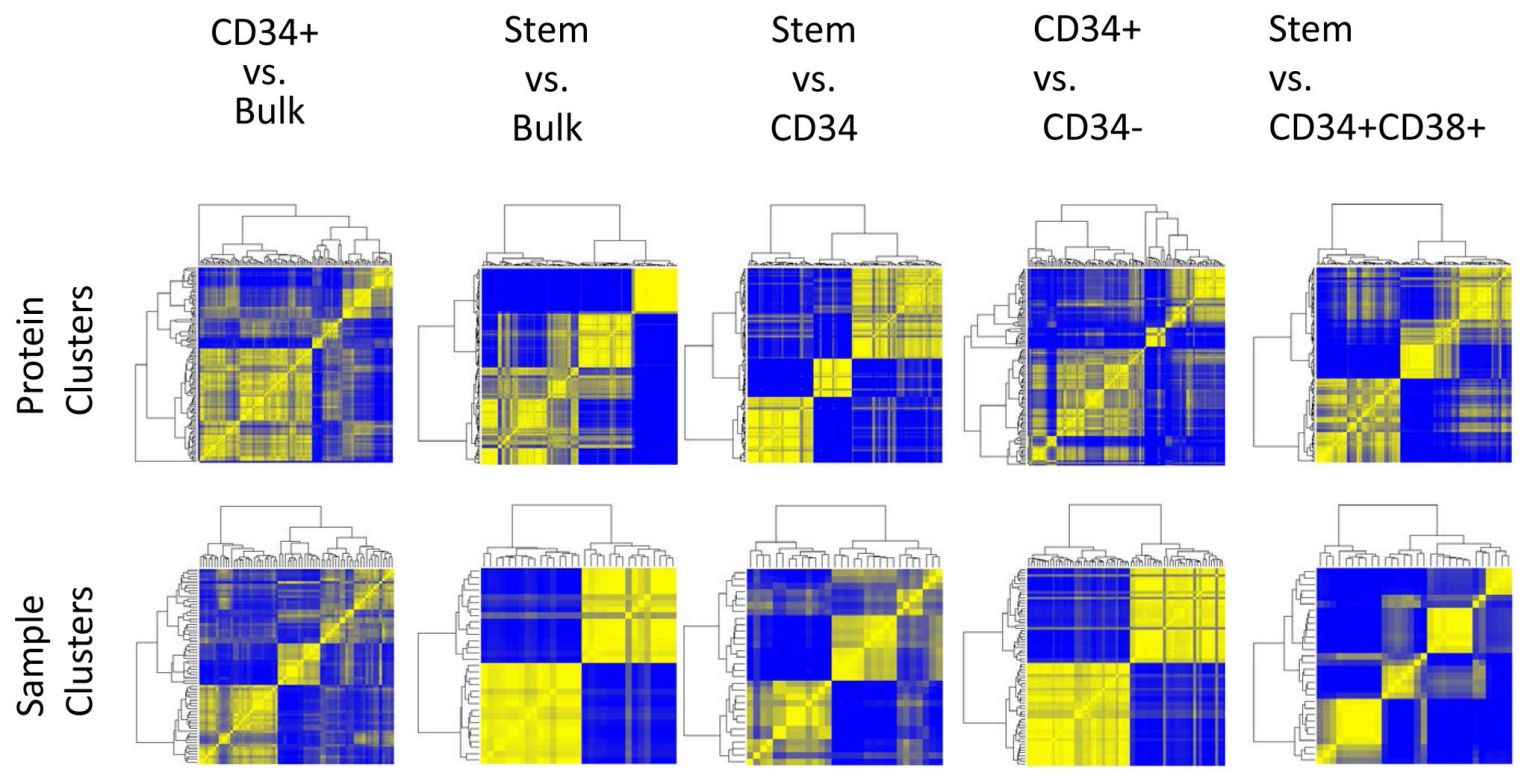
Reproducibility of Protein clustering (top row) or Sample Clustering (bottom row) for the 5 different subset comparisons. Reproducibility was assessed by reclustering 200 bootstrap datasets. Color scale ranges from pure blue (pairs of proteins that never cluster together) to pure yellow (pairs of proteins that cluster together 100% of the time).

**Figure 4:**
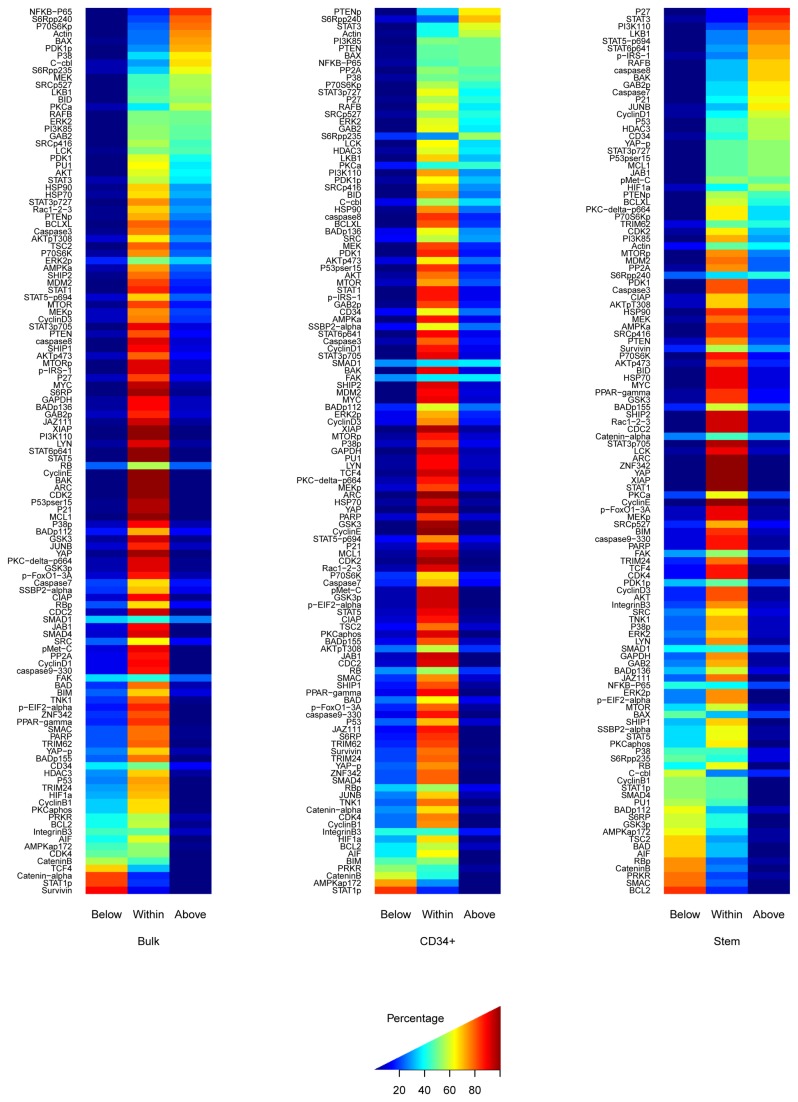
Comparison of expression in Bulk, CD34+ and LSC to that of normal CD34+ bone marrow cells. The percentage of BULK (Left), CD34+ (middle) or CD34+CD38- cells (right) samples that were below, within, or above the range defined by normal CD34+ cells is shown. Proteins are ranked so that those with the highest (lowest) expression relative to the normal CD34+are at the top (bottom).

Protein expression levels for all 121 antibodies were compared between the five different AML subsets including two intra-subset comparisons: CD34+ vs. CD34- and CD34+CD38+ vs. CD34+CD38- and three inter-subset comparisons: CD34+CD38- vs. CD34+, CD34+CD38- vs. Bulk and CD34+ vs. Bulk. The significance of differences was assessed using the Wilcoxon signed-rank tests and paired t-tests and two FDR thresholds, 1% and 10% (Table 2), and the number of comparisons shown in Table S4 in File S1. The t-test results for the 5 comparisons are summarized in Figure 1. Unbiased hierarchical clustering was performed for each comparison (Figure 2A & B and Figure S2C, D, & E in File S1). Perturbation bootstrap clustering was performed to test the robustness of clustering of proteins or patients for each comparison (Figure 3). Within each comparison, disease status (diagnosis or relapsed) and sample source (blood vs. marrow) were not statistically significantly associated with any protein or sample cluster (Table 2). The expression of each protein within the Bulk, CD34+ or LSC compartments relative to normal CD34+ cells is shown in Figure 4. 

**Table 2:** Summary of differences by False Discovery Rate.

		# Significantly different proteins				# of clusters			
		False Discovery Rate							
		0.1		0.01					
Comparison	#	Wilcoxon signed-rank	T-Test	Wilcoxon signed-rank	T-Test	Protein	Samples	Disease status	Blood vs. Marrow
Stem vs. Bulk	31	121	120	95	93	3	2	P= 0.72	P=1
Stem vs. CD34+	30	119	109	89	88	3	3	P = 0.11	P= 0.87
Stem vs. CD34+CD38+	30	118	118	96	97	3	4	P= 0.10	P= 0.18
CD34+ vs. CD34-	71	114	105	74	73	5	2	P= 1	P= 0.73
Bulk vs. CD34+	69	110	104	68	65	4	4	P= 0.12	P=0.81

### Stem cells vs. CD38+, CD34+ or Bulk cells

Regardless of whether CD34+CD38- are compared inter-subset to Bulk or CD34+ cells or intra-subset to CD34+CD38+ cells, the majority of proteins show significantly different expression in CD34+CD38- compared to either Bulk, CD34+ or CD38+ CD34+ cells (95, 89, and 96 proteins, FDR of 1%). As shown in Figure 1 the patterns of change are consistent in both the direction and the magnitude of significance in these 3 comparisons. The proteins consistently formed three clusters, separating into groups comprised of proteins with much higher, somewhat higher or much lower expression (Figure 2). These protein clusters were highly reproducible in the perturbation bootstrap clustering (Figure 3). Patients divided into 2 or 3 clusters when clustering was performed based on the similarity of patterns of differences between LSC and Bulk or CD34+ cells (Table 2) and patient clustering was again highly robust. Thus, protein expression in CD34+CD38- is significantly different from Bulk, CD34+ or CD34+CD38- cells. 

### CD34+ cells vs. Bulk and CD34- cells

CD34+ cells showed very different protein expression from either Bulk, or CD34- cells, with 68 and 74 proteins being significantly different (FDR=1%). The patterns of difference and magnitude were similar in both CD34+ comparisons (Figure 1). There were more protein clusters (4 vs. Bulk, 5 vs. CD34-), and while the protein clustering remained very robust it was not as strong as in the LSC comparisons. Patients formed 4 clusters for CD34+ vs. Bulk, with 2 being very robust and 2 less robust. Differences between CD34+ and CD34- divided patients into two clusters that were extremely robust. Thus CD34+ cells have a markedly different protein expression profile than either CD34- or Bulk leukemic cells.

### Principal Component Analysis

**Figure 5:**
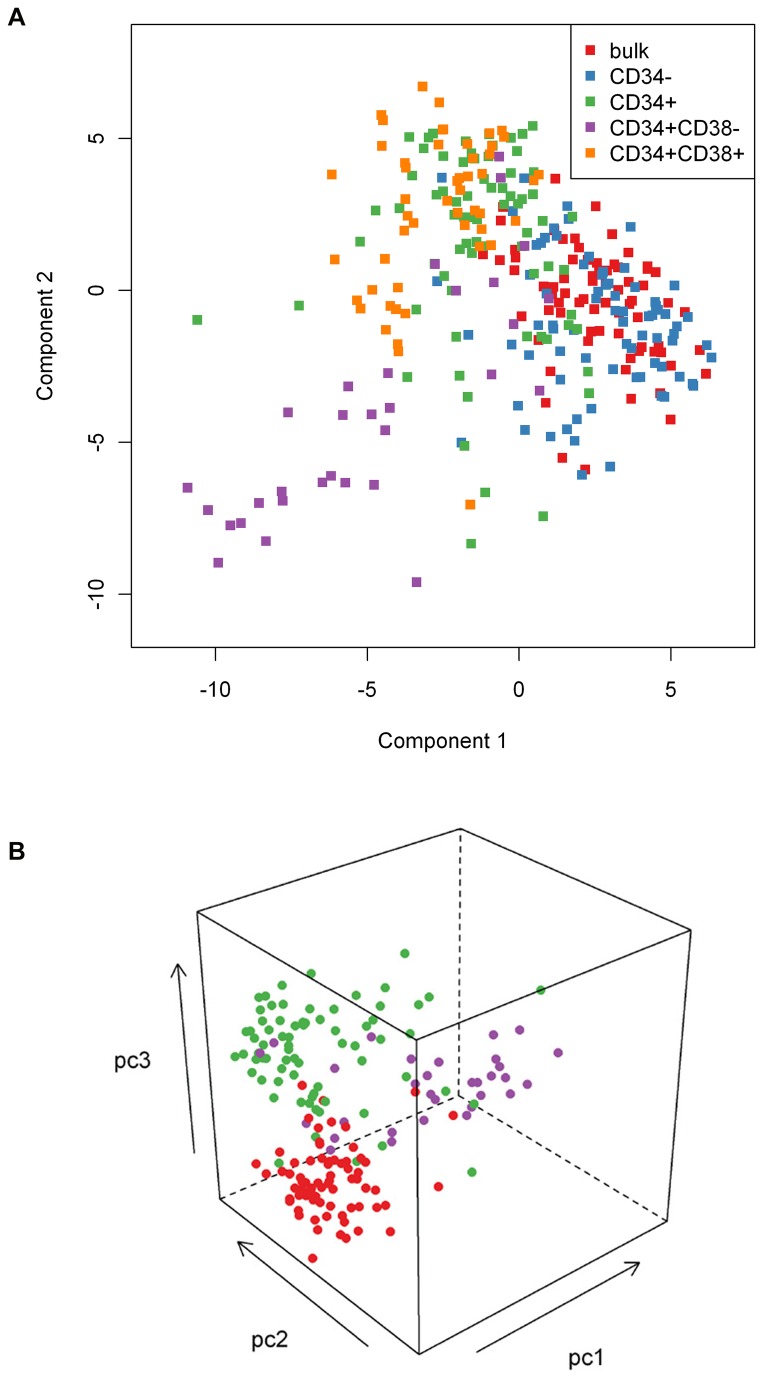
Principal component analysis demonstrates that LSC, CD34+ and bulk cells are distinct. Panel. A) Demonstrates separation along principal components 1 (x-axis) and component 2 (y-axis) for the 5 subgroups of samples studied. From the spatial separation it is clear that CD34+ cells (green) occupy a separate space from CD34- cells (blue) from which they were derived, which largely overlap with the unfractionated Bulk population (red). Similarly after the CD38 sort the CD34+CD38+ cells (orange) largely overlay the CD34+ cases, from which these cells were separated, and the CD34+CD38- population (purple) occupies a separate space. B) Principal component analysis based on components 1, 2 and 3 shows clear separation between Bulk (red) CD34+ (green) and CD34+CD38- (purple) samples.

As a measurement of global differences or similarities between the 5 different AML subsets principal component analysis was performed. The first three PCs represent 18%, 16%, and 12% of total variance, with each remaining PC representing below 7% of the variance. The steady march of differences associated with increasing purification is easily visualized in Figure 5A. As might be expected from these results, LSC occupied a unique space compared to CD34+ or Bulk cells (Figure 5B) and the Mahalanobis distance between the mean of CD34+CD38- and Bulk population is 3.55. The distances are 3.17 and 2.36 between CD34+ and Bulk and between CD34+ and CD34+CD38-, respectively. 

### Summary of proteins with differential expression

We next asked which proteins had higher or lower expression in CD34+CD38- cells. Because we analyzed multiple antibodies (n=121) we used a Bonferroni corrected threshold of 0.0004 (=0.05/121) to determine significance. Table 3 shows the 57 proteins that demonstrated significantly different protein expression, either higher or lower, in CD34+CD38- compared to both CD34+ cells and Bulk cells at the Bonferroni threshold. When CD34+ cells were compared to Bulk cells, 34 of these same proteins were significantly different in the same direction (* in Table 3). Proteins over expressed in CD34+CD38-, and/or the pathways they implicate, are potential therapeutic targets that might selectively target LSC. Proteins that were not different are listed in Table S5 in File S1. 

**Table 3:** Summary of proteins that were consistently differentially expressed between LSC and both Bulk and CD34+ cells.

Functional Class	Apoptosis	Cell Cycle	Expression	Proliferation	Signalling	Miscellaneous
Higher in LSC	*Bak	*Cyclin D1	*HDAC3	**GAB2p**	**METp**	*PP2A
	**Bim**	^#^ CDC2	**JAB1**	**HIF1α**	*αCatenin	
	**Caspase7**	**CDK2**	**JUNB**	* *LKB1*	*PI3K110	
	**Caspase8**	**CyclinE**	**P53**	**IRS1p**	**Stat1p**	
	**CIAP**	**P21**	*P53pSer15	**PPARγ**	*Stat3	
	**MCL1**	*P27	*TCF4		**Stat5p694**	
	*Survivin		**YAP**		*Stat6p641	
			**YAPp**			
			**ZNF342**			
	**Badp155**	*CDK4	**Trim24**			*CD34
Lower in LSC	**AIF**	Cyclin B1	*NFκB-P65	*Gab2	^#^ AKT	**Actin**
	**BAD**		**mTOR**	**GSK3p**	^#^ * Β-Catenin*	*GAPDH*
	BADp112		^#^ PRKR	**LCK**	**cCbl**	* ^#^ *SHIP1*
	BADp136			^#^ *P70S6K*	**ERK2**	
	*BAX			*RBp	*Lyn*	
	**BCL2**			*S6RP	*P38	
	*BID			*S6RPp235	*PDK1p	
	**SMAC**			*S6RPp240	* ^#^ *PKCα*	
				*SMAD1*	**Stat1**	
				**SMAD4**	Stat5	
				^#^ *SRCp527*		
				*SSBP2α		
				*TSC2	* MEK	
	* Jaz111	CyclinD3			PDK1	HSP90

All proteins in **bold** were significant at a Bonferroni corrected p-value of p <0.00041. Those in plain type were significant at a p value <0.001 but >0.00041, those in *italics* were signficant at a p-value <0.01 but > 0.001. Proteins that are underlined were only significant when LSC were compared to Bulk cells but not when LSC were compared to CD34+ cells. Proteins preceeded by a ^#^ were significantly different, at the P <0.00041 level, when LSC were compared with Bulk cells. Proteins preceeded by a * were also significant when CD34+ cells were compared to Bulk cells. A lower case p after a protein name indicates “phosphorylated” protein, in some cases the affected amino acid is noted.

### Comparison of protein expression to normal CD34+ cells

Proteins overexpressed in CD34+ or LSC relative to their normal counterparts might be leukemia specific therapeutic targets. To identify proteins fitting this profile we compared expression in the Bulk, CD34+ and CD34+CD38- to normal CD34+ cells (Figure 4) as sufficient normal HSC were unobtainable. The proteins in CD34+CD38- that showed the highest percentage of cases with expression significantly above that of the normal CD34+ cells included P27, STAT3, PI3KP110, LKB1, STAT5p, STAT6p, IRS-1p, RAFB, Caspase 7, Caspase 8, GAB2p,P21, P53 and JUNB, all of which had >60% of cases showing higher than normal expression. Proteins that were lower than normal in >60% of cases include BCL2, β-Catenin, SMAC, RBp, PRKR, AIF, BAD and TSC2. Protein expression in CD34+CD38- showed partial correlation with published mRNA-GEP datasets (see Supplemental Materials in File S1), however the immunophenotype used to define the LSC differs between these studies. 

### Network Analysis

#### Significantly different protein pairs and subnetworks

**Figure 6:**
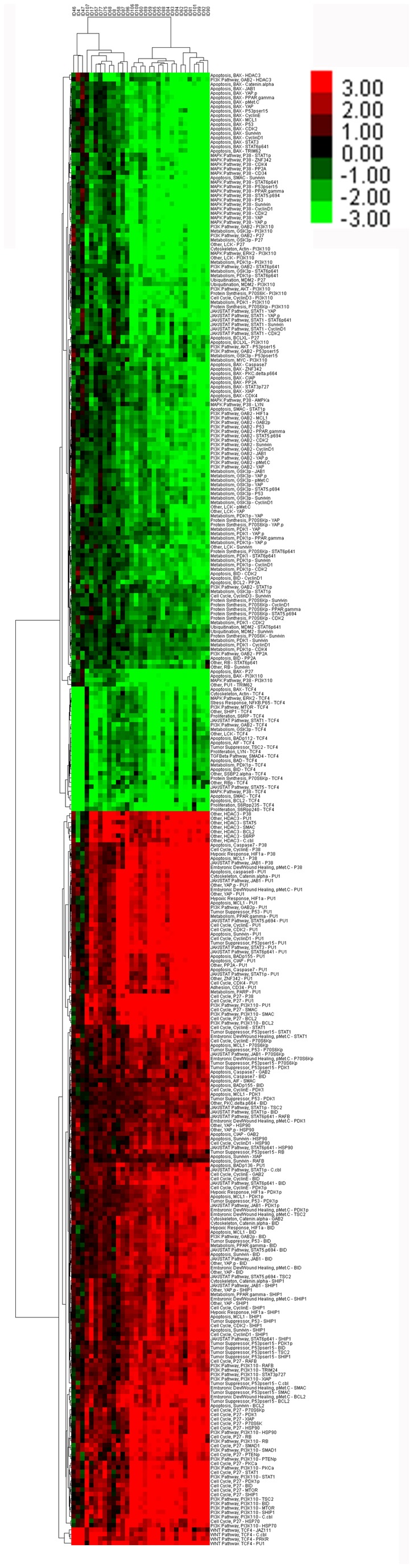
Hierarchical cluster analysis of paired proteins. Clustering based on all protein pairs for CD34+CD38- vs. Bulk (p ≤ 10^-10^). The same two overall clusters appeared in the paired protein analysis compared with the individual protein comparisons (see Figure 2A) except for two patients (ID 59 and ID 60). Cluster analysis was also performed on patients grouped by individual protein pathways (e.g., apoptosis, PI3K), and two or three different clusters emerged (See Figure S3 in File S1).

**Figure 7:**
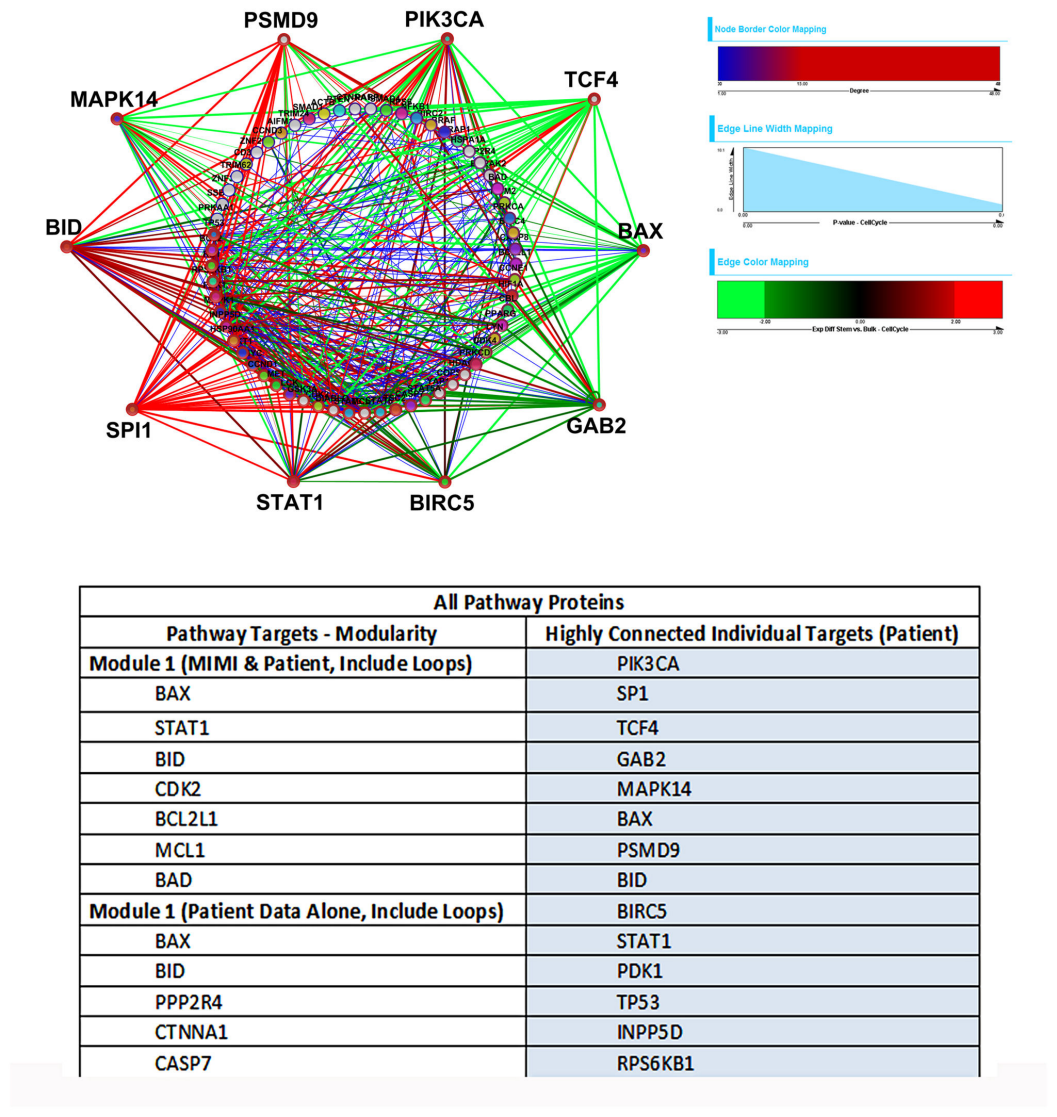
Global networks identified. The overall network of the most highly differentially expressed protein pairs between CD38+CD38- vs. Bulk cells (p-value ≤ 10^-10^) based on the entire dataset is shown. The green to red edges represent relative expression levels from the RPPA data, and show connectivity predicted by the t-test protein pair comparisons between CD34+CD38- vs. Bulk cells; to interpret the edge colors, please see Figure S3 in File S1 which displays the ordering of the protein pair ratios depicted in this network. Edge width corresponds to relative p-values, with wider edges having lower p-values. Blue edges indicate PPI and signaling interactions from public databases. Node border color and width correspond to the number of protein connections. Individual groups based on protein function are shown separately in Figure S4 in File S1, Subnetworks of highly interconnected proteins found from modularity analysis are listed and the associated proteins with the greatest connectivity (≥ 15 connections) are listed in the accompanying table including those identified by online datasets (left) and those from patient data from this dataset (right). A legend for the node colors, which correspond to known signaling pathways associated with the proteins from available public databases, is included as Table S6 in File S1.

Using the complementary network biology approach we observed that 55% (3992) of the 7260 possible protein pairs were significantly different, at a p-value of 0.001, between CD34+CD38- and bulk cells while 49% (3584) were different between CD34+CD38- and CD34+ cells (Table 4). The number of significantly different pairs was markedly less if the dataset is scrambled (Table 4). The associated differences in CD34+CD38- vs. Bulk paired protein expression for statistically different pairs (α = 10^-10^), are shown individually (Figure 6) and by protein function (Figure S3 in File S1). Figure 7 shows the resulting network from all the differentially expressed proteins, the subnetwork of proteins with the highest interconnectivity, and individual proteins with the highest degree of overall connections. The central role of increased Pu.1 (SPI1) and P27 (PSMD9) connectivity to multiple pathways is notable. Proteins grouped by function are shown in Figure S4 in File S1, along with their associated subnetworks identified by MCODE modularity analysis. Key findings from the individual networks include the decreased relative expression of proapoptotic pathways associated with BAX; decreased TCF4 in connection with apoptotic proteins and mixed TCF4 in the proliferative submodule; overall lower MAPK signaling through MAPK14; increased histone deacetylase (HDAC3); overall increased HIF1α and PI3K expression; and increased P53 relative expression in CD34+CD38- cells vs. Bulk cells.

**Table 4:** Significantly different pairs by p-value.

	Protein Pairs Found Significantly Different (% of 7260)				
p-value	CD34+CD38- vs. bulk	Scrambled CD34+CD38- vs. bulk	CD34+CD38- vs. CD34+	Scrambled CD34+CD38- vs. CD34+	Number of Shared Protein Pairs (2)
**10^-12^**	**34 (0.5%)**	**0 (0%**)	**1 (0%**)	**0 (0%**)	**0**
**10^-11^**	**135 (2%)**	**0 (0%**)	**2 (0%**)	**0 (0%**)	**0**
**10^-10^**	**331 (5%)**	**0 (0%**)	**46 (0.6%)**	**0 (0%**)	**16 (35%)**
**10^-8^**	**1160 (16%)**	**0 (0%**)	**436 (6%)**	**0 (0%**)	**301 (69%)**
**6.89 x 10^-6^ (1)**	**2746 (38%)**	**0 (0%**)	**2041 (28%)**	**0 (0%**)	**1684 (83%)**
**0.001**	**3992 (55%)**	**6 (0.1%)**	**3584 (49%)**	**5 (0.1%)**	**3062 (85%)**

(1) This is the bonferroni corrected p-value based on 7260 possible comparisons.

(2) Significantly different pairs between both CD34+CD38- and Bulk and CD34+CD38- and CD34+ (% is relative to the total number of significantly different pairs found for CD34+CD38- vs. CD34+ at each p-value).

## Discussion

Stem cells are thought to be the reservoir from which chemoresistance and regrowth of leukemia arise, making understanding LSC crucial to improving therapy. The inability to purify sufficient numbers of LSC has limited their study to gene expression profiling or single protein analyses, typically in small cohorts. Similar to other studies[20,21] we isolated a small number of CD34+CD38- cells, the fraction most frequently shown to contain LSC, median 200,000 cells, but we leveraged the sample sparing ability of RPPA technology to study the expression of 121 different protein epitopes, creating the first report of proteomic profiling of CD34+CD38-. The major observation is the stepwise differences in protein expression that becomes increasingly apparent as more purified samples are studied. The majority of proteins had significantly different expression between CD34+ cells and Bulk cells and between CD34+CD38- and any other subset. These differences were highly consistent in both direction and magnitude. There were very tightly clustered groups of patients with similar patterns between the purified group and the material it was purified from, for example the individual protein and network based analysis cluster 29 of 31 cases of Stem vs. Bulk identically (Figures 2A and 6). This consistency suggests that actual physiologic differences are being described. Although this patient population was heterogeneous, with respect to clinical features and disease status, the differences had similar magnitude in all subsets suggesting that these findings are generalizable across the entire population of AML cases.

This finding has implications regarding which cells should be used for studies of AML biology. DNA based events, including mutations, SNPs, DNA methylation and histone acetylation, are unlikely to be different between LSC and their downstream progeny (personal communication J.P. Issa), so any blast is theoretically suitable. In contrast, these data strongly argue that CD34+CD38- are a more suitable target population for protein studies. The use of LSC will provide insight into the biological differences that distinguish LSC from other populations, and could identify the therapeutically targetable idiosyncrasies of LSC. If the Bulk blast population is studied, the characteristics described are unlikely to accurately reflect the biology of the more clinically relevant LSC. Studies that utilize the more numerically available CD34+ blast will capture half of LSC and bulk cell differences, but will miss many changes found exclusively in LSC. Since mRNA-GEP based data also differ between LSC and other fractions it is highly likely that the same is also true for miRNA profiling. Consequently the accurate understanding of leukemia biology will require the integration of genetic (mutation) and epigenetic data obtained from any population of blasts (Bulk), while rare LSC should be triaged for use in miRNA, mRNA and proteomic profiling. 

There are technical caveats to consider. We were only successful in isolating the 200,000 CD34+CD38- required for the RPPA from ~ 60% of cases where a CD38 sort was attempted (37/63). In addition, there were 33 samples where no CD38 sorting was attempted, due to low CD34+ cell numbers (<8x10^5^). Our results could therefore be subject to an inclusion bias based on which samples could yield sufficient CD34+CD38-. Secondly, were the CD34+CD38- cells studied truly stem cells? Since additional LSC surface markers are described, additional sorts on these markers to generate further enriched populations of stem cells are theoretically possible. However additional sorting would further reduce cell number and preclude RPPA analysis. Newer technologies like mass cytometry might permit study of a reduced set of proteins in a smaller population of stem cells[39]. We believe that the stepwise and consistent nature of these changes suggest that these differences reflect the protein expression present in a LSC enriched population, even if the “true” stem cell is a further subpopulation of the cells studied.

This analysis showed differential protein expression in CD34+CD38- that could have therapeutic relevance. We observed that CD34+CD38- cells highly over-expressed Mcl1 while Bcl2 expression was significantly lower. Mcl1 is an essential survival factor for hematopoiesis in humans[40] and HSCs have very high Mcl1 expression[41]. FLT3 signaling increases MclL1 expression in both FLT3-WT and FLT3-ITD LSC. In ITD positive cases this occurred via STAT5 signaling and contributed to LSC survival[42]. Resistance to apoptosis is considered to be a hallmark of malignancy[43] so the discovery that three proteins (Mcl1, cIAP and Survivin) that mediate resistance to apoptosis are highly over-expressed in CD34+CD38- cells suggest that these might be therapeutically relevant targets. In contrast Bcl2 was shown to be under-expressed in CD34+CD38- cells. If this is a general phenomenon in other leukemias, this might explain the lack of therapeutic efficacy with Bcl2 antagonists in leukemia. Results from the network analysis show a synergistic but more complex picture of apoptotic signaling, with protein pair-specific changes, but an overall decrease in relative signaling through proapoptotic BAX, mixed anti-apoptotic signaling through PU.1 (SP1) and BID, and mixed, but generally decreased signaling for MAPK14 in LSCs (Figure 7; see Figures S3A and S4A in File S1 for the order of the protein pair ratio depicted in Figure 7). Other observations obtained from the network analysis include the central importance of cell cycle regulating proteins P27 (PSMD9) and PU.1 (SP1).

The normal bone marrow microenvironment is hypoxic[44] and the leukemia marrow even more so[45]. HIF-1α induces the chemokine CXCL12[46], which is expressed by stromal cells in normal and malignant marrow, and both HSC and LSC express the CXCL12 receptor CXCR4[47]. This CXCL12 loop serves to attract and keep HSC and LSC in the marrow[48]. Thus, in hypoxic conditions, HIF-1α expression would induce CXCL12 and attract LSC to protective marrow niches. We observed that HIF1α levels were significantly higher in CD34+CD38- cells in the individual protein analysis and had increased activity relative to its cosignalling factors MARK14 (P38) and SPI1 (Pu.1), supporting that this loop is active in LSC and that a hypoxic environment is relevant to LSC biology. Indeed, knockdown of Hif1α in LSC or in bone marrow stromal cells diminished leukemia growth in vivo[49]. These data support the hypothesis that chemotherapy agents that are preferentially activated in hypoxic conditions would have activity in AML[50].

 The differences observed in signal transduction pathway utilization also suggest potential therapeutically exploitable differences. Activation of Stats 1, 5 and 6 was noted to be increased in CD34+CD38- cells, relative to normal CD34+ cells or to CD34+ or bulk AML cells, based on both individual protein and network based analysis, suggesting potentiated activity through increased interaction with SPI1 (Pu.1). Using multiparameter flow cytometry we previously observed increased responsiveness of Stat activation pathways in AML cells to G-CSF or SCF[51]. In contrast, none of the RAS/RAF/MEK/ERK, MAPK14 (P38) or the AKT pathways were differentially expressed in LSC vs. CD34+ or bulk cells, or in CD34+CD38- cells vs. normal CD34+ cells in the individual protein analysis, however the network analysis did show a significant increase in PIK3CA (PI3K110) based interactions in several pathways. There are many small molecule inhibitors of each of these 4 pathways in clinical development, but these data suggest that those targeting the STAT pathways may show greater selectivity for LSC. Also notable is that all components of mTOR signaling (total MTOR, pS70S6K, S6K and proteins that are up/downstream of AKT: pPDK1, PDK1, GSK3b, TSC2) are lower in CD34+CD38- cells suggesting that mTOR inhibitors will spare LSC.

 The higher connectivity of Pu.1 among the CD34+CD38- cells fits in well with the already defined role of Pu.1 in AML. Pu.1 function may be deregulated in AML by mutation (7% in some studies, although not observed in the recent TCGA report) or by a SNP in an upstream enhancer (perhaps in 65% of humans) as well as by other fusion genes and down-regulation in mice can lead to leukemia and lymphomas[52,53]. In our RPPA on bulk leukemia cells in 511 newly diagnoses AML cases we observe that PU.1 expression was very heterogeneous in expression, with 30% of cases being above that of normal CD34+ cells and 26% being lower than normal CD34+ cells (data not shown). In the literature Pu.1 disruption has not been described as a universal finding in AML and our own data would suggest that this is not the case either. Furthermore the level of expression is prognostic, with those with less Pu.1 having an inferior outcome (manuscript in preparation). In this dataset Pu.1 expression did not differ from that of bulk cells (third from the bottom in Figure 2A) or from the CD34+ cells (one from the bottom in Figure 2). However in the network analysis Pu.1 levels were very highly correlated with protein expression in the CD34+CD38- cells, suggesting more of a role in the stem cell compartment, which fits with the data of others. Thus our data supports that of others, that Pu.1 is down-regulated in many, but not all cases and that it seems to have a role in stem cell maintenance.

Based on the finding of Eppert et al, that the presence of LSC-like gene expression profiles in bulk leukemic blasts is often observed and is prognostically adverse, we looked for similar findings at the protein level. In our comparison of LSC to bulk, we observed two clusters, one highly distinct and the other less different. Those that were less different might correspond to cases where the Bulk cells are more LSC like at the protein expression level. However we were unable to confirm that the presence of a LSC like proteomic signature in bulk blasts was prognostically adverse in a cohort of 511 AML patients (Supplemental Data in File S1). This may be due to the limited number of unselected protein studied here vs. the larger number of genes selected for inclusion in the signature derived from the LSC mRNA-GEP analysis. Alternatively, since all leukemias have stem cells, but not all leukemias are adverse prognostically, there must be favorable and unfavorable protein or mRNA based signatures. Since we merged all of these to define our protein signature we may have obscured the prognostic impact of having an unfavorable LSC like protein expression signature in bulk cells. This dataset was also weighted towards intermediate and unfavorable prognosis patients, hence the LSC protein expression signature might have only been present in a more homogeneous group of patients and therefore been non-predictive.

In summary we have demonstrated the ability to study protein expression in AML CD34+CD38- cells and these cells have markedly different protein expression patterns than either CD34+ or Bulk leukemic cells. These findings suggest that studies of protein expression, and by extension mRNA expression, should preferentially be performed in LSC to gain insight into biologically and therapeutically more relevant populations of cells. We have previously demonstrated that protein expression signatures exist in AML and are prognostic in AML [27,28]. We observed 7 signatures in bulk cells and saw three LSC signatures in this smaller cohort of 31 cases. It is likely that a larger number of signatures would be observed after studying a greater number of samples. These data suggest that a study of a significantly larger number of LSC samples from newly diagnosed AML cases is needed to define the protein expression patterns that characterize AML LSC and to determine the prognostic significance of different patterns in LSC. Such information would provide stronger leads for the selection of targeted therapies that are matched to the pathophysiology of the stem cells of an individual case. We are currently collecting samples for such a study. Two proteins, SPI1 (PU.1) and PSMD9 (P27), that were highly connected and differentially regulated were identified as potential LSC selective therapeutic targets, but this requires additional investigation. 

## Supporting Information

File S1 **Contains all supplemental information for this manuscript in one location.** Supplemental materials contains a comparison of RPPA and mRNA-GEP data. Table S1 contains the demographic and subset availability data for all cases. Table S2 lists the antibodies used in this study and details on primary and secondary antibody dilutions. Table S3 is a “Rosetta Stone” for the HUGO, Mimi and manufacturers antibody names . Table S4 lists the number of comparisons available between each subset. Table S5 lists proteins that were not different between LSC and bulk cells by protein function. Table S6 contains legends for the Cytoscape ™ figures. Figure S1 presents flow data showing the purity of the samples after sorting. Figure S2 is an expansion of regular Figure 2. It shows two way hierarchical clustering between C) CD34+ vs. Bulk cells, D) CD34+ vs. CD34- cells and E) Stem Cells and CD34+ cells. Figure S3 shows Network based clustering by protein functional category. Figure S4: Subnetworks for highly interconnected proteins for different cellular functions.
(PDF)
