# Supplementary material for: Proteomic Profiling Identifies Distinct Protein Patterns in Acute Myelogenous Leukemia CD34+CD38- Stem-Like Cells
Source: PLoS One. 2013 Oct 24;8(10):e78453. doi: 10.1371/journal.pone.0078453 (PMC3816767; doi:10.1371/journal.pone.0078453)

Proteomic Profiling Identifies Distinct Protein Patterns in Acute Myelogenous Leukemia CD34+CD38- Stem-Like Cells .

SUPPLEMENTAL MATERIALS

Comparison of RPPA to AML stem cell mRNA-GEP data

We queried how well the protein data correlated with mRNA-GEP based findings. Majeti et al[1] generated mRNA gene expression profiles from normal bone marrow HSC and AML LSC and identified genes and pathways that were differentially expressed. Starting with a subset of proteins where the levels in LSC were significantly above or below that of normal CD34+ cells in more than 50% of the cases, we compared that protein list to their mRNA-GEP findings (supplemental online dataset). Among 24 proteins significantly higher by RPPA, only 1 was higher by GEP, 4 were lower and 19 did not differ. Among the 18 proteins that were lower by RPPA, 10 were not significantly different by GEP, 5 were lower and 3 were higher. Caveats with this comparison is that many of the RPPA differences were in phosphorylated proteins (8 that were higher, 5 that were lower) but only total mRNA could be queried in the GEP dataset,. Also, there were different comparators, LSC to CD34+ in this dataset and LSC to HSC in their dataset. Nonetheless, poor correlation between protein expression and GEP has been noted in several other datasets and highlights the importance of studying protein instead of mRNA.[2-5]

Recently Eppert et. Al. [6] defined mRNA gene expression programs associated with normal and leukemic stem cells from CD34+CD38-, CD34+CD38+ and less frequently CD34-CDD38+ and CD34-CD38- populations of cells. They observed that prognosis was significantly worse when bulk AML cells had a gene expression program similar to that of either the normal or leukemic stem cells. We queried whether the same would be true for protein expression signatures. Using the data described above, principal component analysis was performed to define a signature, based on the

differential expression between CD34+CD38- and bulk cells of 88 proteins. This signature was then used to score 511 AML patients previously studied in a separate RPPA[7] that utilized bulk blasts for “stemness”. These patients were divided into sextiles to see if “stemness” was associated with overall survival or remission duration, but no obvious association was observed.

Reference List

1. Majeti R, Becker MW, Tian Q et al. Dysregulated gene expression networks in human acute myelogenous leukemia stem cells. Proc.Natl.Acad.Sci.U.S.A. 2009;106:3396-3401.

2. Gygi SP, Rochon Y, Franza BR, Aebersold R. Correlation between protein and mRNA abundance in yeast. Mol Cell Biol 1999;19:1720-1730.

3. Griffin TJ, Gygi SP, Ideker T et al. Complementary profiling of gene expression at the transcriptome and proteome levels in Saccharomyces cerevisiae. Mol.Cell Proteomics. 2002;1:323-333.

4. Washburn MP, Koller A, Oshiro G et al. Protein pathway and complex clustering of correlated mRNA and protein expression analyses in Saccharomyces cerevisiae. Proc.Natl Acad Sci U.S.A. 2003;100:3107-3112.

5. Greenbaum D, Colangelo C, Williams K, Gerstein M. Comparing protein abundance and mRNA expression levels on a genomic scale. Genome Biol. 2003;4:117.

6. Eppert K, Takenaka K, Lechman ER et al. Stem cell gene expression programs influence clinical outcome in human leukemia. Nat.Med. 2011;17:1186-1193.

7. Kornblau SM, Singh N, Qiu YH et al. Highly Phosphorylated FOXO3A Is An Adverse Prognostic Factor in Acute Myeloid Leukemia [abstract]. Blood 2009;114:166-167.

Supplemental Table 1. Subsets available for each sample and selected clinical and demographic features are shown. For the subsets a black box indicates that this subset was available. The percentage yield of CD34+ and CD34+CD38- cells as a percent of the starting material is shown. Samples that were excluded because of duplicate source, (blood and bone marrow), or for multiple dates (diagnosis and relapse) are noted. FAB= French-American-British classification. Numbers refer to FAB categoy M0…M7, Some patients that had between 20 and 30% blasts that were refractory anemia with excess blasts (RAEBT) under the older FAB schema, but that are now considered to be AML were included. Status: N = new, R= relapsed. Age is in years. Sex: M(Black box) = male, F= female. Race: W = caucasian, B= Black, H= Hispanic, A= Asian,. Cytogenetics (F)avorable, (I)ntermediate, (U)nfavorable. FLT3 ITD and D835: Black box = positive, N= Negative, ND = not done. Some samples were excluded from the individual protein or the network analysis as they were duplicates from the same date or same source as noted in the “Exclude” column.

	Subset Available														FLT3		
ID	Bulk	CD34+	CD34-	CD38+	CD38-	CD34+ Yield	CD34+CD38- Yield	FAB	Status	Age	SEX	RACE	Cytogenetics	Cyto Category	ITD	D835	Exclude?
2						73.5%		M2	R	72	M	W	IM	I	N	N	
22						30.8%		RAEBT	R	60	F	W	ND	ND	N	N	
23						10.8%		RAEBT	N	69	M	W	-5,-7	U	N	N	
3						89.5%		M1	R	69	F	B	ND	ND	N	N	
7						1.6%		M1	N	74	F	W	INV(9)	I	N		
9						1.3%		M2	N	59	F	A	+8	U	N	N	
11						4.3%		M2	R	28	F	W	-5,-7	U	N		
20						2.0%		M2	N	23	M	W	MISC	U	N	N	
24						3.1%		M5	N	46	M	H	DIP	I	N	N	
29						41.3%		M0	R	26	M	W	MISC	U	N	N	
35						4.5%		M5	R	62	M	H	MISC	U	N	N	
44						31.3%		M2	R	56	F	W	DIP	I	N	N	
49						5.4%		M2	N	58	F	W	DIP	I		N	
50						3.1%		M4	N	73	M	W	DIP	I	N	N	
51						2.2%		M1	N	71	F	B	DIP	I			
52						1.3%		M4	N	65	M	W	DIP	I	N	N	
56						9.1%		M4	R	55	F	B	MISC	U		N	
61						16.7%		RAEBT	N	83	M	W	+8	U	N	N	
66						2.4%		M2	N	34	F	W	DIP	I	N	N	
73						21.1%		M1	R	66	M	W	ND	ND	N	N	
104						22.6%		UNK	R	39	F	W	MISC	U	N	N	
12						38.5%		M2	R	72	F	W	DIP	I	N	N	
15						91.5%		M0	R	80	M	W	MISC	U	N	N	
19						35.3%		M2	R	55	F	B	MISC	U			
27						50.3%		M2	R	56	M	W	5Q-	U	N	N	
33						16.7%		M4	N	79	M	W	MISC	U		N	
34						34.6%		M4	N	14	M	H	INV(16)	F	ND	ND	
36						3.0%		M4	R	50	M	W	DIP	I	N	N	
37						74.3%		UNK	R	51	F	W	MISC	U			
38						45.9%		UNK	R	59	M	W	MISC	U	N	N	
40						9.5%		M4	N	49	F	H	INV(16)	F	N	N	
42						4.3%		M5	R	31	F	W	MISC	U		N	
46						18.6%		UNK	R	66	F	W	5Q-	U	N	N	

8	77.3%	7.02%	M1	N	83	M	W	MISC	U	N	N	
10	11.4%	0.57%	UNK	N	80	M	H	ND	ND	N	N	
14	8.8%	2.20%	M1	R	47	F	W	IM	I		N	
16	68.2%	3.10%	M1	R	37	F	A	MISC	U	N	N	
17	20.6%	0.98%	UNK	R	68	F	W	5Q-	U	N	N	
47	46.8%	14.05%	M5	R	32	F	B	MISC	U	N	N	
55	1.3%	0.21%	M4	N	79	F	B	DIP	I		N	
57	10.3%	0.22%	M2	N	64	M	W	DIP	I	N	N	
58	3.0%	0.68%	M2	R	19	M	H	DIP	I		N	
59	49.5%	6.19%	M2	R	50	F	W	T(8;21)	F	ND	ND	
60	57.6%	12.89%	UNK	R	78	F	A	MISC	U	N	N	
62	36.4%	6.06%	M6	R	59	M	W	-5,-7	U	N	N	
63	31.7%	0.86%	M1	N	68	M	W	DIP	I		N	
65	80.3%	2.51%	M0	N	66	M	H	MISC	U	N	N	
68	6.6%	0.78%	UNK	R	52	F	W	DIP	I		N	
72	24.2%	1.86%	M4	R	25	M	W	DIP	I	N	N	
75	37.1%	6.44%	M5	N	43	M	W	5Q-	U		N	
77	12.2%	0.56%	M0	N	78	M	W	+8	U	N	N	
78	20.2%	0.52%		N	84	M	W	DIP	I		N	
81	14.3%	0.24%	M1	N	24	F	W	DIP	I		N	
84	12.3%	1.75%	UNK	R	55	F	W	INV(16)	F	N	N	
85	22.8%	0.65%	M5	R	71	F	W	DIP	I		N	
86	48.3%	2.84%	M2	R	75	M	W	ND	ND	N	N	
87	6.8%	0.57%	UNK	R	71	F	B	MISC	U	N	N	
88	4.2%	0.28%	M2	R	83	F	W	DIP	I	N	N	
89	3.9%	0.56%	M5	R	38	F	W	DIP	I	N	N	
90	43.2%	0.94%	M4	R	26	F	W	MISC	U	N	N	
92	20.0%	2.00%	RAEBT	R	69	M	W	ND	ND	N	N	
94	70.0%	1.06%	UNK	R	64	M	W	7Q-	U	N	N	
96	88.9%	1.03%	M1	N	62	M	W	T(8;21)	F	N	N	
99	40.0%	0.87%	M4	R	67	M	W	+8	U	N	N	
101	32.8%	1.64%	RAEBT	N	18	F	W	DIP	I	N	N	
105	42.0%	1.85%	M4	R	69	F	W	DIP	I	N	N	
107	68.1%	5.24%	UNK	N	81	M	W	-5,-7	U	ND	ND	
108	55.1%	2.75%	M1	R	37	M	B	MISC	U	N	N	
53	2.5%		M5	R	31	F	W	ND	ND		N	Dates
70	8.3%		M5	R	38	F	W	ND	ND	N	N	Dates
71	10.4%		M4	R	50	M	W	ND	ND	N	N	Dates
82	8.3%		RAEBT	R	83	M	W	+8	U	N	N	Dates
28	69.1%		M2	R	55	F	B	MISC	U	N		Dates
39	55.7%		M4	R	26	F	W	ND	ND	N	N	Dates
43	50.4%		UNK	R	78	F	A	MISC	U	N	N	Dates
76	48.3%	2.91%	UNK	R	78	F	A	MISC	U	N	N	Dates
93	33.7%	4.22%	RAEBT	R	83	M	W	ND	ND	N	N	Dates

103						30.3%	1.52%	M1	N	68	M	W	ND	ND		N	Dates
109						87.2%	28.31%	M2	R	50	F	W	T(8;21)	F	ND	ND	Dates
21						1.8%		M2	N	23	M	W	MISC	U	N	N	Source
25						4.5%		M5	N	46	M	H	DIP	I	N	N	Source
48						1.6%		M2	N	58	F	W	DIP	I		N	Source
67						3.6%		M2	N	34	F	W	DIP	I	N	N	Source
79						6.7%		Bipheno	N	84	M	W	DIP	I		N	Source
41						9.1%	0.24%	M4	N	49	F	H	INV(16)	F	N	N	Source
18						16.2%	0.24%	UNK	R	68	F	W	5Q-	U	N	N	Source
64						38.6%	1.93%	M1	N	68	M	W	DIP	I		N	Source
74						30.0%	0.25%	M5	N	43	M	W	5Q-	U		N	Source
80						90.9%	0.42%	M1	N	24	F	W	DIP	I		N	Source
83						9.8%	1.09%	UNK	R	55	F	W	INV(16)	F	N	N	Source
100						34.1%	0.09%	M4	R	67	M	W	+8	U	N	N	Source
106						53.2%	3.80%	M4	R	69	F	W	DIP	I	N	N	Source

Supplemental Table 2. Antibodies used in this study. The antibodies used in this study along with the manufacturer, catalog number, primary antibody dilution, secondary antibody dilution are listed.

RPPA Antibody	Antibody Name	Hugo name	modify (hugo)	Host	company	catalog#	lot#	Storage	1st Ab dilution	2nd Ab dilution
Actin	Actin(β)	ACTB	ACTB	mouse	Sigma	A5441	055K4854,1 07K4800	-20	10000	15000-20000
AIF	AIF	AIFM1	AIFM1	mouse	santa cruz	sc-13116	C1306	4	250	15000
AKT	AKT	3	AKT1/2/3	Rabbit	cell signaling	9272	12,17	-20	150	15000
AKTpT308	AKT-P308(Thr)	3-phospho	pT308	Rabbit	cell signaling	9275	10,11	-20	150	15000
AKTp473	AKT-P473(Ser)	3-phospho	pS473	Rabbit	cell signaling	9271	9,11	-20	150	15000
AMPKa	AMPKα	2	PRKAA1/2	Rabbit	cell signaling	2532	4	-20	200	15000
AMPKap172	αP(Thr172)	2-phospho	p172	Rabbit	cell signaling	2535	3	-20	500	15000
ARC	ARC	ARC	ARC	Rabbit	Imgenex	IMG-170		4	2000	15000
BAD	bad	BAD	BAD	Rabbit	cell signaling	9292	6,9	-20	100	15000
BADp112	bad-p112(Ser)	BAD-phospho Ser112	BAD p112	Rabbit	cell signaling	9291	6,8	-20	100	15000
BADp136	bad-p136(Ser)	BAD-phospho Ser136	BAD p136	Rabbit	cell signaling	9295	7,8	-20	50	15000
BADp155	bad-p155(Ser)	BAD-phospho Ser155	BAD p155	Rabbit	cell signaling	9297	2,3	-20	100	15000
BAK	bak	BAK1	BAK1	Rabbit	cell signaling	3792	1	-20	50	15000
BAX	bax	BAX	BAX	Rabbit	cell signaling	2772	3	-20	100	15000
BCL2	bcl2	BCL2	BCL2	mouse	DAKO	M0887	6140	4	200	15000
BCLXL	bcl-XL	BCL2L1	BCL2L1	Rabbit	cell signaling	2762	3	-20	200	15000
BID	bid	BID	BID	Rabbit	cell signaling	2002	3	-20	250	15000
BIM	bim	BCL2L11	BCL2L11	Rabbit	Epitomics	1036-1	22308	-20	500-Xing	15000
C.cbl	Cbl-c	CBL	CBL	mouse	Transduction	610441	43775(2)	4	1000(2)	15000
Caspase3	caspase 3	CASP3	CASP3	Rabbit	cell signaling	9662	9	-20	1000	15000
Caspase7	*cleaved*	Asp198	CASP7 cl198	Rabbit	cell signaling	9491	6	-20	250	15000
caspase8	caspase 8	CASP8	CASP8	mouse	cell signaling	9746	7	-20	1000	15000
0	cleaved	Asp330	CASP9 cl330	Rabbit	cell signaling	9501	4	-20	150	15000
a	Catenin-alpha	CTNNA1	CTNNA1	mouse	CalBiochem	CA1030	D00003169	-20	75	15000
CateninB	Catenin-beta	CTNNB1	CTNNB1	Rabbit	cell signaling	9562	3	-20	100	15000
CD34	CD34	CD34	CD34	Rabbit	Epitomics	2150-1	E070202	-20	500	15000

CDC2	cdc2	CDK1	CDK1	mouse	calbiochem	cc01	D30015	4	200	15000
CDK2	cdk2	CDK2	CDK2	mouse	santa Cruz	sc6248	D1607	4	200	15000
CDK4	cdk4	CDK4	CDK4	mouse	cell signaling	2906	2	-20	250-X	15000
CIAP	CIAP-1	BIRC2	BIRC2	Rabbit	upstate	07-759	30622	-20	200-400	15000
CyclinB1	cyclin B1	CCNB1	CCNB1	Rabbit	Epitomics	1495-1	yc053103	-20	500	15000
CyclinD1	20)	CCND1	CCND1	Rabbit	santa Cruz	sc-718	H2007	4	1000-D	15000
CyclinD3	cyclin D3	CCND3	CCND3	mouse	Cell Signaling	2936	2	-20	100	15000
CyclinE	cyclin E	CCNE1	CCNE1	mouse	santa Cruz	sc-247	E2406	4	500-X	15000
ERK2	erk2	MAPK1	MAPK1	Rabbit	santa Cruz	Sc-154	A0605	4	10000	15000
ERK2p	p42/44(Thr202/	phospho	p202/204	Rabbit	cell signaling	9101	16	-20	1000	15000
FAK	FAK	PTK2	PTK2	Rabbit	Cell Signaling	3285	2	-20	500	15000
GAB2	Gab2	GAB2	GAB2	Rabbit	cell signaling	3239	1	-20	500	15000
GAB2p	pGab2-Tyr452	Tyr452	GAB2-p452	Rabbit	cell signaling	3882	1	-20	25	15000
GAPDH	GAPDH	GAPDH	GAPDH	mouse	Ambion	AM4300	86081	-20	2000	15000
GSK3	GSK3	GSKA/GSKB	GSKA/B	mouse	santa Cruz	sc-7291	L1605	4	200	15000
GSK3p	ser21/9)	phospho Ser21/9	GSKA/B-p21/9	Rabbit	cell signaling	9331	2a	-20	200	15000
HDAC3	HDAC3	HDAC3	HDAC3	Rabbit	cell signaling	2632	3	-20	100	15000
HIF1a	HIF-1α	HIF1A	HIF1A	Mouse	BD pharMingen	610959	54033	-20	25	15000
HSP70	HSP70	HSPA1A/HSPA1 L	HSPA1A/L	Rabbit	Cell Signaling	4872	2	-20	250	15000
HSP90	HSP90	HSP90AA1/HSP 90AB1	HSP90AA1/B1	Rabbit	Cell Signaling	4874	2	-20	500	15000
IntegrinB3	integrin-beta3	ITGB3BP	ITGB3BP	Rabbit	cell signaling	4702	1	-20	250	15000
JAB1	JAB1	COPS5	COPS5	mouse	santa Cruz	sc-13157	10605	4	**300**-500	15000
JAZ111	JAZ111	ZNF346	ZNF346	Rabbit	May/yang(UFL)			-20	1000	20000
JUNB	Jun-B	JUNB	JUNB	Rabbit	cell signaling	3755	1	-20	100	15000
LCK	Lck	LCK	LCK	Rabbit	cell signaling	2752	2	-20	500	15000
LKB1	LKB1/STK11	STK11	STK11	Rabbit	cell signaling	3050	1	-20	500	15000
LYN	lyn	LYN	LYN	Rabbit	cell signaling	2732	2	-20	250	15000
MCL1	MCL1	MCL1	MCL1	mouse	pharmingen	559027	83737	4	50	15000
MDM2	MDM2	MDM2	MDM2	Rabbit	Santa Cruz	sc-813	H3007	4	5000	15000
MEK	MEK	2	MAP2K1/2	Rabbit	cell signaling	9122	6	-20	5000	15000
MEKp	ser217/221)	2-phospho	p217/221	Rabbit	cell signaling	9121	6	-20	2000	15000

MTOR	mTor	MTOR	MTOR	Rabbit	cell signaling	2983	3	-20	200	15000
MTORp	Ser2448	Ser2448	MTOR p2448	Rabbit	cell signaling	2971	4,12	-20	150-B	15000
MYC	myc(c-)	MYC	MYC	Rabbit	cell signaling	9402	2	-20	100	15000
NFKB.P65	NF-kB p65	RELA	RELA	Rabbit	cell signaling	3034	2	-20	500	15000
p.EIF2.alpha	alpha	Ser51	EIF2S1 p51	Rabbit	cell signaling	9721	2	-20	150	15000
p.FoxO1.3A	FoxO1a/3a	FOXO1-phospho thr24/FOXO3-	FOXO1p24/FOXO3p32	Rabbit	Cell Signaling	9464		-21	100(B)	15000
p.IRS.1	p-IRS-1 (S1101)	IRS1-phospho ser 1101	IRS1-phospho ser 1101	Rabbit	Cell Signaling	2385	1	-20	250	15000
P21	p21	CDKN1A	CDKN1A	mouse	Cell signaling	2946	3	-20	250	15000
P27	p27	CDKN1B	CDKN1B	Rabbit	santa Cruz	sc-528	D0607	4	250	15000
P38	p38	MAPK14	MAPK14	Rabbit	cell signaling	9212	11	-20	700	15000
P38p	thr180/tyr182	phospho	p180/182	Rabbit	cell signaling	9211	17	-20	100	15000
P53	p53	TP53	TP53	mouse	BD Biosciences	554294	52418	4	750	15000
P53pser15	p53-p-ser15	Ser15	TP53 p15	Rabbit	cell signaling	9284	8	-20	250	15000
P70S6K	p70S6K	RPS6KB1	RPS6KB1	Rabbit	Cell Signaling	9202	7	-20	250	15000
P70S6Kp	p70S6K(p-thr389)	RPS6KB1-phospho thr389	RPS6KB1 ph389	Rabbit	cell signaling	9205	5	-20	250	15000
PARP	PARP	PARP1	PARP1	Rabbit	cell signaling	9542	5	-20	200	15000
PDK1	PDK1	PDK1	PDK1	Rabbit	cell signaling	3062	3	-20	200	15000
PDK1p	p241(Ser)	ser241	PDK1 p241	Rabbit	cell signaling	3061	5	-20	500	15000
PI3K110	alpha	PIK3CA	PIK3CA	Rabbit	Epitomics	1683-1	D031312	-20	500-X	15000
PI3K85	PI3 K p85	PIK3R1/PIK3R2	PIK3R1/2	Rabbit	cell signaling	4292	4	-20	100	15000
64	p664(Ser)	Ser664	PRKCD p664	Rabbit	Upstate	07-875	JBC138623	-20	500	15000
PKCa	PKCα	PRKCA	PRKCA	mouse	Upstate	05-154	23336	-20	5000	15000
PKCaphos	p657(Ser)	ser657	PRKCA-p657	Rabbit	Upstate	06-822	21320	-20	5000	15000
pMet.C	Met(Py1230/1234/1235	Py1230/1234/1235	p1230/1234/1235	Rabbit	Biosource	44-888G		-20	250	15000
PP2A	PP2A-B55	2A/PPP2R2B/PP	δ	goat	santa Cruz	sc-18330	D1404	4	500	15000
a	PPARγ	PPARG	PPARG	mouse	santa Cruz	sc7273	F0106	4	75	15000
PRKR	PKR(EIF2AK2)	EIF2AK2	EIF2AK2	mouse	Abnova	M02	00Aa6	-20	5000	15000
PTEN	PTEN	PTEN	PTEN	Rabbit	cell signaling	9552	2	-20	500-X	15000
PTENp	PTEN-p380	PTEN-phospho	PTEN-phospho	Rabbit	cell signaling	9551	4	-20	500	15000

PU1	PU.1	SPI1	SPI1	Rabbit	cell signaling	2258		-20	5000	15000
Rac1.2.3	Rac1/2/3	RAC1/RAC2/RA C3	RAC1/2/3	Rabbit	Cell Signaling	2465	1	-20	500	15000
RAFB	Raf-B	BRAF	BRAF	mouse	Santa cruz	sc5284	E1006	4	100	15000
RB	Rb	RB1	RB1	mouse	PharMingen	554136	52561	4	100	15000
RBp	pRb (P-Ser807/811)	RB1-phospho ser807/811	RB1 p807/811	Rabbit	Cell Signaling	9308	9	-20	750	15000
S6RP	protein	RPS6	RPS6	Rabbit	cell signaling	2217	1	-20	200-X	15000
S6Rpp235	protein(phosph	ser235/236	p235/236	Rabbit	cell signaling	2211	11	-20	30000-X	15000
S6Rpp240	protein(phosph	ser240/244	p240/244	Rabbit	cell signaling	2215	4	-20	3000-X	15000
SHIP1	SHIP1	INPP5D	INPP5D	mouse	Santa Cruz	sc-8425	E2406	4	**100**	15000
SHIP2	SHIP2	INPPL1	INPPL1	Rabbit	cell signaling	2730	1	-20	100-300	15000
SMAC	Smac/Diablo	DIABLO	DIABLO	mouse	cell signaling	2954	1	-20	3000	15000
SMAD1	smad1	SMAD1	SMAD1	Rabbit	Epitomics	1649-1	YD020503	-20	200	15000
SMAD4	smad4	SMAD4	SMAD4	mouse	Santa Cruz	sc-7966	G0907	4	200	15000
SRC	Src	SRC	SRC	mouse	Upstate	05-184	25373=2		200-D	15000
SRCp416	tyr416)	tyr416	SRC p416	Rabbit	cell signaling	2101	5	-20	250-X	15000
SRCp527	tyr527)	tyr527	SRC p527	Rabbit	cell signaling	2105	5	-20	400	15000
SSBP2.alpha	SSBP2-I(alpha)	SSBP2	SSBP2	Rabbit	Dr. Nagarajan			4	1000	15000
STAT1	stat1	STAT1	STAT1	Rabbit	cell signaling	9172	9	-20	250	15000
STAT1p	tyr701)	tyr701	STAT1 p701	Rabbit	cell signaling	9171	5,6	-20	100	15000
STAT3	stat3	STAT3	STAT3	Rabbit	Upstate	06-596	29693	-20	100	15000
STAT3p705	stat3 p705(Tyr)	tyr705	STAT3 p705	Rabbit	cell signaling	9131	2,5	-20	400-D	15000
STAT3p727	p727(Ser)	ser727	STAT3 p727	Rabbit	cell signaling	9134	5	-20	200	15000
STAT5	stat5	B	STAT5A/B	Rabbit	cell signaling	9352	2	-20	250	15000
STAT5.p694	Tyr694)	B phospho	p694	Rabbit	cell signaling	9351	4	-20	50	15000
STAT6p641	tyr641)	tyr641	STAT6 p641	Rabbit	cell signaling	9361	3,9	-20	300	15000
Survivin	survivin	BIRC5	BIRC5	mouse	cell signaling	2802	2	-20	50	15000
TCF4	TCF-4	TCF4	TCF4	Goat	Santa cruz	sc-8632	H1607	4	400	15000
TNK1	TNK1	TNK1	TNK1	Rabbit	abgent	ap7722	H	-20	400	15000
TRIM24	TRIM24	TRIM24	TRIM24	Rabbit	Novus	NB100-2597		4	2000	15000

TRIM62	TRIM62	TRIM62	TRIM62	mouse	Abnova	H00055223-B01	8032	-20	1000	15000
TSC2	TSC2	TSC2	TSC2	Rabbit	Epitomics	1613-1	YC120610=Lot 1; YD011907=Lot 2	-20	500	15000
XIAP	XIAP	XIAP	XIAP	Rabbit	cell signaling	2042	4,6	-20	200	15000
YAP	YAP	YAP1	YAP1	Rabbit	cell signaling	4912	1	-20	100	15000
YAP.p	pYAP-ser127	Ser127	YAP1 p127	Rabbit	cell signaling	4911	1	-20	500	15000
ZNF342	ZNF342	ZNF296	ZNF296	Rabbit	Abcam	ab51265		-20	2000	15000

Blue: antibodies aren't commercially available 5

Supplemental Table 3. Rosetta stone of protein names based on HUGO, Mimi and antibody names. The different databases used in this manuscript frequently have different names for the same protein. This table lists the protein names used in the RPPA analysis, the manufacuters name for the antibody, the Hugo name, our modification of the Hugo name to account for phosphorylation and cleavage sites and finaly the MIMI name. Since HUGO names do not include any post-tranaslational modification data there is not a different name for a protein that is phosphorylated on a given site. The common practice of using a lower case "p" before the protein name prevents alphabetical sorting. With that convention all the phospho proteins sort together and no way to sort the total and phosphorylated form of protein so they come out next to each other in a list. We use a convention where the protein name comes first, followed by a dot, which is then followed by any post-translational modifications, (e.g. a p for phosphorylation, cl for cleaved) and then the amino acid number that is affected. (e.g. AKT.pThr308). Names that are identical across all of these have a green background.. Names where Hugo and MIMI differ have a red background. This table includes all antibodies that we have validated for use in RPPA, however only 121 were used in this analysis.

Protein Name RPPA	Antibody Name	Hugo name	Hugo (modified)	MIMI Name
	Abl-c	ABL1	ABL1	ABL1
	ACC (P-S79)	ACACA/ACACB	ACACAp79 /B	ACACA
ACTIN	Actin(β)	ACTB	ACTB	ACTB
	AIB1	NCOA3	NCOA3	NCOA3
AIF	AIF	AIFM1	AIFM1	AIFM1
AKT	AKT1	AKT1	AKT1	AKT1
	AKT2	AKT2	AKT2	AKT2
	AKT3	AKT3	AKT3	AKT3
AKTp308	AKT-P308(Thr)	AKT1/AKT2/AKT3-phospho Thr308	AKT1/2/3 p308	AKT1
AKTp473	AKT-P473(Ser)	AKT1/AKT2/AKT3-phospho ser473	AKT1/2/3 p473	AKT1
AMPKa.phos	AMPK αP(Thr172)	PRKAA1/PRKAA2-phospho Thr172	PRKAA1/2 p172	PRKAA1
AMPKa	AMPKα	PRKAA1/PRKAA2	PRKAA1/2	PRKAA1
	Androgen Receptor	AR	AR	AR
ARC	ARC	ARC	ARC	ARC
Ash2L	Ash2L	ASH2L	ASH2L	ASH2L
ASNS	ASNS	ASNS	ASNS	ASNS
ATF3	ATF3	ATF3	ATF3	ATF3
ATG7	ATG7	ATG7	ATG7	ATG7
BAD	bad	BAD	BAD	BAD
BADp112	bad-p112(Ser)	BAD-phospho Ser112	BAD p112	BAD
BADp136	bad-p136(Ser)	BAD-phospho Ser136	BAD p136	BAD
BADp155	bad-p155(Ser)	BAD-phospho Ser155	BAD p155	BAD
BAK	bak	BAK1	BAK1	BAK1
BAX	bax	BAX	BAX	BAX
BCL2	bcl2	BCL2	BCL2	BCL2
BCLXL	bcl-XL	BCL2L1	BCL2L1	BCL2L1
Beclin.1	Beclin-1	BECN1	BECN1	BECN1
BID	bid	BID	BID	BID
BIM	bim	BCL2L11	BCL2L11	BCL2L11
GRP78	bip/GRP78	GRP78	GRP78	HSPA5
Bmi.1	Bmi-1	BMI1	BMI1	BMI1

C23.nucleolin	C23 (nucleolin)	NCL	NCL	NCL
	Cadherin-p	CDH3	CDH3p	CDH3
Caspase3	caspase 3	CASP3	CASP3	CASP3
CASP3clvd	caspase 3 cleaved Asp175	CASP3 cleaved	CASP3 cl175	CASP3
CASP7Clvd	*caspase 7 cleaved Asp198*	CASP7 cleaved Asp198	CASP7 cl198	CASP7
CASPASE8	caspase 8	CASP8	CASP8	CASP8
CASP9	*caspase 9*	CASP9	CASP9	CASP9
CASP9.Asp315	caspase 9 cleaved Asp315	CASP9 cleaved Asp315	CASP9 cl315	CASP9
CASP9.Asp330	caspase 9 cleaved Asp330	CASP9 cleaved Asp330	CASP9 cl330	CASP9
CateninA	Catenin-alpha	CTNNA1	CTNNA1	CTNNA1
CateninB	Catenin-beta	CTNNB1	CTNNB1	CTNNB1
CateninBp	catenin-beta phospho-	CTNNB1-phospho Ser33/37/Thr41	CTNNB1 p33/37/41	CTNNB1
Cavelin.1	Caveolin-1	CAV1	CAV1	CAV1
C.cbl	Cbl-c	CBL	CBL	CBL
CD11A	CD11a	ITGAL	ITGAL	ITGAL
CD20	CD20	MS4A1	MS4A1	MS4A1
	CD31/PECAM	PECAM1	PECAM1	PECAM1
CD34	CD34	CD34	CD34	CD34
CD44.Epi	CD44.Epi	CD44	CD44	CD44
CD49B	CD49b	ITGA2	ITGA2	ITGA2
CD74	CD74	CD74	CD74	CD74
CDC2	cdc2	CDK1	CDK1	CDC2
CDK2	cdk2	CDK2	CDK2	CDK2
CDK4	cdk4	CDK4	CDK4	CDK4
CIAP	CIAP-1	BIRC2	BIRC2	BIRC2
CaseinKinase2al	CK2α	CSNK2A1	CSNK2A1	CSNK2A1
Kit.C	c-Kit	KIT	KIT	KIT
	Collagen type VI	COL6A1	COL6A1	COL6A1
Cox2	cox-2	PTGS2	PTGS2	PTGS2
CREB	CREB	CREB1	CREB1	CREB1
CREBps133	CREB-p(ser133)	Ser133	CREB1 p133	CREB1
CRM1.XPO1	CRM1	XPO1	XPO1	XPO1
CyclinB1	cyclin B1	CCNB1	CCNB1	CCNB1
CyclinD1	cyclin D1(M-20)	CCND1	CCND1	CCND1
CyclinD3	cyclin D3	CCND3	CCND3	CCND3
CyclinE	cyclin E	CCNE1	CCNE1	CCNE1

CyclinE2	cyclin E2	CCNE2	CCNE2	CCNE2
	DAP5	EIF4G2	EIF4G2	EIF4G2
DJ1	DJ-1	PARK7	PARK7	PARK7
DLX1	DLX1	DLX1	DLX1	DLX1
EBP1	EBP1	PA2G4	PA2G4	PA2G4
EBP1.pser65	EBP1.pser65	PA2G4	PA2G4.pS65	PA2G4
EBP1.pthr37.46	EBP1.pthr37.46	PA2G4	PA2G4.pT37.p46	PA2G4
EBP1.pthr70	EBP1.pthr70	PA2G4	PA2G4.pT70	PA2G4
EGFR	EGFR	EGFR	EGFR	EGFR
EGFRp992	EGFR-p tyr992	EGFR-phospho Tyr992	EGFR-p992	EGFR
Egln1	Egln1	EGLN1	EGLN1	EGLN1
EIF4E	eIF4E	EIF4E	EIF4E	EIF4E
EIF2	elF2	EIF2S1	EIF2S1	EIF2S1
ElKp383	ElK(phospho-ser383)	ELK1-phospho Ser383	ELK1 p383	ELK1
EGR123	ERG1/2/3	ERG	ERG	EGR1
ERK2	erk2	MAPK1	MAPK1	MAPK1
ERK2p42.44	erk-p42/44(Thr202/Tyr204	MAPK1/MAPK3-phospho Thr202/Tyr204	MAPK1/3 p202/204	MAPK1
FAK	FAK	PTK2	PTK2	PTK2
Fibronectin	Fibronectin	FN1	FN1	FN1
FOXO3Ap	FKHRL1/FoxO3a (P-Ser 318/321)	FOXO3-phospho Ser318/321	FOXO3-p318/321	FOXO3
Fli	Fli	Fli1	Fli1	FLI1
FoxO1.3Ap	FoxO1a/3a	FOXO1-phospho thr24/FOXO3-phospho thr32	FOXO1p24/FOXO3p32	FOXO1
FOXO3A	FoxO3a	FOXO3	FOXO3	FOXO3
GAB2	Gab2	GAB2	GAB2	GAB2
GAB2phos	Gab2-pTyr452	GAB2-phospho Tyr452	GAB2-p452	GAB2
Galetin.3	galectin-3	LGALS3	LGALS3	LGALS3
GAPDH	GAPDH	GAPDH	GAPDH	GAPDH
GATA.1	GATA-1	GATA1	GATA1	GATA1
Gata3	Gata3	GATA3	GATA3	GATA3
GSK3	GSK3	GSKA/GSKB	GSKA/B	GSK3A
GSK3p21.9	GSK3a/B(p-ser21/9)	GSKA/GSKB-phospho Ser21/9	GSKA/B-p21/9	GSK3A
hnRNPK	h nRNP k	HNRNPK	HNRNPK	HNRPK
HDAC1	HDAC1	HDAC1	HDAC1	HDAC1

HDAC2	HDAC2	HDAC2	HDAC2	HDAC2
HDAC3	HDAC3	HDAC3	HDAC3	HDAC3
HER2p	HER2(p-Tyr1248)	ERBB2-phospho Tyr1248	ERBB2 p1248	ERBB2
HER2	HER2/Erb2	ERBB2	ERBB2	ERBB2
HER3	HER3	ERBB3	ERBB3	ERBB3
HIF1a	HIF-1α	HIF1A	HIF1A	HIF1A
HSP27	HSP27	HSPB1	HSPB1	HSPB1
HSP70	HSP70	HSPA1A/HSPA1L	HSPA1A/L	HSPA1A
HSP90	HSP90	HSP90AA1/HSP90AB1	HSP90AA1/B1	HSP90AA1
IGFBP2	IGFBP-2	IGFBP2	IGFBP2	IGFBP2
IGF.1	IGF-I Rβ	IGF1R	IGF1R	IGF1R
IntegrinB3	integrin-beta3	ITGB3	ITGB3	ITGB3
IRS.1p	p-IRS-1 (S1101)	IRS1-phospho ser 1101	IRS1-phospho ser 1101	IRS1
JAB1	JAB1	COPS5	COPS5	COPS5
JAZ	JAZ111	ZNF346	ZNF346	ZNF346
JMJD6	JMJD6	JMJD6	JMJD6	JMJD6
	JNK1	MAPK8	MAPK8	MAPK8
JNK2	JNK2	MAPK9	MAPK9	MAPK9
JUNB	Jun-B	JUNB	JUNB	JUNB
Jun.C.pser73	Jun-c(p-ser73	JUN-phospho Ser73	JUN-p73	JUN
LCK	Lck	LCK	LCK	LCK
LEF1	LEF1	LEF1	LEF1	LEF1
LKB1	LKB1/STK11	STK11	STK11	STK11
LYN	LYN	LYN	LYN	LYN
MCL1	MCL1	MCL1	MCL1	MCL1
MDM2	MDM2	MDM2	MDM2	MDM2
MDM4	MDM4	MDM4	MDM4	MDM4
MEK	MEK	MAP2K1	MAP2K1	MAP2K1
MEKp217p221	MEK(p-ser217/221)	MAP2K1/MAP2K2-phospho ser217/221	MAP2K1/2 p217/221	MAP2K1
MSI2	MSI2	MSI2	MSI2	MSI2
MTOR	mTor	MTOR	MTOR	FRAP1
MTORp2448	mTor(p-Ser2448)	MTOR-phospho Ser2448	MTOR p2448	FRAP1
MYC	Myc(c-)	MYC	MYC	MYC
NF2	NF2	NF2	NF2	NF2
NFKB.P65	NF-kB p65	RELA	RELA	NFKB1

Notch1clvd	Notch1-cleaved (Val1744)	NOTCH1 cleaved val1744	NOTCH1 cl1744	NOTCH1
Notch3	Notch3	NOTCH3	NOTCH3	NOTCH3
NPM	NPM	NPM1	NPM1	NPM1
NPM3542	NPM3542	NPM1	NPM1	NPM1
NRP1	NRP1(neuropilin)	NRP1	NRP1	NRP1
NURR77	Nur77	NR4A1	NR4A1	NR4A1
ODC	ODC	ODC1	ODC1	ODC1
OPN	Osteopotin	SPP1	SPP1	SPP1
P16	P16	CDKN2A	CDKN2A	CDKN2A
P21	P21/Waf	CDKN1A	CDKN1A	CDKN1A
P27	P27	PSMD9	PSMD9	PSMD9
P27ps10	P27ps10	PSMD9	PSMD9	PSMD9
	P300	EP300	EP300	EP300
P38	P38	MAPK14	MAPK14	MAPK14
P38p180p182	P38p180p182	MAPK14	MAPK14	MAPK14
P53	P53	TP53	TP53	TP53
P53pSER15	P53pSER15	TP53	TP53	TP53
P62	P62	SQSTM1	SQSTM0	SQSTM1
P70S6K	p70S6K	RPS6KB1	RPS6KB1	RPS6KB1
P70S6Kp	p70S6K(p-thr389)	RPS6KB1-phospho thr389	RPS6KB1 ph389	RPS6KB1
PARP	PARP	PARP1	PARP1	PARP1
PARPclvd	PARP(cleaved Asp214)	PARP1-cleaved	PARP1 cl214	PARP1
PDK1	PDK1	PDK1	PDK1	PDK1
PDK1p	PDK1-p241(Ser)	PDK1-phospho ser241	PDK1 p241	PDK1
EIF2A.phos	phospho-eIF2-alpha	EIF2S1-phospho Ser51	EIF2S1 p51	EIF2S1
CMETp	Met(Py1230/1234/1235	MET-phospho Py1230/1234/1235	MET p1230/1234/1235	MET
PI3Kp110	alpha	PIK3CA	PIK3CA	PIK3CA
PI3Kp85	PI3 K p85	PIK3R1/PIK3R2	PIK3R1/2	PIK3R1
PIM.1	PIM1	PIM1	PIM1	PIM1
PIM.2	PIM2	PIM2	PIM2	PIM2
PKCalpha	PKCα	PRKCA	PRKCA	PRKCA
PKCap657	PKCα-p657(Ser)	PRKCA-phospho ser657	PRKCA-p657	PRKCA
PKCB.I	PKCβ I	PRKCB	PRKCB	PRKCB
PKCBII	PKCβII	PRKCB	PRKCB	PRKCB

PKCDelta.p507	PKCδ-507(Thr)	PRKCD-phospho Thr507	PRKCD p507	PRKCD
PKCdeltap664	PKCδ-645(Ser)	PRKCD-phospho Ser645	PRKCD p645	PRKCD
PKCg645	PKCδ-p664(Ser)	PRKCD-phospho Ser664	PRKCD p664	PRKCG
PLAC1	PLAC1	PLAC1	PLAC1	PLAC1
NF2p	pNF2(ser518)	NF2-phospho ser518	NF2 p518	NF2
PP2A	PP2A-B55	PPP2R2D/PP2R2A/PPP2R2B/PPP2R2C	PPP2R2α/β/γ/δ	PPP2R4
PPARgam	PPARγ	PPARG	PPARG	PPARG
	PRAS40	AKT1S1	AKT1S1	AKT1S1
	PRAS40 pThr246	AKT1S1 phospho Thr246	AKT1S1 p246	AKT1S1
PRKR	K2)	EIF2AK2	EIF2AK2	EIF2AK2
PKR.pT451	(EIF2AK2)	EIF2AK2	EIF2AK2	EIF2AK2
	Receptor	PGR	PGR	PGR
PTEN	PTEN	PTEN	PTEN	PTEN
PTENp	PTENp	PTEN	PTEN	PTEN
PU1	PU.1	SPI1	SPI1	SPI1
RAC123	Rac1/2/3	RAC1/RAC2/RAC3	RAC1/2/3	RAC1
RAFB	Raf-B	BRAF	BRAF	BRAF
RB	Rb	RB1	RB1	RB1
RBp807p811	Rb (P-Ser807/811)	RB1-phospho ser807/811	RB1 p807/811	RB1
S6RP	protein	RPS6	RPS6	RPS6
S6RPp235p236	protein(phospho-	ser235/236	RPS6 p235/236	RPS6
S6RPp240p244	protein(phospho-	ser240/244	RPS6 p240/244	RPS6
SHIP	SHIP1	INPP5D	INPP5D	INPP5D
SHIP2	SHIP2	INPPL1	INPPL1	INPPL1
SHP.2	SHP-2	PTPN11	PTPN11	PTPN11
SIRT1	SIRT1	SIRT1	SIRT1	SIRT1
SMAC	Smac/Diablo	DIABLO	DIABLO	DIABLO
SMAD1	smad1	SMAD1	SMAD1	SMAD1
SMAD2	SMAD2	SMAD2	SMAD2	SMAD2
SMAD2.p245	SMAD2.p245	SMAD2	SMAD2.p245	SMAD2
SMAD2.pS465	SMAD2.pS465	SMAD2	SMAD2.pS465	SMAD2
SMAD3	SMAD3	SMAD3	SMAD3	SMAD3
SMAD4	smad4	SMAD4	SMAD4	SMAD4
SMAD5	SMAD5	SMAD5	SMAD5	SMAD5
Smad5.pS463	Smad5.pS463	SMAD5	SMAD5pS463	SMAD5

SMAD6	Smad6	SMAD6	SMAD6	SMAD6
SRC	Src	SRC	SRC	SRC
SRCp416	Src(phospho-tyr416)	SRC-phospho tyr416	SRC p416	SRC
SRCp527	Src(phospho-tyr527)	SRC-phospho tyr527	SRC p527	SRC
SSBP2	SSBP2-I(alpha)	SSBP2	SSBP2	SSBP2
STAT1	stat1	STAT1	STAT1	STAT1
STAT1p701	stat1(phospho-tyr701)	STAT1-phospho tyr701	STAT1 p701	STAT1
STAT3	stat3	STAT3	STAT3	STAT3
STAT3p705	stat3 p705(Tyr)	STAT3-phospho tyr705	STAT3 p705	STAT3
STAT3p727	stat3-p727(Ser)	STAT3-phospho ser727	STAT3 p727	STAT3
STAT5	stat5	STAT5A/STAT5B	STAT5A/B	STAT5A
STAT5p694	Stat5(phospho-Tyr694)	STAT5A/STAT5B phospho Tyr694	STAT5A/B p694	STAT5A
STAT6p641	Stat6(phospho-tyr641)	STAT6-phospho tyr641	STAT6 p641	STAT6
Stathmin	Stathmin	STMN1	STMN1	STMN1
Survivin	survivin	BIRC5	BIRC5	BIRC5
TAU	Tau	MAPT	MAPT	MAPT
TAZ	TAZ	TAZ	TAZ	TAZ
TAZ.pser89	TAZ p-Ser89	TAZ-phospho Ser89	TAZ p89	TAZ
TCF4	TCF-4	TCF4	TCF4	TCF4
TG2	TG2	TGM2	TGM2	TGM2
TNK1	TNK1	TNK1	TNK1	TNK1
TRIM24	TRIM24	TRIM24	TRIM24	TRIM24
TRIM62	TRIM62	TRIM62	TRIM62	TRIM62
TSC2	TSC2	TSC2	TSC2	TSC2
VASP	VASP	VASP	VASP	VASP
VEGFR2	VEGFR2	KDR	KDR	KDR
VHL	VHL	VHL	VHL	VHL
WTAP	WTAP	WTAP	WTAP	WTAP
X14.3.3Epsilon	X14.3.3Epsilon	YWHAE	YWHAE	YWHAE
X14.3.3Sigma	X14.3.3Sigma	SFN	SFN	SFN
X14.3.3Z	X14.3.3Z	YWHAZ	YWHAZ	YWHAZ
XIAP	XIAP	XIAP	XIAP	BIRC4
YAP	YAP	YAP1	YAP1	YAP1
YAPphos	YAPphos	YAP1	YAP1p	YAP1

ZNF342	ZNF342	ZNF296	ZNF296	ZNF296

Supplemental Table 4. Number of comparisons by sample subset.

Fraction	Total	CD34+	CD34-	Stem	Other	Stem & other
Bulk	82	77	79	29	56	28
CD34+	82		81	29	54	28
CD34-	85	81		30	55	29
Bulk & CD34+	77		76	29	53	28
Other	57	54	55	29		29
Stem	30	29	30		29	29

Supplementary Table 5. Proteins that were not different between LSC and Bulk Cells ($p > 0.01$).

Apoptosis	Cell Cycle	Expression	Proliferation	Signalling	Miscellaneous
ARC		MTORp	AMPKα	AKTp308	EIF2α-p
BCLXL		FOXO1-3Ap	AMPKα-p172	AKTp473	HSP70
Caspase3			MYC	ERK2p	Integrinβ3
Caspase9-Asp330			GSK3	FAK	MDM2
PARP			*SpI (Pu.1)*	MEKp	RAC1-2-3
XIAP			*RB*	P38p	SHIP2
			SRCp416	PI3KP85	
			TNK1	PKCα-p	
				PKCδ-p664	
				PTEN	
				PTENp	
				RAFβ	
				STAT3p705	
				STAT3p727	

Supplementary Table 6. Cytoscape figure legends (See Website Links for Higher Resolution)

Supplemental Figure 6 Legend. Cytoscape Node Color Legends

Color	Gene Pathway
	[path:hsa04115]; Apoptosis [path:hsa04210]; Natural killer cell mediated cytotoxicity [path:hsa04650]
	[path:hsa04115]; mTOR signaling pathway [path:hsa04150]; Insulin signaling pathway [path:hsa04910]
	Acute myeloid leukemia [path:hsa05221]
	Adherens junction [path:hsa04520]; Tight junction [path:hsa04530]; Leukocyte transendothelial migration [path:hsa04670]; Endometrial cancer [path:hsa05213]
	Antigen processing and presentation [path:hsa04612]
	Apoptosis [path:hsa04210]
	Apoptosis [path:hsa04210]; Focal adhesion [path:hsa04510]; Small cell lung cancer [path:hsa05222]
	B cell receptor signaling pathway [path:hsa04662]; Fc epsilon RI signaling pathway [path:hsa04664]; Long-term depression [path:hsa04730]; Epithelial cell signaling in Helicobacter pylori infection [path:hsa05120
	Cell Communication [path:hsa01430]; Focal adhesion [path:hsa04510]; Adherens junction [path:hsa04520]; Tight junction [path:hsa04530]; Pathogenic Escherichia coli infection [path:hsa05130]; [path:hsa05131]
	Cell adhesion molecules (CAMs) [path:hsa04514]; Hematopoietic cell lineage [path:hsa04640]
	Cell cycle [path:hsa04110]; [path:hsa04115]; Glioma [path:hsa05214]; Prostate cancer [path:hsa05215]; Melanoma [path:hsa05218]; Bladder cancer [path:hsa05219]; Chronic myeloid leukemia [path:hsa05220]
	Cell cycle [path:hsa04110]; [path:hsa04115]; Prostate cancer [path:hsa05215]; Small cell lung cancer [path:hsa05222]
	Cell cycle [path:hsa04110]; [path:hsa04115]; Tight junction [path:hsa04530]; T cell receptor signaling pathway [path:hsa04660]; Pancreatic cancer [path:hsa05212]; Glioma [path:hsa05214]; Melanoma [path:hsa05218]; Bladder cancer [path:hsa05219]; Chronic myeloid leukemia [path:hsa05220]; Small cell lung cancer [path:hsa05222]; Non-small cell lung cancer [path:hsa05223]
	Cell cycle [path:hsa04110]; [path:hsa04115]; Wnt signaling pathway [path:hsa04310]; Focal adhesion [path:hsa04510]; Jak-STAT signaling pathway [path:hsa04630]
	Cell cycle [path:hsa04110]; [path:hsa04115]; Wnt signaling pathway [path:hsa04310]; Focal adhesion [path:hsa04510]; Jak-STAT signaling pathway [path:hsa04630]; Colorectal cancer [path:hsa05210]; Pancreatic cancer [path:hsa05212]; Endometrial cancer [path:hsa05213]; Glioma [path:hsa05214]; Prostate cancer [path:hsa05215]; Thyroid cancer [path:hsa05216]; Melanoma [path:hsa05218]; Bladder cancer [path:hsa05219]; Chronic myeloid leukemia [path:hsa05220]; Acute myeloid leukemia [path:hsa05221]; Small cell lung cancer [path:hsa05222]; Non-small cell lung cancer [path:hsa05223]
	Cell cycle [path:hsa04110]; Pancreatic cancer [path:hsa05212]; Glioma [path:hsa05214]; Prostate cancer [path:hsa05215]; Melanoma [path:hsa05218]; Bladder cancer [path:hsa05219]; Chronic myeloid leukemia [path:hsa05220]; Small cell lung cancer [path:hsa05222]; Non-small cell lung cancer [path:hsa05223]
	Cell cycle [path:hsa04110]; Wnt signaling pathway [path:hsa04310]; TGF-beta signaling pathway [path:hsa04350]; Adherens junction [path:hsa04520]; Colorectal cancer [path:hsa05210]; Pancreatic cancer [path:hsa05212]; Chronic myeloid leukemia [path:hsa05220]
	Colorectal cancer [path:hsa05210]

	Cytokine-cytokine receptor interaction [path:hsa04060]; Axon guidance [path:hsa04360]; Focal adhesion [path:hsa04510]; Adherens junction [path:hsa04520]; Epithelial cell signaling in Helicobacter pylori infection [path:hsa05120]; Colorectal cancer [path:hsa05210]; Renal cell carcinoma [path:hsa05211]; Melanoma [path:hsa05218]
	ErbB signaling pathway [path:hsa04012]; Cell cycle [path:hsa04110]; [path:hsa04115]; Glioma [path:hsa05214]; Prostate cancer [path:hsa05215]; Melanoma [path:hsa05218]; Bladder cancer [path:hsa05219]; Chronic myeloid leukemia [path:hsa05220]
	ErbB signaling pathway [path:hsa04012]; Jak-STAT signaling pathway [path:hsa04630]; Chronic myeloid leukemia [path:hsa05220]; Acute myeloid leukemia [path:hsa05221]
	ErbB signaling pathway [path:hsa04012]; Jak-STAT signaling pathway [path:hsa04630]; T cell receptor signaling pathway [path:hsa04660]; Insulin signaling pathway [path:hsa04910]; Chronic myeloid leukemia [path:hsa05220]
	ErbB signaling pathway [path:hsa04012]; mTOR signaling pathway [path:hsa04150]; Insulin signaling pathway [path:hsa04910]; Adipocytokine signaling pathway [path:hsa04920]; Type II diabetes mellitus [path:hsa04930]; Glioma [path:hsa05214]; Prostate cancer [path:hsa05215]; Acute myeloid leukemia [path:hsa05221]
	ErbB signaling pathway [path:hsa04012]; mTOR signaling pathway [path:hsa04150]; TGF-beta signaling pathway [path:hsa04350]; Insulin signaling pathway [path:hsa04910]; Acute myeloid leukemia [path:hsa05221]
	Fc epsilon RI signaling pathway [path:hsa04664]; Chronic myeloid leukemia [path:hsa05220]
	Inositol phosphate metabolism [path:hsa00562]; ErbB signaling pathway [path:hsa04012]; Phosphatidylinositol signaling system [path:hsa04070]; mTOR signaling pathway [path:hsa04150]; Apoptosis [path:hsa04210]; VEGF signaling pathway [path:hsa04370]; Focal adhesion [path:hsa04510]; Toll-like receptor signaling pathway [path:hsa04620]; Jak-STAT signaling pathway [path:hsa04630]; Natural killer cell mediated cytotoxicity [path:hsa04650]; T cell receptor signaling pathway [path:hsa04660]; B cell receptor signaling pathway [path:hsa04662]; Fc epsilon RI signaling pathway [path:hsa04664]; Leukocyte transendothelial migration [path:hsa04670]; Regulation of actin cytoskeleton [path:hsa04810]; Insulin signaling pathway [path:hsa04910]; Type II diabetes mellitus [path:hsa04930]; Colorectal cancer [path:hsa05210]; Renal cell carcinoma [path:hsa05211]; Pancreatic cancer [path:hsa05212]; Endometrial cancer [path:hsa05213]; Glioma [path:hsa05214]; Prostate cancer [path:hsa05215]; Melanoma [path:hsa05218]; Chronic myeloid leukemia [path:hsa05220]; Acute myeloid leukemia [path:hsa05221]; Small cell lung cancer [path:hsa05222]; Non-small cell lung cancer [path:hsa05223]
	Inositol phosphate metabolism [path:hsa00562]; Phosphatidylinositol signaling system [path:hsa04070]; [path:hsa04115]; Focal adhesion [path:hsa04510]; Tight junction [path:hsa04530]; Endometrial cancer [path:hsa05213]; Glioma [path:hsa05214]; Prostate cancer [path:hsa05215]; Melanoma [path:hsa05218]; Small cell lung cancer [path:hsa05222]
	Insulin signaling pathway [path:hsa04910]; Adipocytokine signaling pathway [path:hsa04920]; Type II diabetes mellitus [path:hsa04930]
	Jak-STAT signaling pathway [path:hsa04630]
	Jak-STAT signaling pathway [path:hsa04630]; Adipocytokine signaling pathway [path:hsa04920]; Pancreatic cancer [path:hsa05212]; Acute myeloid leukemia [path:hsa05221]
	MAPK signaling pathway [path:hsa04010]; Apoptosis [path:hsa04210]; Toll-like receptor signaling pathway [path:hsa04620]; T cell receptor signaling pathway [path:hsa04660]; B cell receptor signaling pathway [path:hsa04662]; Adipocytokine signaling pathway [path:hsa04920]; Epithelial cell signaling in Helicobacter pylori infection [path:hsa05120]; Pancreatic cancer [path:hsa05212]; Prostate cancer [path:hsa05215]; Chronic myeloid leukemia [path:hsa05220]; Acute myeloid leukemia [path:hsa05221]; Small cell lung cancer [path:hsa05222]

	MAPK signaling pathway [path:hsa04010]; Cell cycle [path:hsa04110]; [path:hsa04115]; Apoptosis [path:hsa04210]; Wnt signaling pathway [path:hsa04310]; Amyotrophic lateral sclerosis (ALS) [path:hsa05030]; Huntington's disease [path:hsa05040]; Colorectal cancer [path:hsa05210]; Pancreatic cancer [path:hsa05212]; Endometrial cancer [path:hsa05213]; Glioma [path:hsa05214]; Prostate cancer [path:hsa05215]; Thyroid cancer [path:hsa05216]; Basal cell carcinoma [path:hsa05217]; Melanoma [path:hsa05218]; Bladder cancer [path:hsa05219]; Chronic myeloid leukemia [path:hsa05220]; Small cell lung cancer [path:hsa05222]; Non-small cell lung cancer [path:hsa05223]
	MAPK signaling pathway [path:hsa04010]; ErbB signaling pathway [path:hsa04012]; Calcium signaling pathway [path:hsa04020]; Phosphatidylinositol signaling system [path:hsa04070]; Wnt signaling pathway [path:hsa04310]; VEGF signaling pathway [path:hsa04370]; Focal adhesion [path:hsa04510]; Tight junction [path:hsa04530]; Gap junction [path:hsa04540]; Natural killer cell mediated cytotoxicity [path:hsa04650]; Fc epsilon RI signaling pathway [path:hsa04664]; Leukocyte transendothelial migration [path:hsa04670]; Long-term potentiation [path:hsa04720]; Long-term depression [path:hsa04730]; GnRH signaling pathway [path:hsa04912]; Melanogenesis [path:hsa04916]; Cholera [path:hsa05110]; Pathogenic Escherichia coli infection [path:hsa05130]; [path:hsa05131]; Glioma [path:hsa05214]; Non-small cell lung cancer [path:hsa05223]
	MAPK signaling pathway [path:hsa04010]; ErbB signaling pathway [path:hsa04012]; Dorso-ventral axis formation [path:hsa04320]; VEGF signaling pathway [path:hsa04370]; Focal adhesion [path:hsa04510]; Gap junction [path:hsa04540]; Toll-like receptor signaling pathway [path:hsa04620]; Natural killer cell mediated cytotoxicity [path:hsa04650]; Fc epsilon RI signaling pathway [path:hsa04664]; Long-term potentiation [path:hsa04720]; Long-term depression [path:hsa04730]; Regulation of actin cytoskeleton [path:hsa04810]; Insulin signaling pathway [path:hsa04910]; GnRH signaling pathway [path:hsa04912]; Melanogenesis [path:hsa04916]; Colorectal cancer [path:hsa05210]; Renal cell carcinoma [path:hsa05211]; Pancreatic cancer [path:hsa05212]; Endometrial cancer [path:hsa05213]; Glioma [path:hsa05214]; Prostate cancer [path:hsa05215]; Thyroid cancer [path:hsa05216]; Melanoma [path:hsa05218]; Bladder cancer [path:hsa05219]; Chronic myeloid leukemia [path:hsa05220]; Acute myeloid leukemia [path:hsa05221]; Non-small cell lung cancer [path:hsa05223]
	MAPK signaling pathway [path:hsa04010]; ErbB signaling pathway [path:hsa04012]; Wnt signaling pathway [path:hsa04310]; TGF-beta signaling pathway [path:hsa04350]; Jak-STAT signaling pathway [path:hsa04630]; Colorectal cancer [path:hsa05210]; Endometrial cancer [path:hsa05213]; Thyroid cancer [path:hsa05216]; Bladder cancer [path:hsa05219]; Chronic myeloid leukemia [path:hsa05220]; Acute myeloid leukemia [path:hsa05221]; Small cell lung cancer [path:hsa05222]
	MAPK signaling pathway [path:hsa04010]; ErbB signaling pathway [path:hsa04012]; mTOR signaling pathway [path:hsa04150]; Apoptosis [path:hsa04210]; VEGF signaling pathway [path:hsa04370]; Focal adhesion [path:hsa04510]; Tight junction [path:hsa04530]; Toll-like receptor signaling pathway [path:hsa04620]; Jak-STAT signaling pathway [path:hsa04630]; T cell receptor signaling pathway [path:hsa04660]; B cell receptor signaling pathway [path:hsa04662]; Fc epsilon RI signaling pathway [path:hsa04664]; Insulin signaling pathway [path:hsa04910]; Adipocytokine signaling pathway [path:hsa04920]; Colorectal cancer [path:hsa05210]; Renal cell carcinoma [path:hsa05211]; Pancreatic cancer [path:hsa05212]; Endometrial cancer [path:hsa05213]; Glioma [path:hsa05214]; Prostate cancer [path:hsa05215]; Melanoma [path:hsa05218]; Chronic myeloid leukemia [path:hsa05220]; Acute myeloid leukemia [path:hsa05221]; Small cell lung cancer [path:hsa05222]; Non-small cell lung cancer [path:hsa05223]
	MAPK signaling pathway [path:hsa04010]; ErbB signaling pathway [path:hsa04012]; mTOR signaling pathway [path:hsa04150]; Dorso-ventral axis formation [path:hsa04320]; Focal adhesion [path:hsa04510]; Natural killer cell mediated cytotoxicity [path:hsa04650]; Long-term potentiation [path:hsa04720]; Long-term depression [path:hsa04730]; Regulation of actin cytoskeleton [path:hsa04810]; Insulin signaling pathway [path:hsa04910]; Colorectal cancer [path:hsa05210]; Renal cell carcinoma [path:hsa05211]; Pancreatic cancer [path:hsa05212]; Endometrial cancer [path:hsa05213]; Glioma [path:hsa05214]; Prostate cancer [path:hsa05215]; Thyroid cancer [path:hsa05216]; Melanoma [path:hsa05218]; Bladder cancer [path:hsa05219]; Chronic myeloid leukemia [path:hsa05220]; Acute myeloid leukemia [path:hsa05221]; Non-small cell lung cancer [path:hsa05223]

	MAPK signaling pathway [path:hsa04010]; ErbB signaling pathway [path:hsa04012]; mTOR signaling pathway [path:hsa04150]; Dorso-ventral axis formation [path:hsa04320]; TGF-beta signaling pathway [path:hsa04350]; Axon guidance [path:hsa04360]; VEGF signaling pathway [path:hsa04370]; Focal adhesion [path:hsa04510]; Adherens junction [path:hsa04520]; Gap junction [path:hsa04540]; Toll-like receptor signaling pathway [path:hsa04620]; Natural killer cell mediated cytotoxicity [path:hsa04650]; Fc epsilon RI signaling pathway [path:hsa04664]; Long-term potentiation [path:hsa04720]; Long-term depression [path:hsa04730]; Regulation of actin cytoskeleton [path:hsa04810]; Insulin signaling pathway [path:hsa04910]; GnRH signaling pathway [path:hsa04912]; Melanogenesis [path:hsa04916]; Type II diabetes mellitus [path:hsa04930]; Colorectal cancer [path:hsa05210]; Renal cell carcinoma [path:hsa05211]; Pancreatic cancer [path:hsa05212]; Endometrial cancer [path:hsa05213]; Glioma [path:hsa05214]; Prostate cancer [path:hsa05215]; Thyroid cancer [path:hsa05216]; Melanoma [path:hsa05218]; Bladder cancer [path:hsa05219]; Chronic myeloid leukemia [path:hsa05220]; Acute myeloid leukemia [path:hsa05221]; Non-small cell lung cancer [path:hsa05223]
	MAPK signaling pathway [path:hsa04010]; VEGF signaling pathway [path:hsa04370]; Toll-like receptor signaling pathway [path:hsa04620]; Fc epsilon RI signaling pathway [path:hsa04664]; Leukocyte transendothelial migration [path:hsa04670]; GnRH signaling pathway [path:hsa04912]; Epithelial cell signaling in Helicobacter pylori infection [path:hsa05120]
	Natural killer cell mediated cytotoxicity [path:hsa04650]; T cell receptor signaling pathway [path:hsa04660]
	Neurodegenerative Diseases [path:hsa01510]; [path:hsa04115]; Apoptosis [path:hsa04210]; Amyotrophic lateral sclerosis (ALS) [path:hsa05030]; Colorectal cancer [path:hsa05210]
	Neurodegenerative Diseases [path:hsa01510]; [path:hsa04115]; Apoptosis [path:hsa04210]; Toll-like receptor signaling pathway [path:hsa04620]; Huntington's disease [path:hsa05040]; Dentatorubropallidoluysian atrophy (DRPLA) [path:hsa05050]
	Neurodegenerative Diseases [path:hsa01510]; Apoptosis [path:hsa04210]; Alzheimer's disease [path:hsa05010]; Dentatorubropallidoluysian atrophy (DRPLA) [path:hsa05050]
	Neurodegenerative Diseases [path:hsa01510]; Apoptosis [path:hsa04210]; Focal adhesion [path:hsa04510]; Amyotrophic lateral sclerosis (ALS) [path:hsa05030]; Colorectal cancer [path:hsa05210]; Prostate cancer [path:hsa05215]; Small cell lung cancer [path:hsa05222]
	Neurodegenerative Diseases [path:hsa01510]; Apoptosis [path:hsa04210]; Jak-STAT signaling pathway [path:hsa04630]; Amyotrophic lateral sclerosis (ALS) [path:hsa05030]; Pancreatic cancer [path:hsa05212]; Chronic myeloid leukemia [path:hsa05220]; Small cell lung cancer [path:hsa05222]
	Neurodegenerative Diseases [path:hsa01510]; ErbB signaling pathway [path:hsa04012]; Apoptosis [path:hsa04210]; VEGF signaling pathway [path:hsa04370]; Focal adhesion [path:hsa04510]; Insulin signaling pathway [path:hsa04910]; Amyotrophic lateral sclerosis (ALS) [path:hsa05030]; Colorectal cancer [path:hsa05210]; Pancreatic cancer [path:hsa05212]; Endometrial cancer [path:hsa05213]; Prostate cancer [path:hsa05215]; Melanoma [path:hsa05218]; Chronic myeloid leukemia [path:hsa05220]; Acute myeloid leukemia [path:hsa05221]; Non-small cell lung cancer [path:hsa05223]
	PPAR signaling pathway [path:hsa03320]; Thyroid cancer [path:hsa05216]
	Phosphatidylinositol signaling system [path:hsa04070]; B cell receptor signaling pathway [path:hsa04662]; Fc epsilon RI signaling pathway [path:hsa04664]; Insulin signaling pathway [path:hsa04910]
	Ribosome [path:hsa03010]; mTOR signaling pathway [path:hsa04150]; Insulin signaling pathway [path:hsa04910]
	T cell receptor signaling pathway [path:hsa04660]; Fc epsilon RI signaling pathway [path:hsa04664]
	TGF-beta signaling pathway [path:hsa04350]
	Tight junction [path:hsa04530]; Fc epsilon RI signaling pathway [path:hsa04664]; GnRH signaling pathway [path:hsa04912]; Type II diabetes mellitus [path:hsa04930]

	Toll-like receptor signaling pathway [path:hsa04620]; Jak-STAT signaling pathway [path:hsa04630]; Pancreatic cancer [path:hsa05212]
	Wnt signaling pathway [path:hsa04310]; Focal adhesion [path:hsa04510]; Adherens junction [path:hsa04520]; Tight junction [path:hsa04530]; Leukocyte transendothelial migration [path:hsa04670]; Melanogenesis [path:hsa04916]; Pathogenic Escherichia coli infection [path:hsa05130]; [path:hsa05131]; Colorectal cancer [path:hsa05210]; Endometrial cancer [path:hsa05213]; Prostate cancer [path:hsa05215]; Thyroid cancer [path:hsa05216]; Basal cell carcinoma [path:hsa05217]
	mTOR signaling pathway [path:hsa04150]; Renal cell carcinoma [path:hsa05211]

SUPPLEMENTARY FIGURE LEGENDS

Supplemental Figure 1. Purity of CD34+ and CD34+CD38- selection. Sequential testing of a single sample after CD34 sorting and then pre and post CD38+ depletion is shown. Staining of unstained cells and isotype controls are shown on the left. The purity of the CD34- (top) and CD34+ (bottom) population after CD34+ selection . The CD34- population was only stained with CD34+ antibody, the CD34+ population was stained for CD34 and CD38 before undergoing CD38+ depletion. The histograms (2nd column from right) show the dual populations pre staining and the uniformity of staining in the CD38- population post depletion. The rightmost figure shows the staining results for the stem cell population.

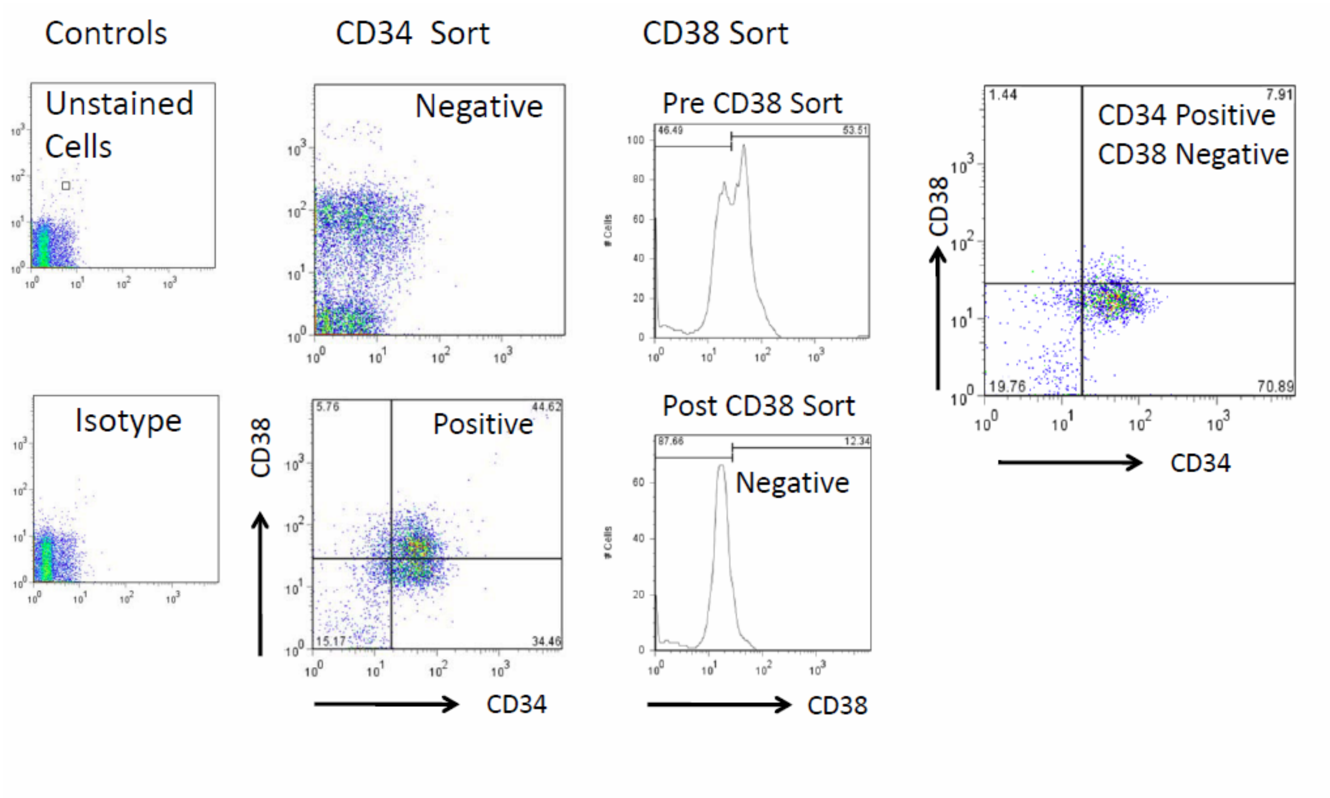

Supplemental Figure 2. Two way hierarchical clustering of paried differences C) CD34+ vs. BULK cells,D) CD34+ vs. CD34- and E) CD34+CD38+ vs. CD34+CD38 between using normalized data. Colors in the heatmap represent log ratios of protein expression in paired sampels, with black representing 0, pure red representing +3, and pure green representing -3. Data beyond these bounds was truncated for display purposes. Comparable figures for -A) Stem Cells and Bulk cells and B)Stem Cells and CD34+, cells are shown as figure 2A and B.

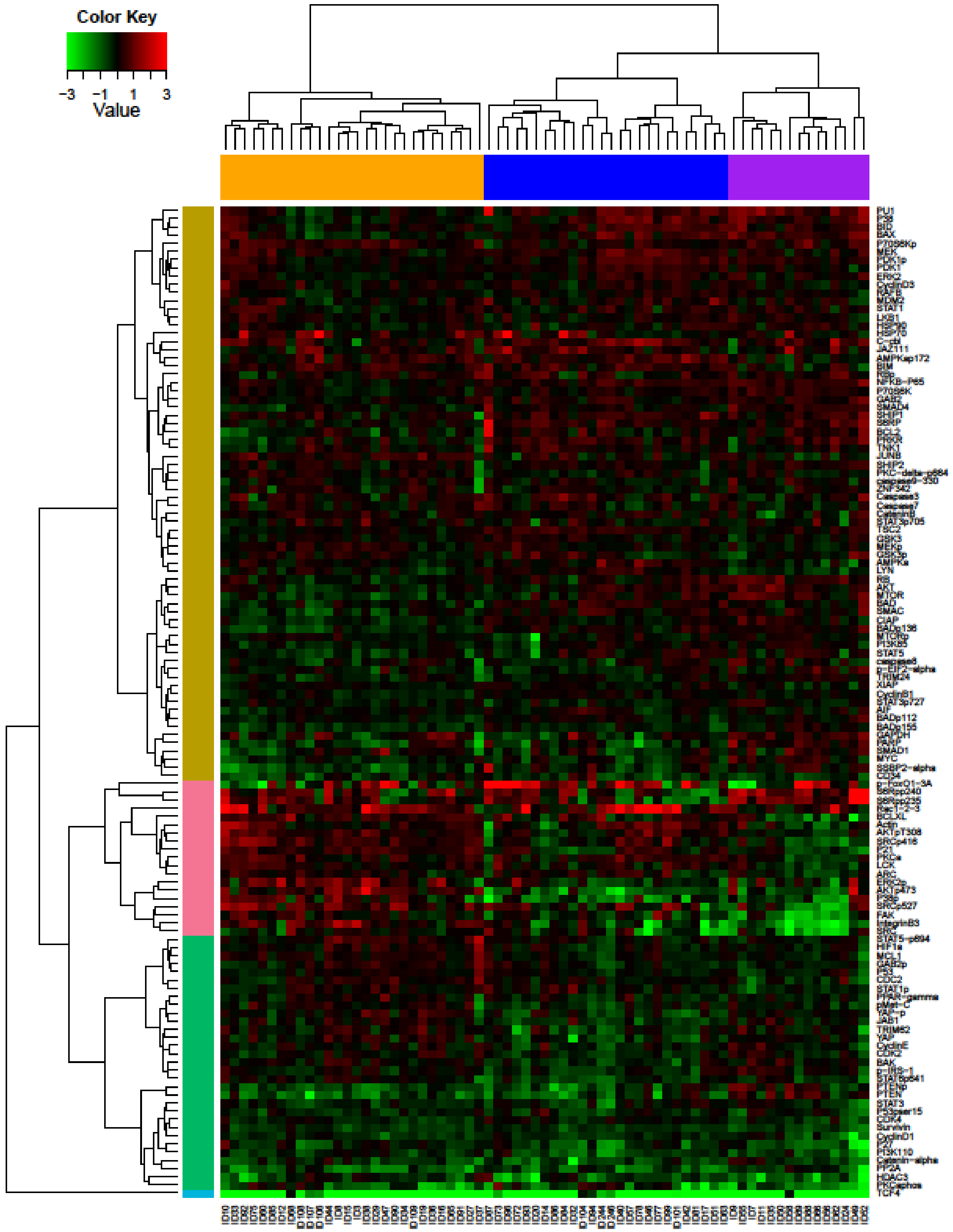
Color Key
−3 −1 1 3
Value

Supplemental Figure 2D. Comparison of CD34+ vs. CD34.

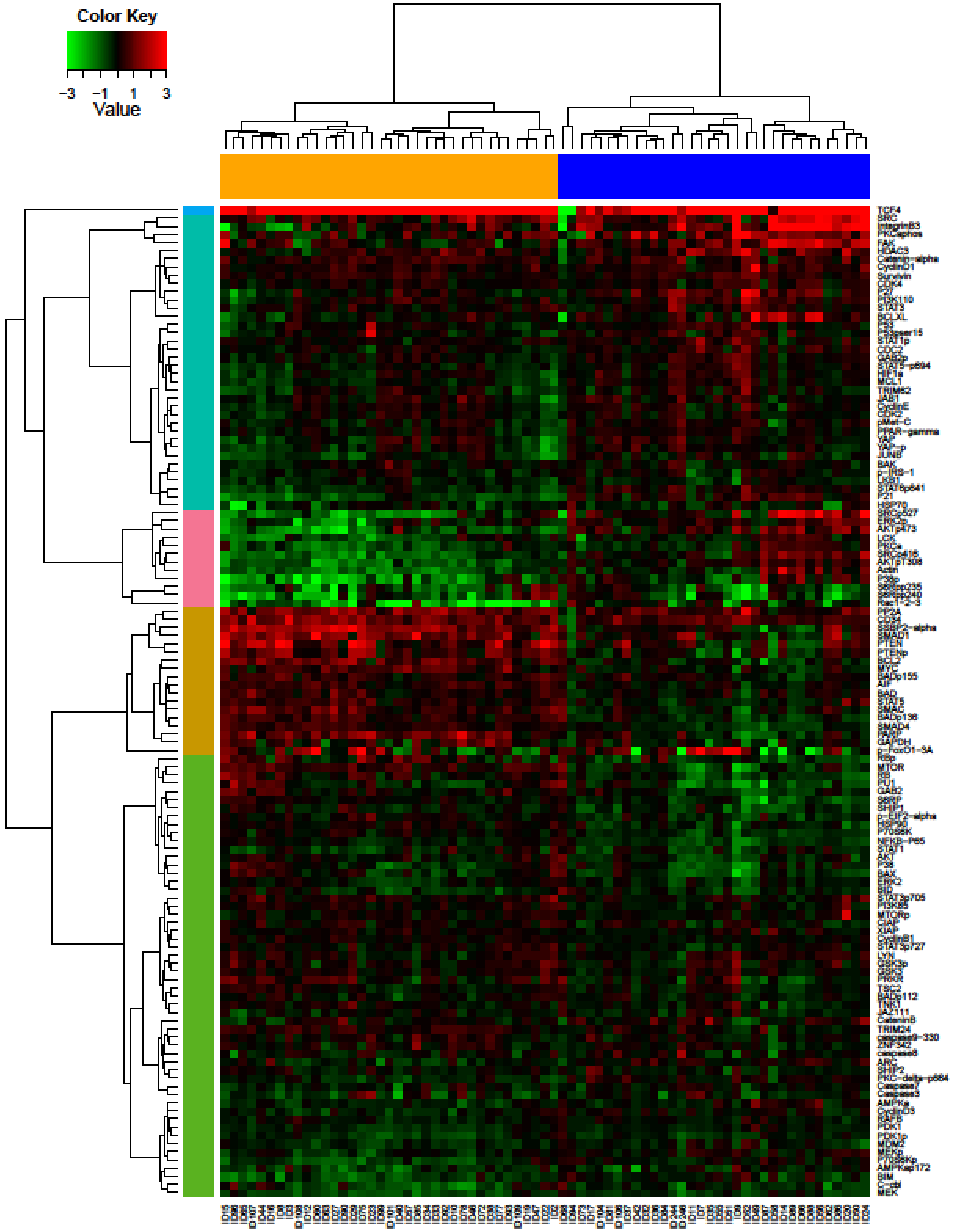

Supplemental Figure 2E. Comparison of CD34+Cd38- vs. CD34+Cd38+

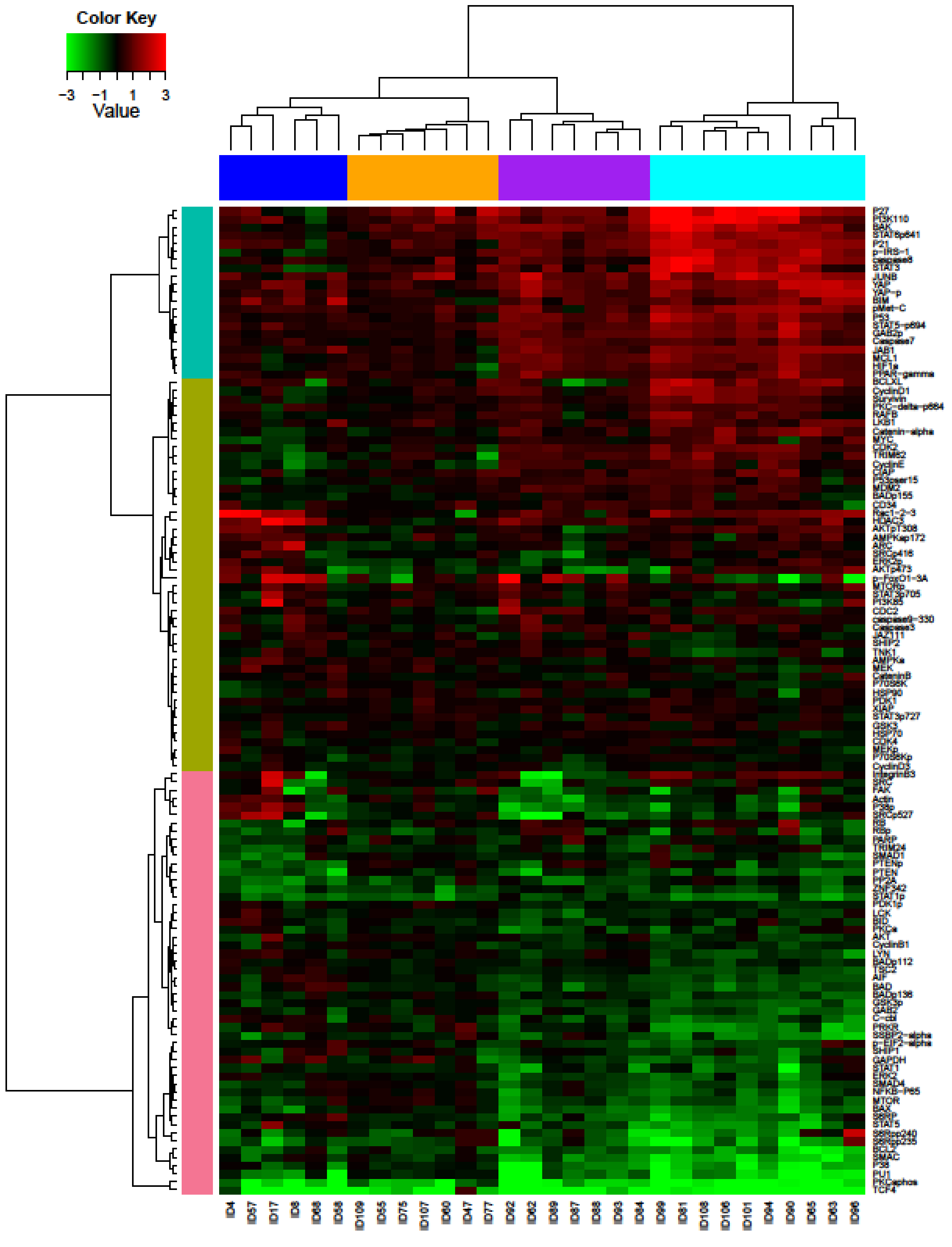

Supplemental Figure 3. Cluster analysis on patients grouped by individual protein pathways demonstrates that patients coalesced into two or three different clusters.

(A) Apoptosis Proteins

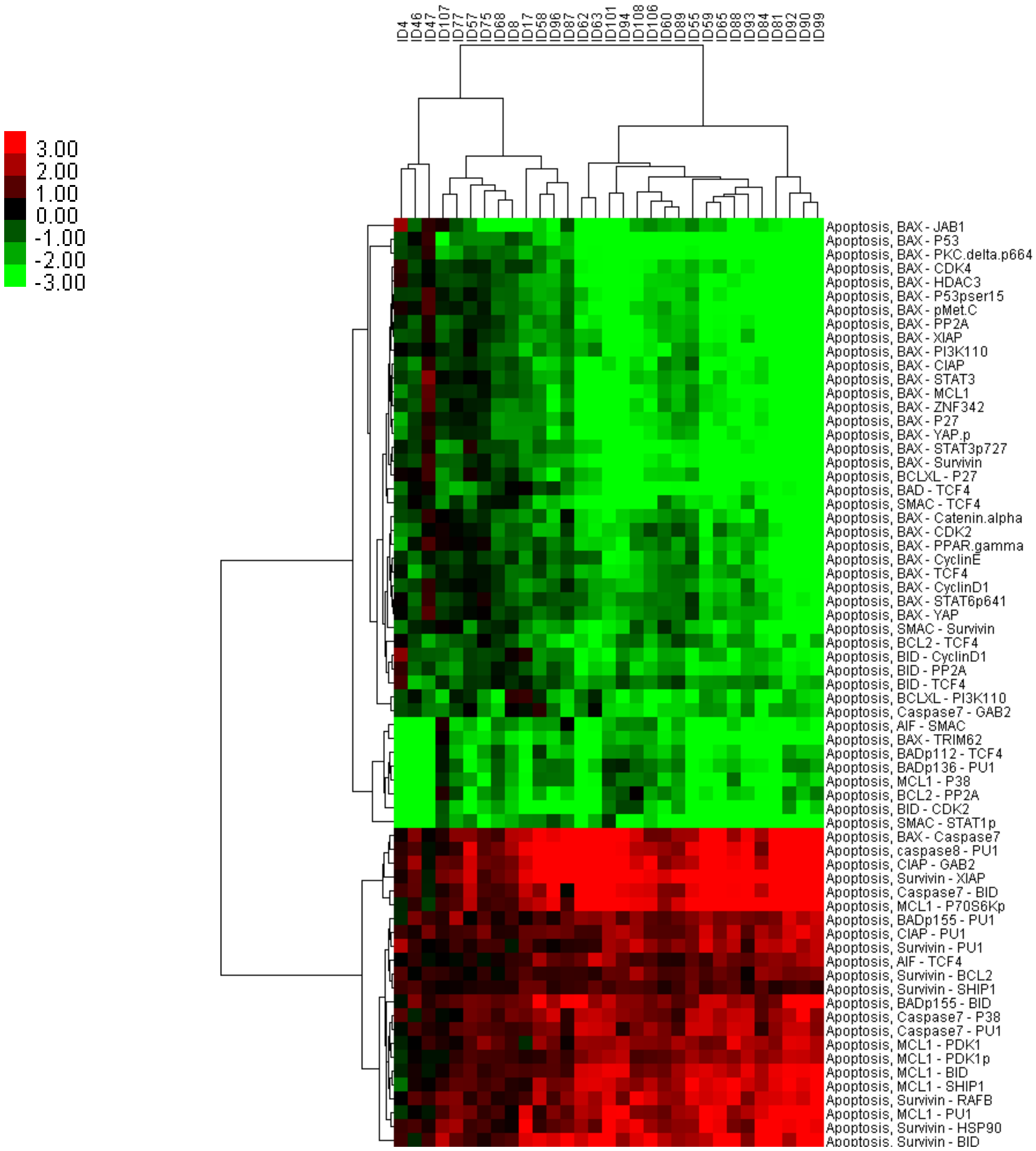

(B) MAPK Pathway Proteins

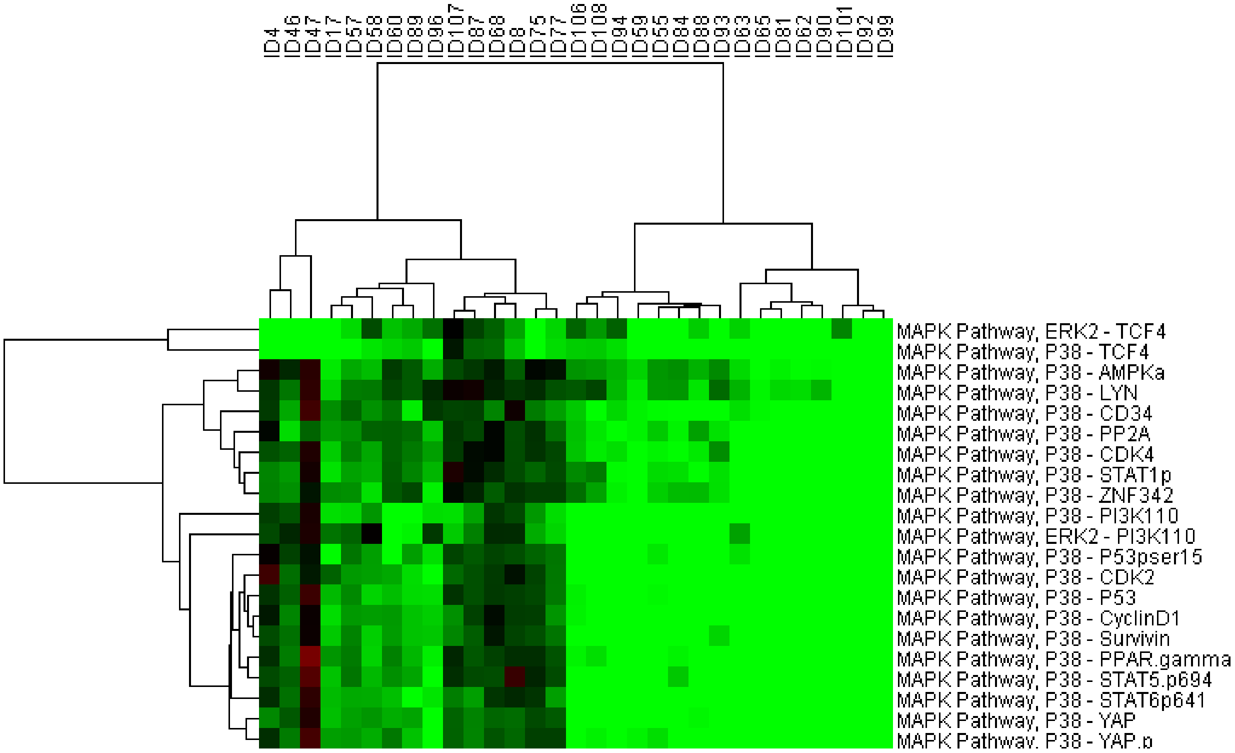

(C) Metabolic Proteins

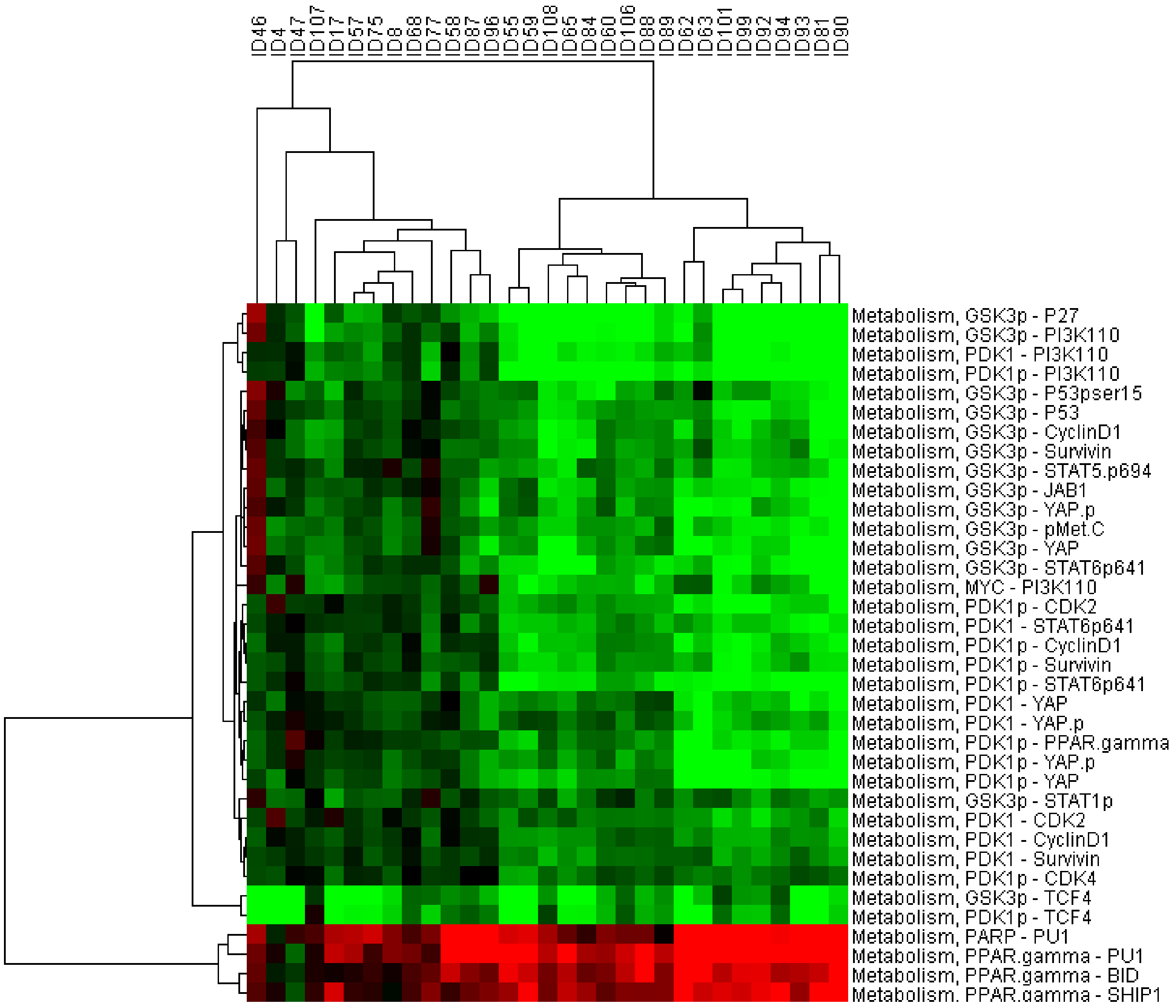

(D) Proliferation, Protein Synthesis, Tumor Suppressor, WNT, Stress Response Proteins

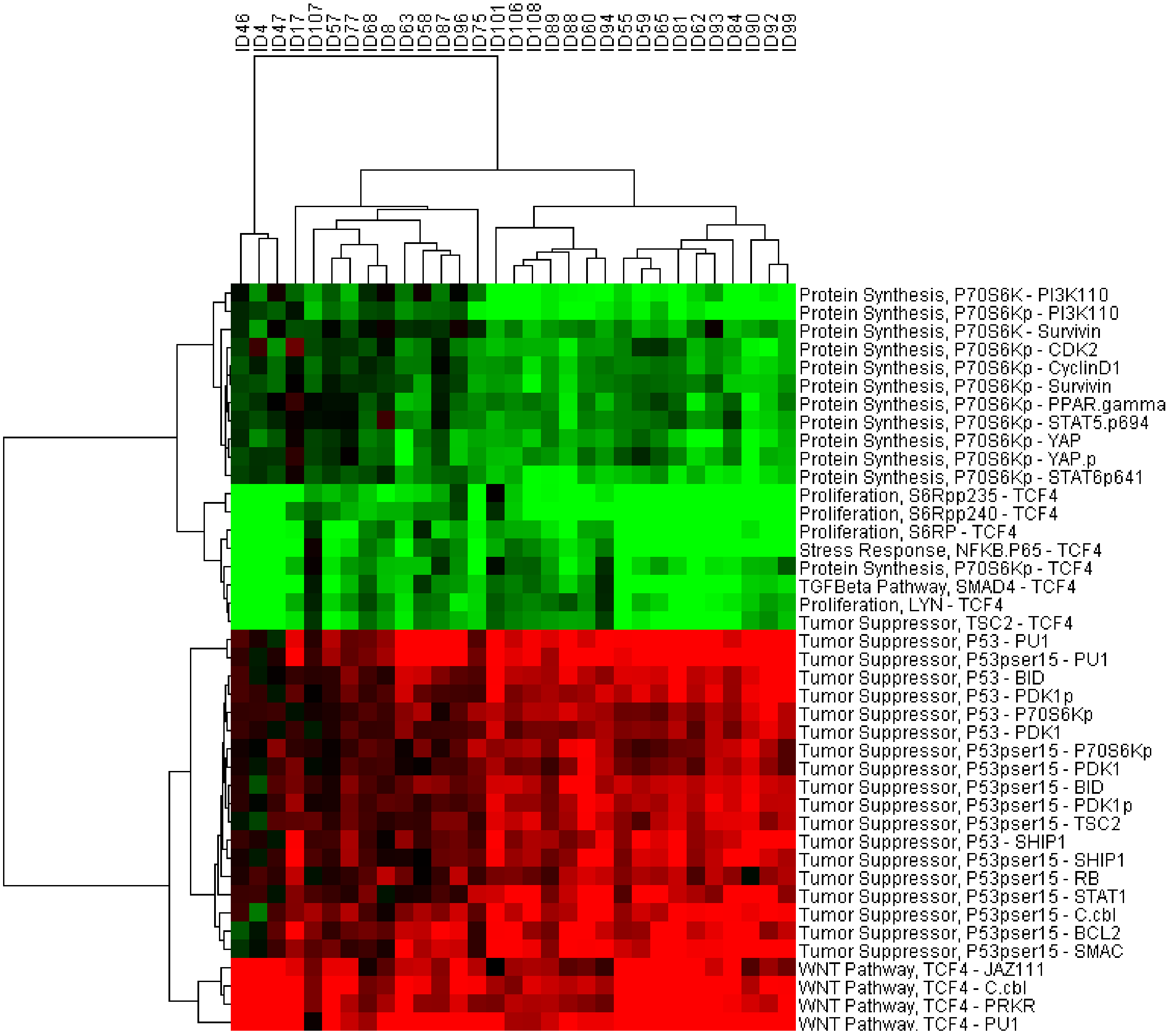

(E) PI3K Pathway Proteins

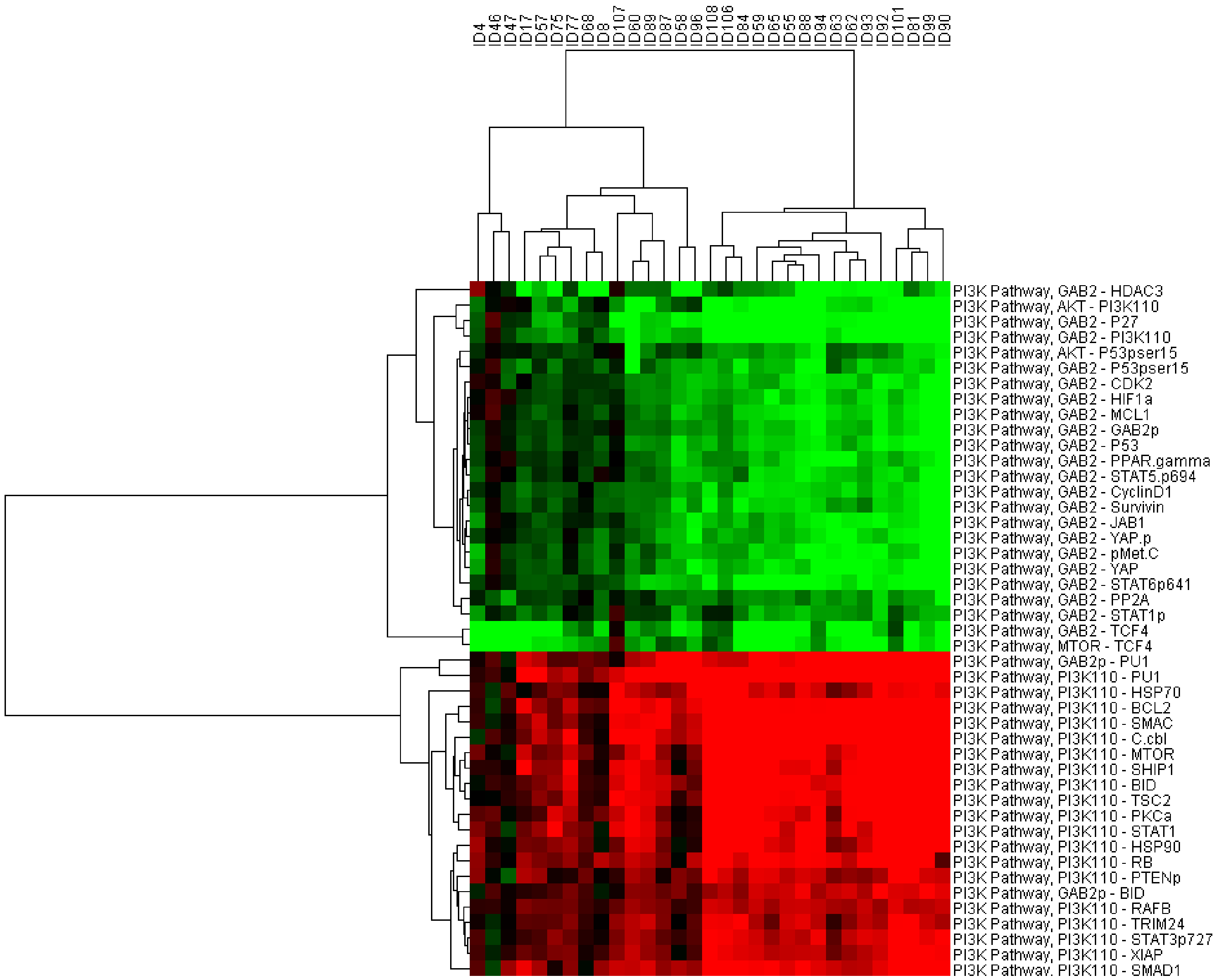

(F) Cell Cycle Proteins

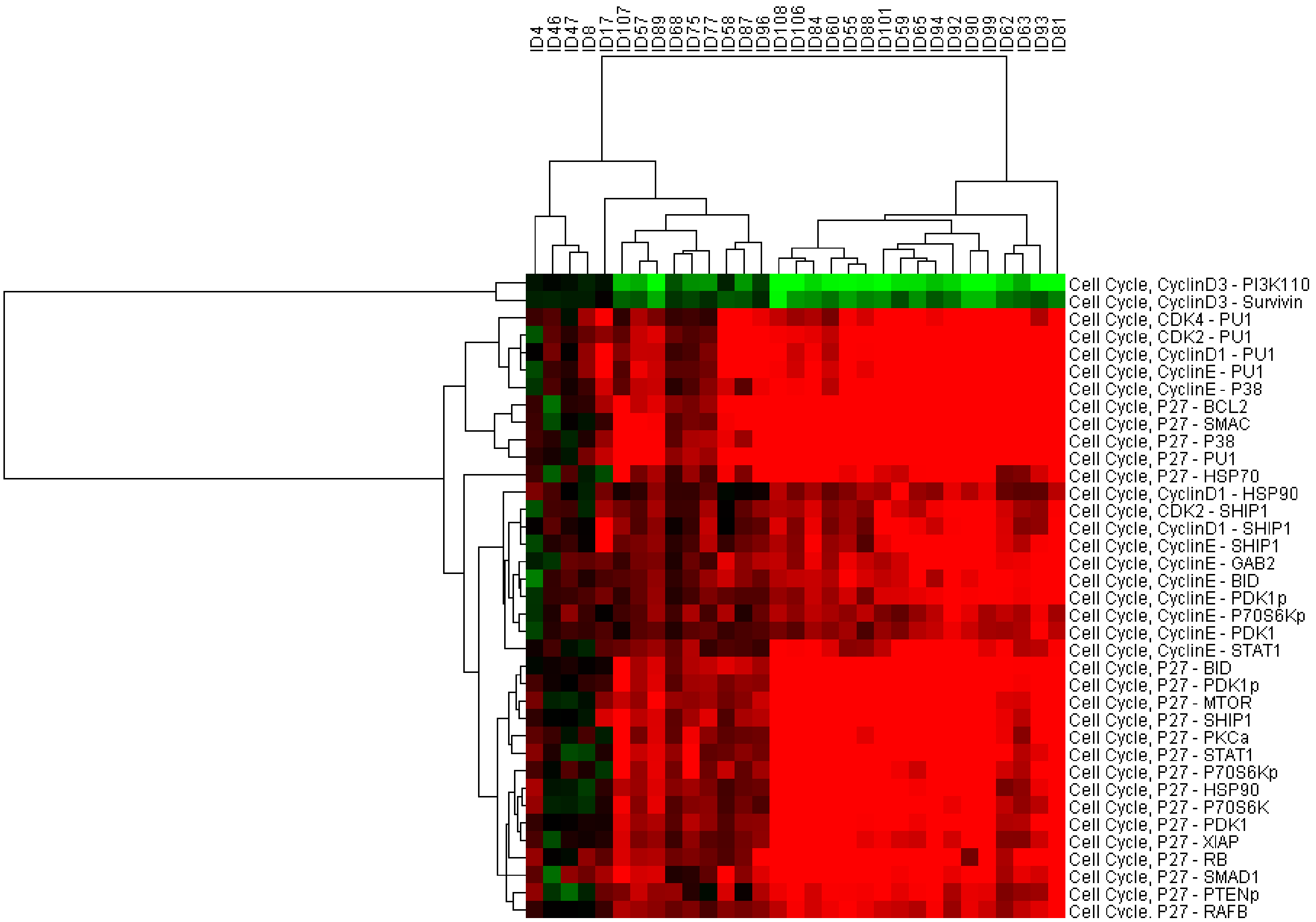

(G) Cytoskeletal, Hypoxia, and Wound-Healing Proteins

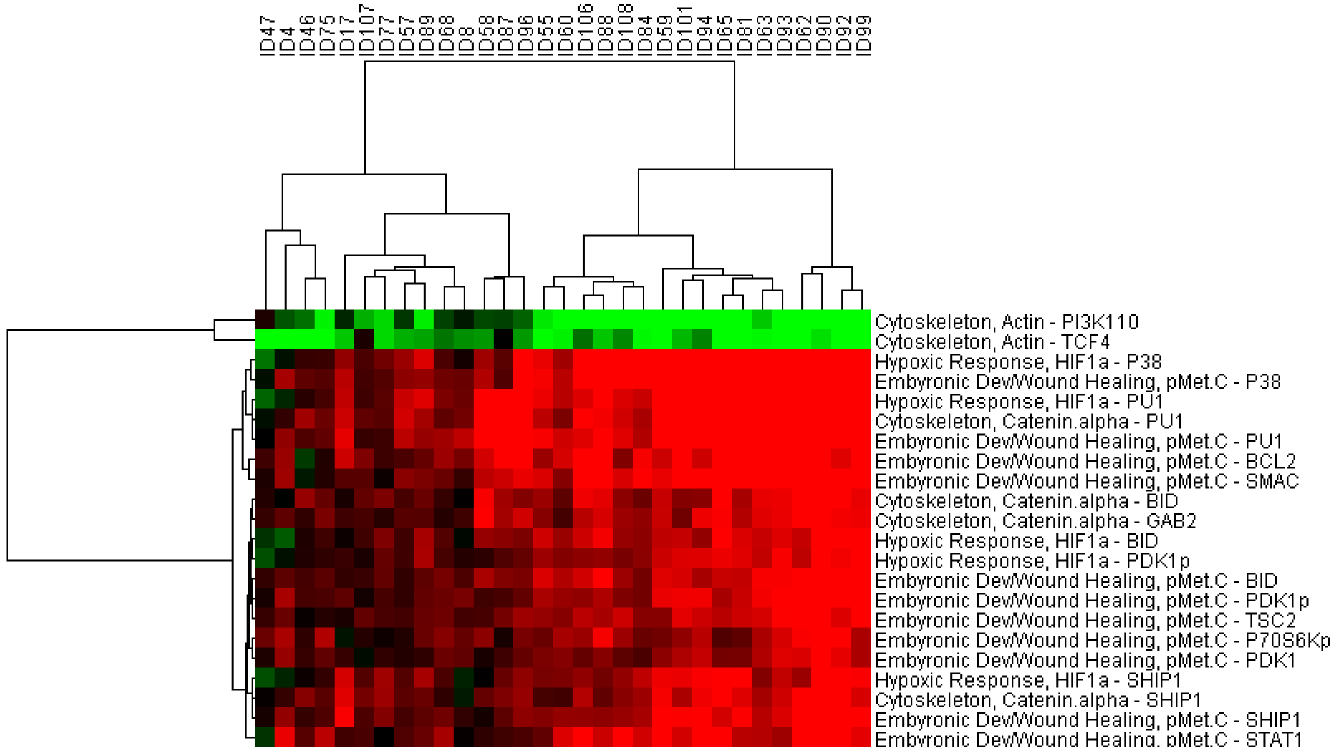

(H) JAK/STAT Pathway Proteins

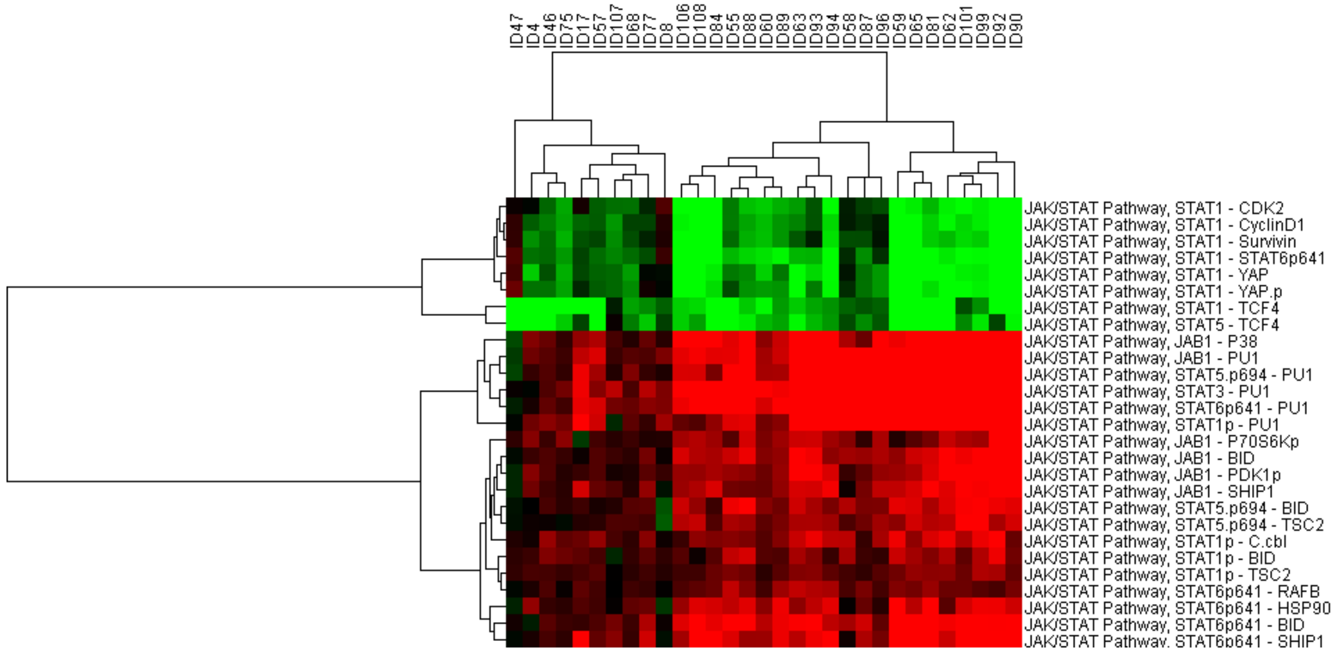

(I) Other Proteins

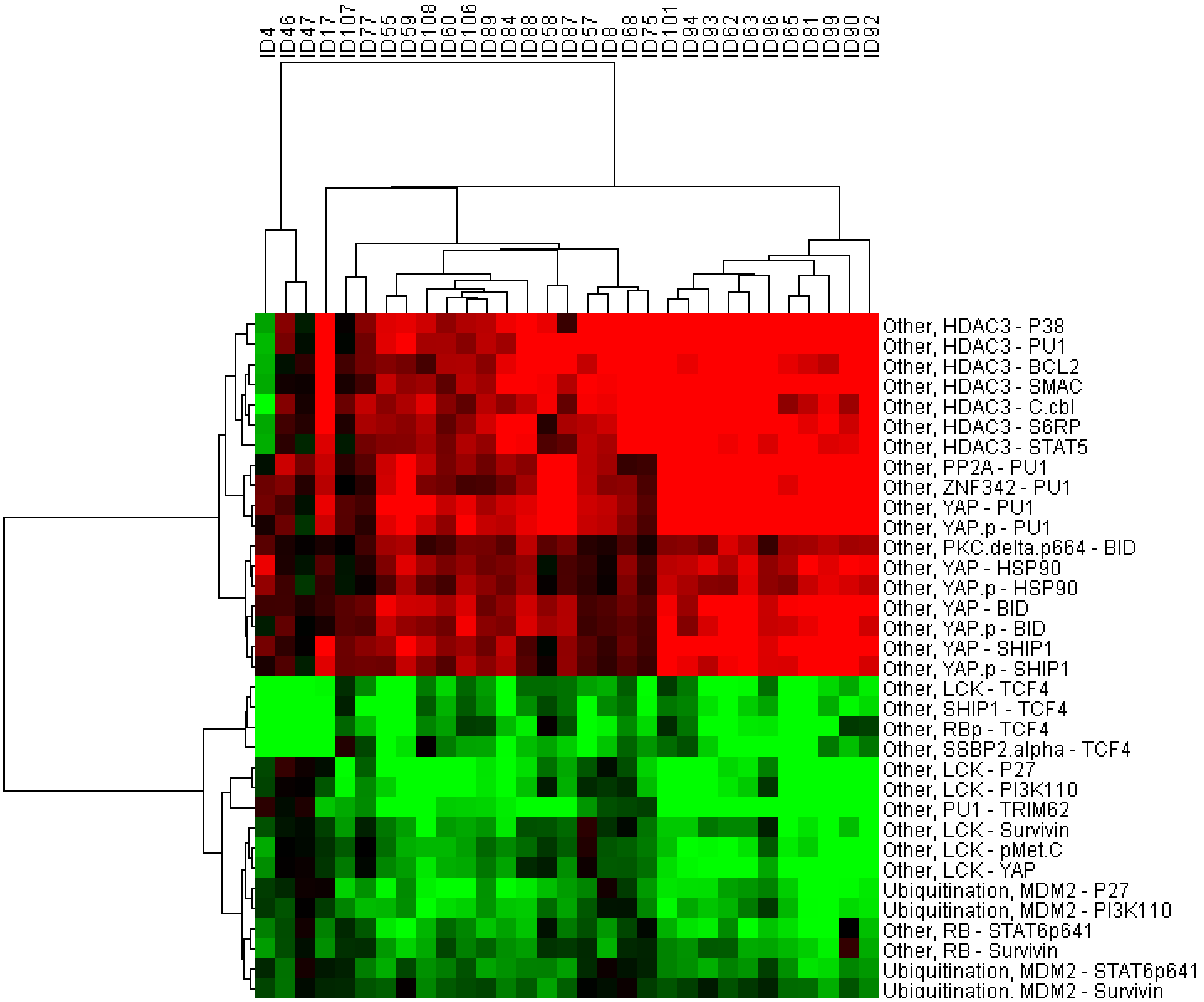

Supplemental Figure 4. Subnetworks of highly interconnected proteins found from modularity analysis are highlighted by a yellow ellipse and the associated proteins are listed in the accompanying table. The most highly connected proteins in each group (≥ 4 degrees, from patient dataset) are also listed. The green to red edges represent relative expression levels from the RPPA data, and show connectivity predicted by the t-test protein pair comparisons between Stem vs. Bulk cells. Edge width corresponds to relative p-values, with wider edges having lower p-values. Blue edges indicate PPI and signaling interactions from public databases. Node border color and wdith correspond to the number of protein connections. A legend for the node colors, which correspond to known signaling pathways associated with the proteins from available public databases, is included as supplemental table 6.

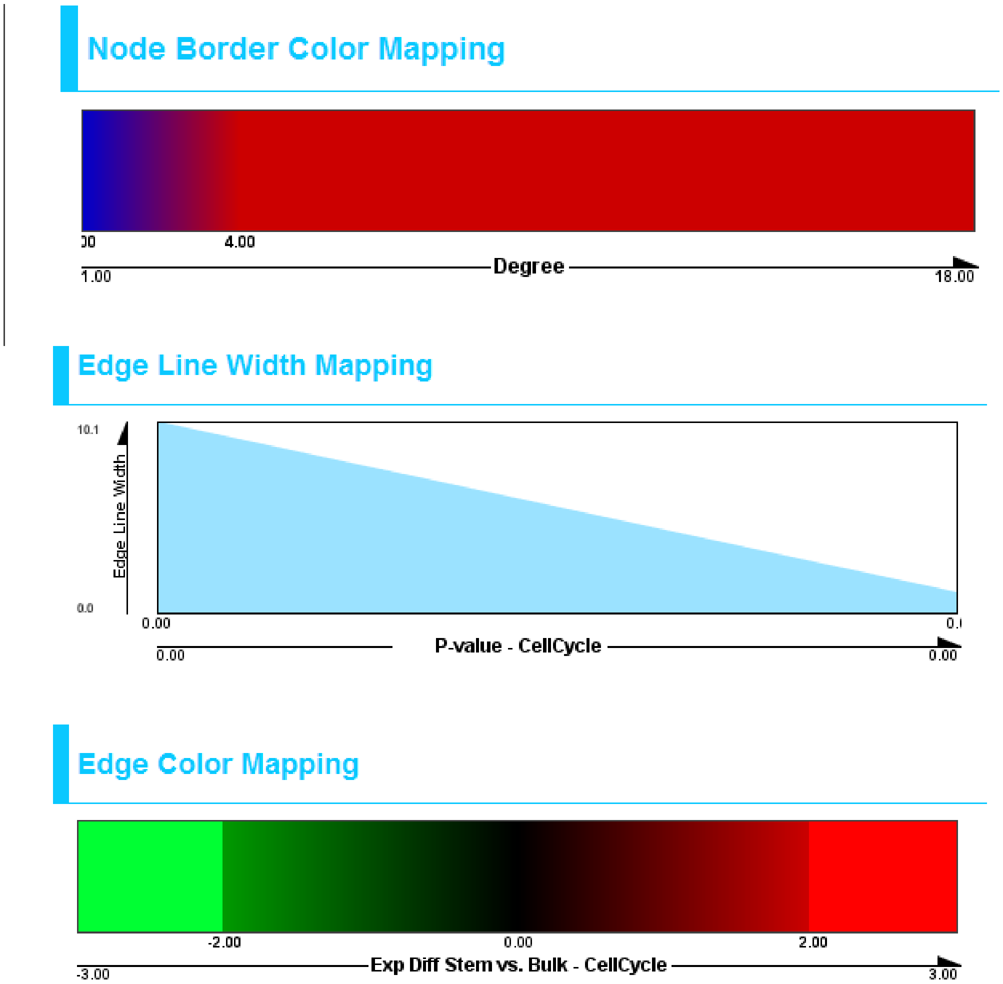

**Apoptosis Proteins**	
**Pathway Targets – Modularity Components**	**Highly Connected Individual Targets (Patients)**
**Module 1 (MIMI and Patient Data)**	BAX
BAX	BIRC5
BID	MCL1
CASP8	SPI1
BIRC5	BID
BIRC4	TCF4
BIRC2	CASP7
DIABLO	DIABLO
**Module 1 (Patient Data)**	
BAX	
BIRC5	
BIRC4	

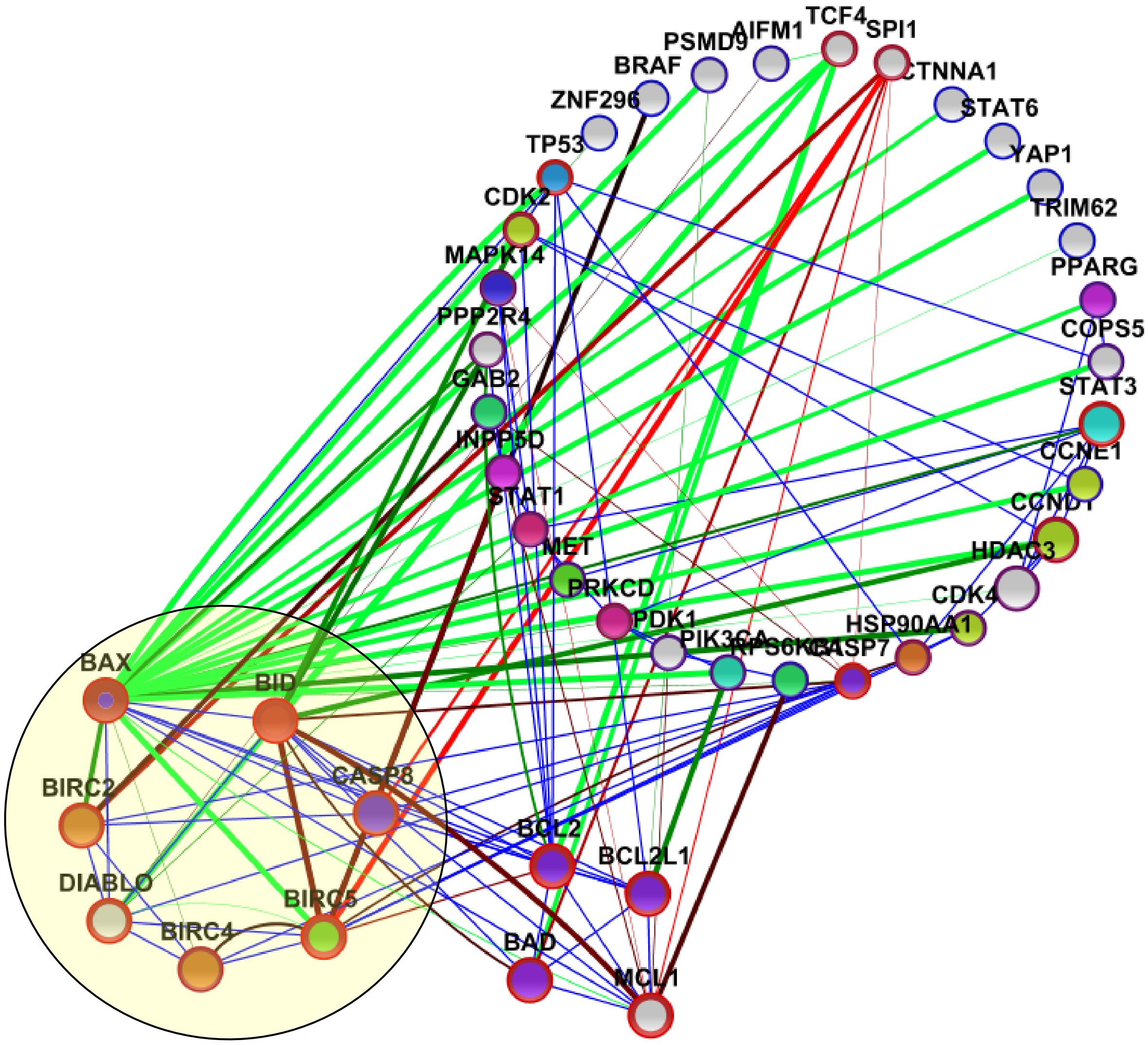

Cell Cycle Proteins	
**Pathway Targets – Modularity Components**	**Highly Connected Individual Targets (Patients)**
**Module 1 (MIMI and Patient Data)**	PSMD9
CCNE1	CCNE1
SPI1	SPI1
CDK4	INPP5D
CCND1	
CDK2	
BCL2	
PSMD9	
PRKCA	
HSPA1A	
**Module 1 (Patient Data)**	
None Found	

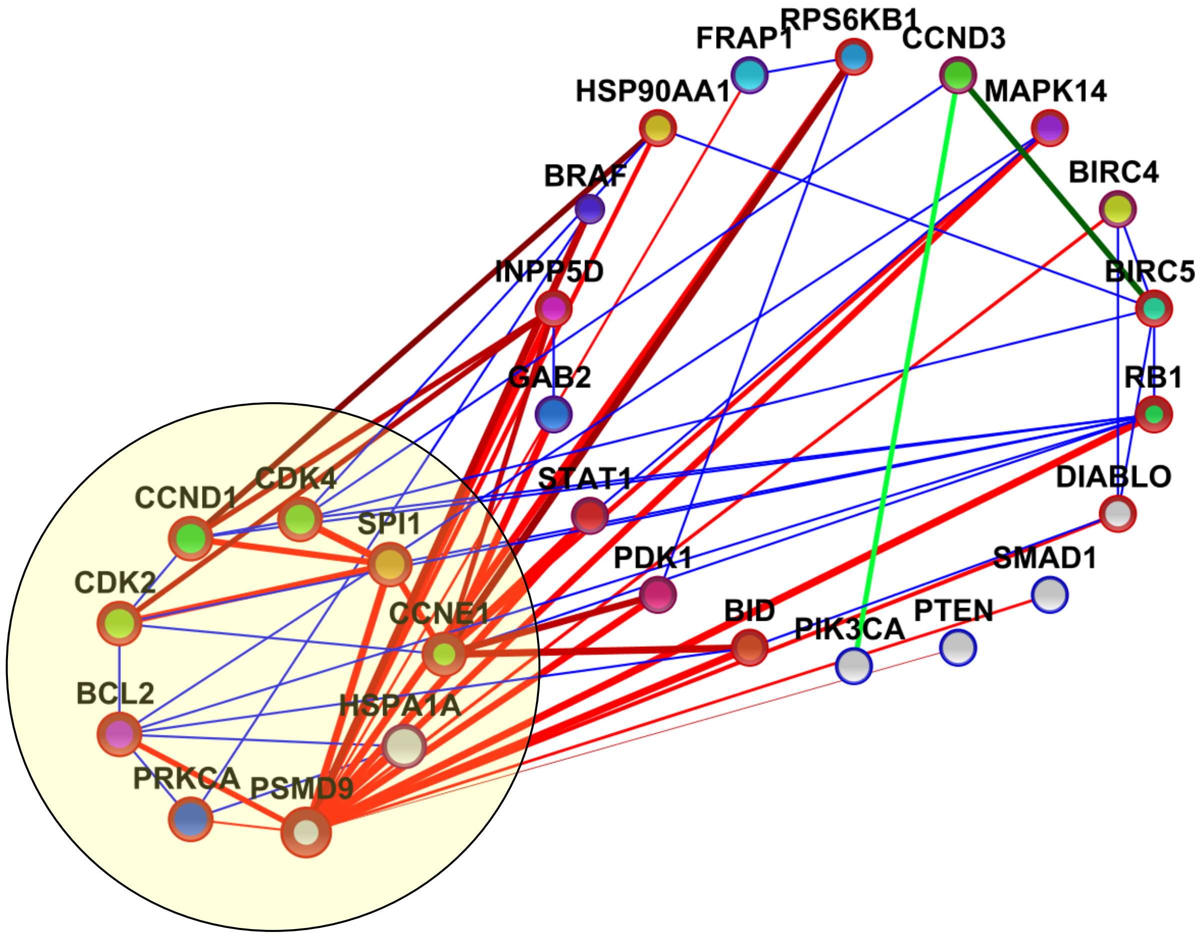

Cytoskeletal, Hypoxia, and Wound-Healing Proteins	
**Pathway Targets – Modularity Components**	**Highly Connected Individual Targets (Patients)**
**Module 1 (MIMI and Patient Data)**	MET
CTNNA1	HIF1A
INPP5D	CTNNA1
GAB2	
**Module 1 (Patient Data)**	
None Found	

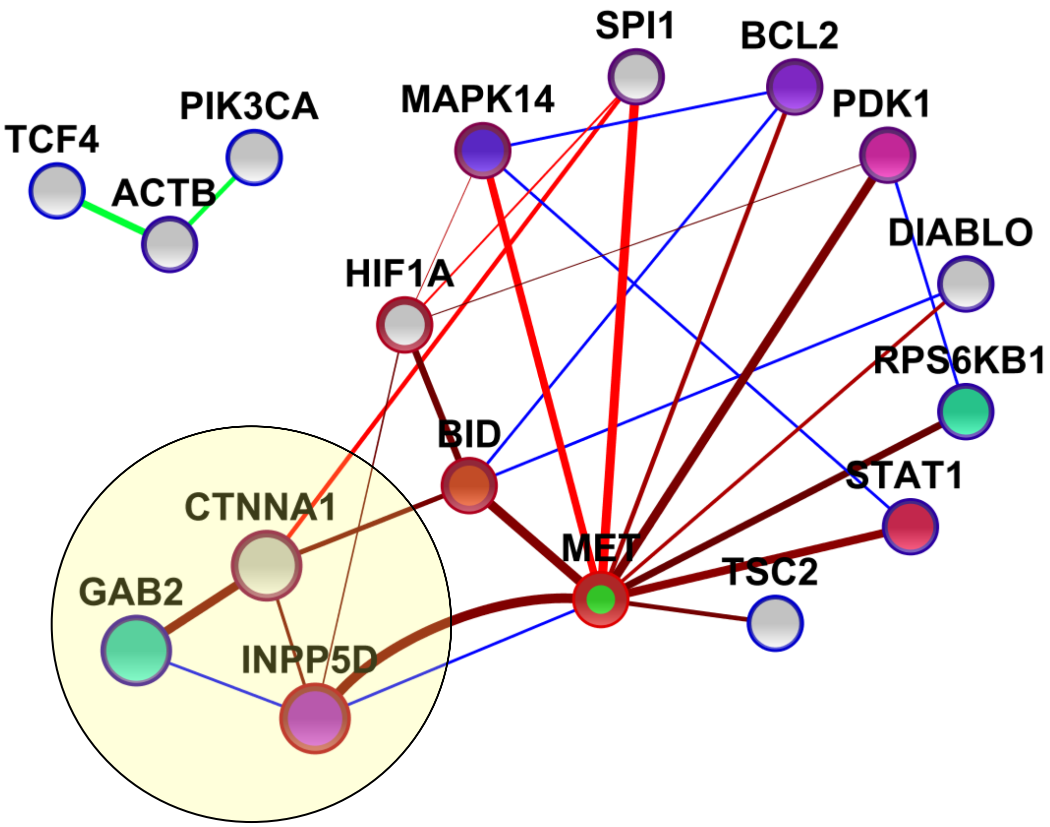

MAPK Pathway Proteins	
**Pathway Targets – Modularity Components**	**Highly Connected Individual Targets (Patients)**
**Module 1 (MIMI and Patient Data)**	MAPK14
MAPK14	
CDK4	
CDK2	
CCND1	
LYN	
BIRC5	
STAT1	
TP53	
**Module 1 (Patient Data)**	
None found	

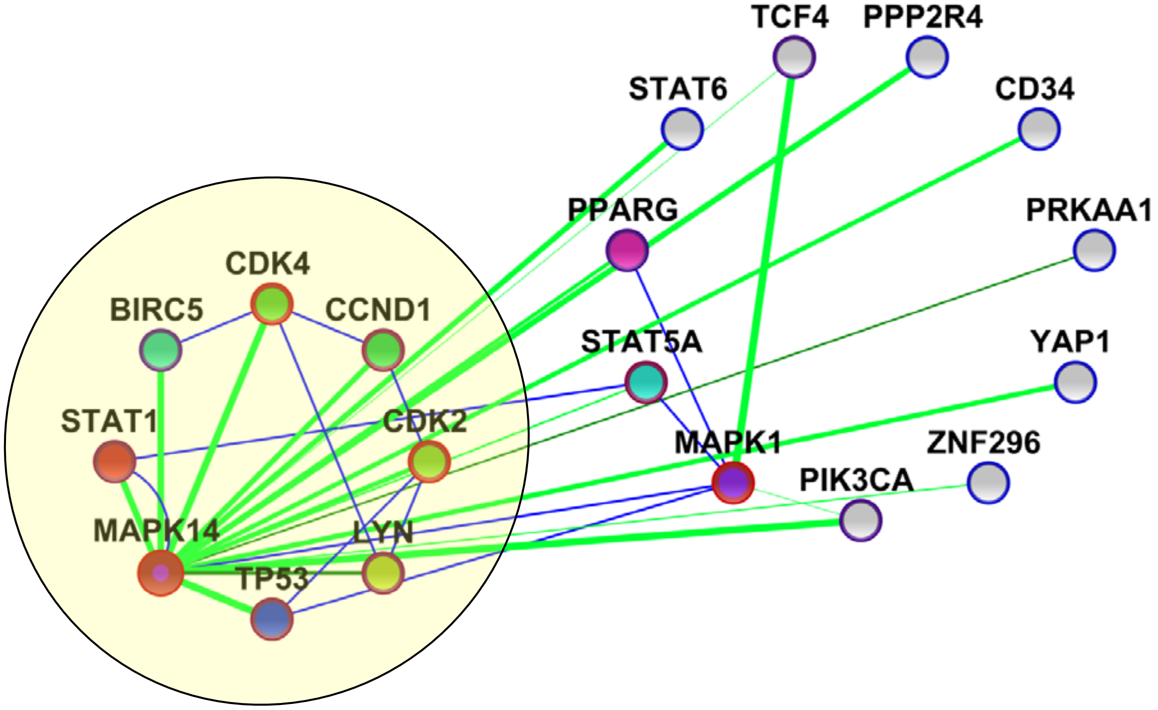

Proliferation and Stress Response Proteins	
**Pathway Targets – Modularity Components**	**Highly Connected Individual Targets (Patients)**
**Module 1 (MIMI and Patient Data)**	TP53
TP53	TCF4
BCL2	RSP6KB1
**Module 1 (Patient Data)**	
None found	

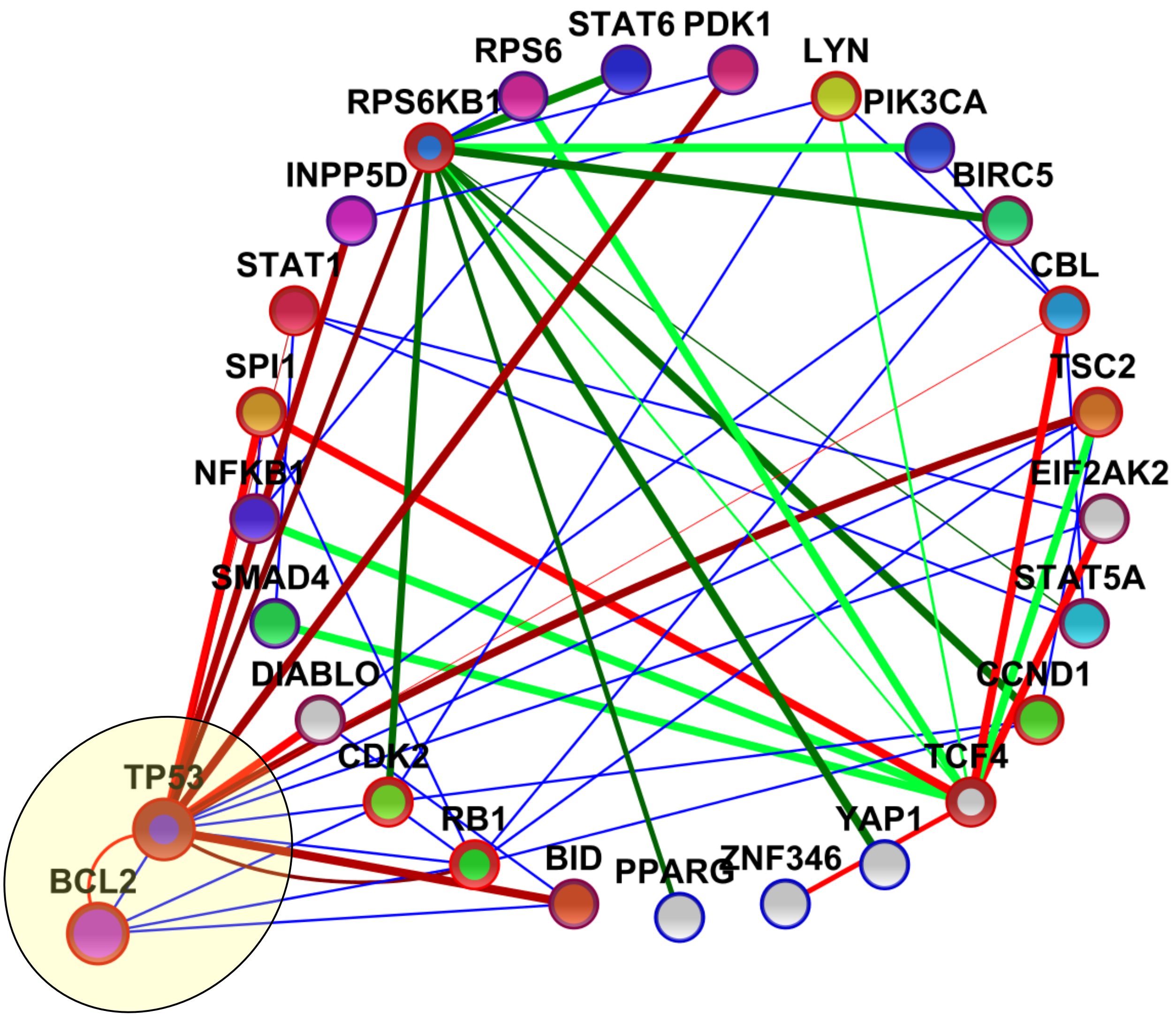

PI3K Pathway Proteins	
**Pathway Targets – Modularity Components**	**Highly Connected Individual Targets (Patients)**
**Module 1 (MIMI and Patient Data)**	PIK3CA
BCL2	GAB2
HSPA1A	
PRKCA	
**Module1 (Patient Data)**	
None Found	

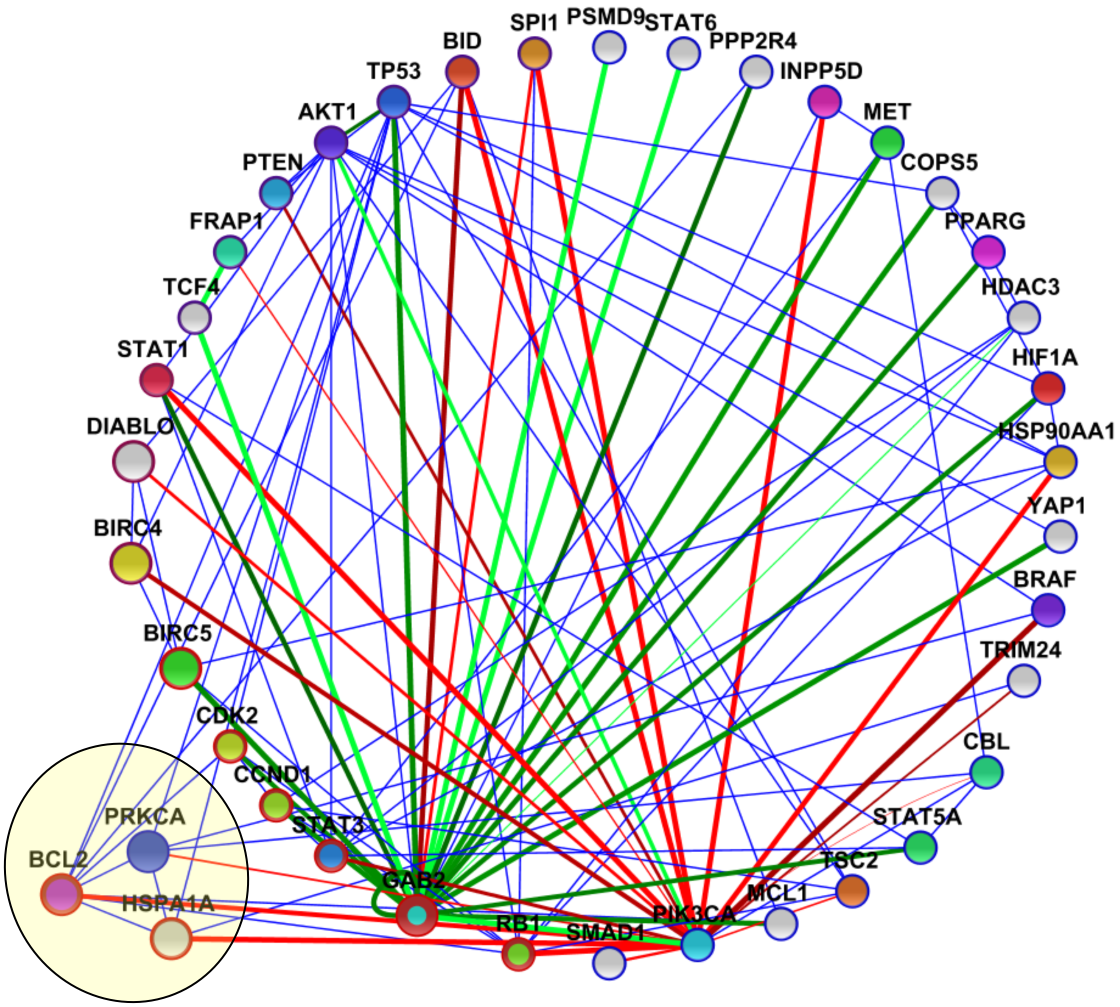

JAK/STAT Pathway Proteins	
**Pathway Targets – Modularity Components**	**Highly Connected Individual Targets (Patients)**
**Module 1 (MIMI and Patient Data)**	STAT1
STAT1	STAT6
STAT6	STAT5A
BID	SPI1
COPS5	BID
RPSKB1	COPS5
PDK1	
**Module 1 (Patient Data)**	
STAT1	
STAT6	
SPI1	
BID	

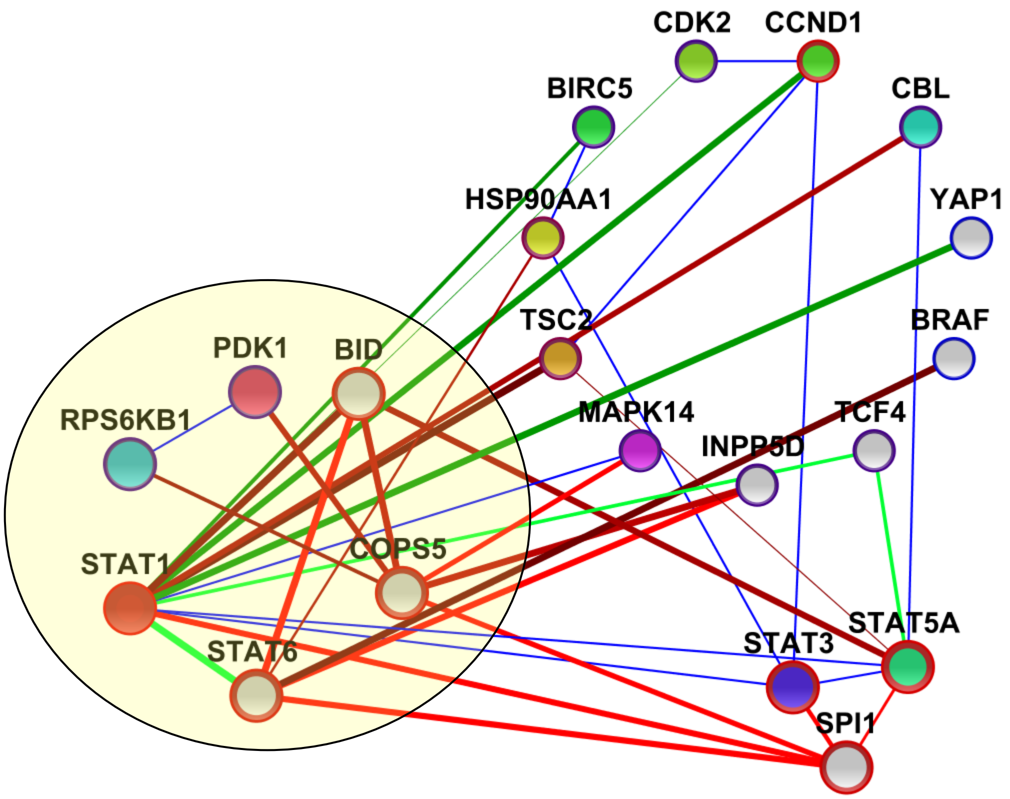

Metabolism Proteins	
**Pathway Targets – Modularity Components**	**Highly Connected Individual Targets (Patients)**
No submodules found	PDK1
	GSK3A

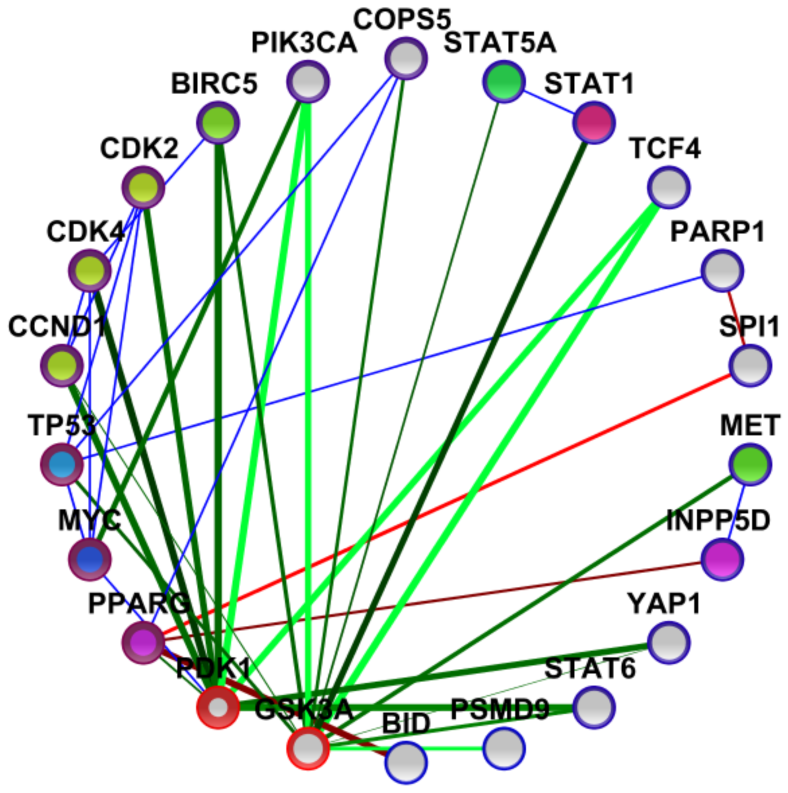

Other Proteins	
**Pathway Targets – Modularity Components**	**Highly Connected Individual Targets (Patients)**
**No submodules found**	HDAC3
	MDM2
	YAP1
	TCF4
	SP1
	LCK

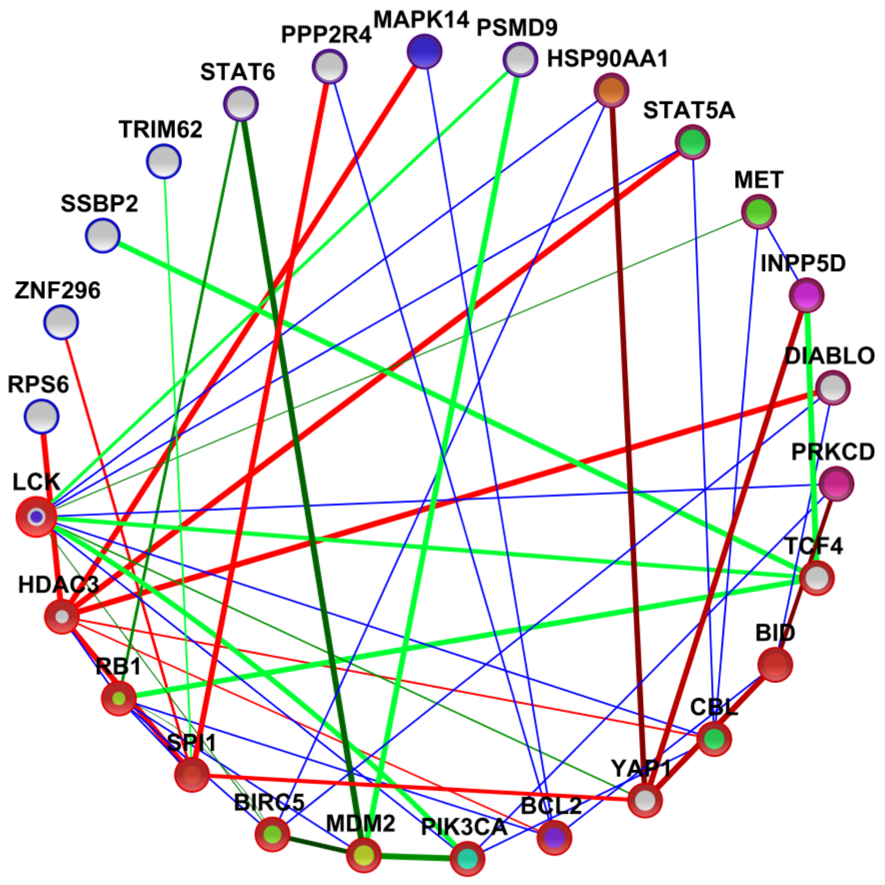

Supplement: File S1 — Contains all supplemental information for this manuscript in one location. Supplemental materials contains a comparison of RPPA and mRNA-GEP data. Table S1 contains the demographic and subset availability data for all cases. Table S2 lists the antibodies used in this study and details on primary and secondary antibody dilutions. Table S3 is a “Rosetta Stone” for the HUGO, Mimi and manufacturers antibody names . Table S4 lists the number of comparisons available between each subset. Table S5 lists proteins that were not different between LSC and bulk cells by protein function. Table S6 contains legends for the Cytoscape ™ figures. Figure S1 presents flow data showing the purity of the samples after sorting. Figure S2 is an expansion of regular Figure 2. It shows two way hierarchical clustering between C) CD34+ vs. Bulk cells, D) CD34+ vs. CD34- cells and E) Stem Cells and CD34+ cells. Figure S3 shows Network based clustering by protein functional category. Figure S4: Subnetworks for highly interconnected proteins for different cellular functions. (PDF) [file pone.0078453.s001.pdf]
